# What is the extent of a frequency-dependent social learning strategy space?

**DOI:** 10.1017/ehs.2022.11

**Published:** 2022-04-13

**Authors:** Aysha Bellamy, Ryan McKay, Sonja Vogt, Charles Efferson

**Affiliations:** 1 Department of Psychology, Royal Holloway, University of London, Egham, Surrey, TW20 0EX, UK; 2 Faculty of Business and Economics, University of Lausanne, 1015 Lausanne, Switzerland

**Keywords:** Social learning, frequency-dependent social learning, conformity, cultural evolution, social norms

## Abstract

Models of frequency-dependent social learning posit that individuals respond to the commonality of behaviours without additional variables modifying this. Such strategies bring important trade-offs, e.g. conformity is beneficial when observing people facing the same task but harmful when observing those facing a different task. Instead of rigidly responding to frequencies, however, social learners might modulate their response given additional information. To see, we ran an incentivised experiment where participants played either a game against nature or a coordination game. There were three types of information: (a) choice frequencies in a group of demonstrators; (b) an indication of whether these demonstrators learned in a similar or different environment; and (c) an indication about the reliability of this similarity information. Similarity information was either reliably correct, uninformative or reliably incorrect, where reliably correct and reliably incorrect treatments provided participants with equivalent earning opportunities. Participants adjusted their decision-making to all three types of information. Adjustments, however, were asymmetric, with participants doing especially well when conforming to demonstrators who were reliably similar to them. The overall response, however, was more fluid and complex than this one case. This flexibility should attenuate the trade-offs commonly assumed to shape the evolution of frequency-dependent social learning strategies.

## Graphical abstract

**Figure:**
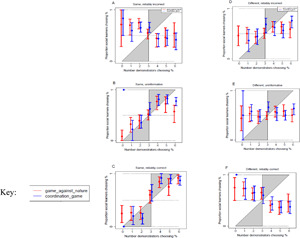

**Social media summary:** Frequency-dependent social learning strategies are flexible to at least three levels of social information.

## 1. Introduction

Social learning strategies help individuals exploit social information (Efferson et al., 2016; Mesoudi & Lycett, 2009; Molleman et al., 2013), and they also have profound effects on cultural evolutionary outcomes (Acerbi & Tehrani, 2018; Brady et al., 2020; Henrich, 2015; Henrich & Boyd, 2001; Henrich & Muthukrishna, 2021; Henrich et al., 2015; Lenfesty & Morgan, 2019; Molleman et al., 2014; Price &van Vugt, 2014). To complement this field, we investigate the flexibility – and complexity – of empirical frequency-dependent social learning strategies.

Frequency-dependent strategies mean that individuals choose behaviours based on how common or rare these behaviours are. The frequency-dependent strategy that has received the most attention is conformity (Efferson et al., 2016; Morgan & Laland, 2012; Morgan et al., 2019), which refers to a disproportionate response to frequencies. For example, if 75% of people braid their hair, a conformist will braid her hair with a probability greater than 0.75.

Conformity shapes cultural evolution (Chudek & Henrich, 2011; Henrich & Muthukrishna, 2021). Boyd and Richerson (1985) showed this with their seminal model. Individuals sampled three others in a group at random, and if these individuals were conformists, they disproportionately adopted the majority behaviour in the sample. The result was that people within groups became similar over time, but different groups could stabilise on different behaviours, norms and traditions. This model only allowed the social learners to respond to frequency information, which may give the impression that social learners conform as a kind of rule-of-thumb.

Individuals vary considerably in terms of their tendencies to conform (Efferson et al., 2008a; Muthukrishna et al., 2016), and for any given individual conformity is unlikely to be an indiscriminate rule-of-thumb. Individuals can switch between using frequency-dependent social learning and learning individually (Boyd & Richerson, 1985 (chapter 7); Mesoudi et al., 2015; Miu & Morgan, 2020; Reader, 2003). For example, Deffner et al. (2020) found that agents conformed in lieu of trial-and-error learning when migrating to a new group in a new environment. Individuals can also flexibly switch between frequency-dependent and payoff-based social learning strategies (McElreath et al., 2008; Molleman & Gächter, 2018). Some individuals only conform when uncertain (Kendal et al., 2018; Toelch et al., 2014).

This research shows that individuals are flexible when choosing between frequency-dependent social learning and other types of individual or social learning. We take a different approach. Conditional on the decision-maker responding to frequency information, we ask just how flexible individuals are in terms of adopting different frequency-dependent social learning strategies in different situations. Besides conformity, for example, individuals may exhibit linear strategies (Morgan & Laland, 2012), copy the minority (Efferson et al., 2008a; Evans et al., 2018) or adopt even more elaborate strategies. Rather than assuming a generic strategy, we examine both heterogeneity in strategies across individuals and, for any given individual, heterogeneity in the response to frequency-dependent information across situations.

An individual's preferred frequency-dependent social learning strategy may depend on the decision-making task. This paper focuses on asocial skills and social norms. Asocial skills are those where one's behaviour affects one's own payoff only, e.g. private tool use. We test this with a game against nature (see Section 2.2). In this case, choice frequencies provide useful information because the majority may choose a specific behaviour because they have learned that this behaviour is good for them (Barrett, 2015: Chapter 9). Social norms are rules that help people coordinate (Legare, 2017). We test this with a coordination game (Kets et al., 2021). Choice frequencies provide useful information because incentives favour behaving like those with whom one interacts (Legare, 2017; Wen et al., 2019).

With these two tasks, we investigate experimentally if and how social learners change their frequency-dependent social learning strategies based on information about the demonstrators from whom they learn. The available information includes (a) choice frequencies among demonstrators, (b) an indication that demonstrators are learning in a similar or different environment to the social learner and (c) whether this similarity information is reliable. We expect all participants to adjust to frequency-dependent social information, which we call ‘first-order’ social information. Even a simple rule-of-thumb like ‘always copy the majority’ implies a response to frequency information.

‘Second-order’ social information is a signal informing social learners if they are in an environment that is similar to, or different from, that of observed demonstrators. In general, similarity may hinge on physical traits like age (Shutts et al., 2010) or gender (Efferson et al., 2016). These kinds of traits may help a social learner identify who is facing a similar decision-making environment and thus a similar optimum choice. Similarity on external appearances, however, does not capture the full picture. An individual who has recently migrated to a new group could benefit from conforming to the local majority, even though they look different, precisely because they know the local optimum (Deffner et al., 2020; Henrich, 2015). In other words, similarity between social learners and demonstrators can have different meanings in different situations. If social learners can respond to this similarity, then this may attenuate certain trade-offs that have traditionally been associated with conformity. For example, conformity is assumed to be less beneficial around those with different optima (Efferson et al., 2016; Smaldino et al., 2017). The similarity signal is the ‘second-order’ social information which we also provide to participants.

In our study, third-order social information refers to the reliability of the similarity signal. The similarity signal, which informs social learners about the relation between their game and the game demonstrators are playing, can be reliably correct, uninformative or reliably incorrect. Reliably correct signals are equivalent to cases where we have reliable knowledge that we share a decision-making environment with someone else (Deffner et al., 2020).

A reliably incorrect signal is like a situation in which apparent similarity is negatively correlated with similarity in terms of the decision-making environment. Migrating to a new environment in which people look different is a case in point. The people may look different, but they know what to do in the local environment, not the recent immigrant.

To illustrate a reliably incorrect signal of similarity, consider that cooperative groups often employ painful or elaborate signals of group membership. Whilst these signals are hard to fake, the incentives for an outsider to do so are high (i.e. as they can deceptively extract resources for their own gain from an otherwise cooperative group; Smaldino et al., 2018; Sosis et al., 2007). The outsiders who fake signals of similarity are unlikely to be able to do so perfectly. Thus, any signals which do not exactly replicate a group's signal of membership may suggest that an outsider is faking similarity to exploit others. For example, if a cooperative group signals its membership via red clothing, then any individual wearing an off-shade of red is likely to be an outsider. Thus, the off-shade of red becomes a reliably incorrect signal of similarity (Stein et al., 2021). Indeed, Toelch et al. (2014) have shown that individuals switch between conformity and trial-and-error based on the perceived reliability of social information. Reliability of social information is clearly important, but ours is the first study to test the reliability of similarity signals as a third order of social information.

Finally, an uninformative signal means that apparent similarity is just as likely to mean a similar decision-making environment as it is to mean a dissimilar environment. When the signal of similarity is maximally uninformative in this way, conformity and any other social learning strategy yield the same expected payoff (Efferson et al., 2016). Thus, if a participant has any intrinsic preference for a specific response to frequency information, e.g. conformity, she can express this preference under an uninformative signal without any effect on her expected payoffs.

In sum, we test if and how participants adjust their frequency-dependent social learning strategies up to three orders of social information with both a game against nature and a coordination game. If participants respond in ways typical of many models (e.g. Boyd & Richerson, 1985; Perreault et al., 2012), the participant strategies depend on first-order information only. Otherwise, frequency-dependent strategies are more complex, more flexible and higher-dimensional than previously assumed.

Because this is, to our knowledge, the first investigation of social learning up to three orders of information, we do not predict the precise nature of adjustments. Broadly speaking, however, strategies should match one of the following possibilities:

1. Participants adjust to both second- and third-order social information, performing equally well on all informationally equivalent trials.
2. Participants adjust to both second- and third-order social information but show asymmetric adjustments across informationally equivalent settings. Put differently, participants perform better in some situations than in others, even though the situations have the same theoretical potential to make optimal choices owing to social learning.
3. Participants adjust to second-order social information (Efferson et al., 2016), but not third-order social information.
4. Social learners respond to first-order social information only.

## 2. Methods

### 2.1. Participants

A total of 302 subjects participated at the Centre for Experimental Social Sciences lab at FLAME University in Pune, India (mean age = 20.73, SD = 2.54, males = 88; see Appendix 1 for rationale and protocol). A power calculation supported this sample size (pre-registration at OSF | prereg_10Dec2018_bellamyEtAl.docx). The sample consisted of university students (see Ekuni et al., 2020), although university students may be more rich, democratic and educated (Henrich, 2020) than the rest of the Indian population. Two participants were international students. We used English for the experiment (see Andrade, 2009).

Participants were randomly assigned to play one of two roles. Sixty-six participants were demonstrators, who learned to play the games via the trial-and-error of individual learning. The remaining 236 participants were social learners, who could only learn via social information. Separating the participants into these roles may seem arbitrary as people ordinarily shift between individual and social learning (Miu et al., 2020). Crucially, however, this approach allowed us to isolate responses to social information without the typical challenges related to drawing causal inferences about social influence, social learning and peer effects (Angrist, 2014; Efferson et al., 2016; Manski, 2000).

### 2.2. The games

**Figure 1.**
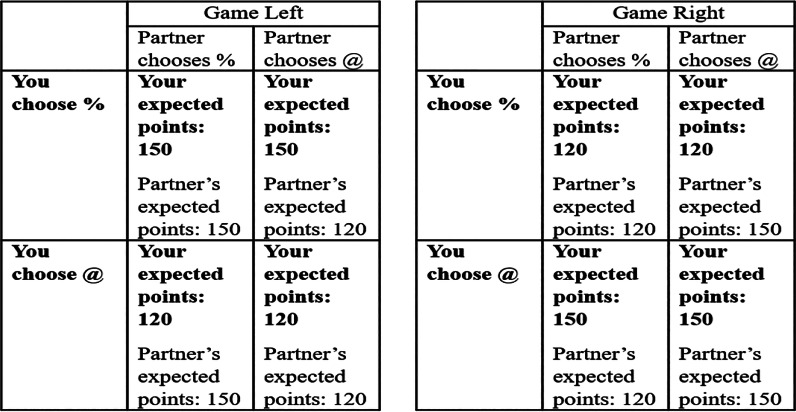
The payoff matrix shown to participants for the game against nature. Text in bold represents the expected payoffs for the focal participant's choices.

A total of 150 participants played games against nature that measured asocial skills. The participants played in pairs although the focal participant's choice did not affect her partner's pay-offs, or vice versa. The game was thus asocial (Figure 1).

**Figure 2.**
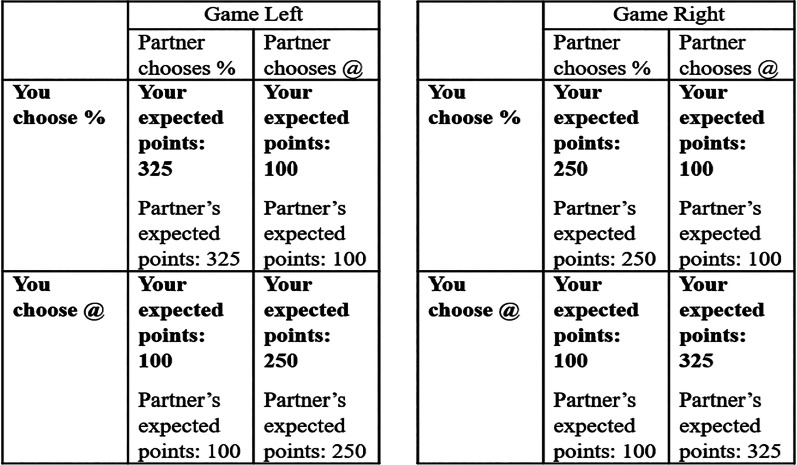
The payoff matrix shown to participants for the coordination game. Text in bold represents the expected payoffs from the focal participant's choices.

A total of 152 participants played coordination games measuring social norms (Figure 2). The participants earned more points if they chose the same option as an anonymous partner with whom they had been paired but could not communicate. Conditional on coordinating, one option had a high payoff. Like all coordination games, our games had multiple equilibria (Kets et al., 2021), and thus they presented players with an equilibrium selection problem. Through repeated play, participants could develop a norm regarding which equilibrium they would select. When such a norm emerges, coordination becomes straightforward because of a shared understanding of how to play.

During both economic games, the participants chose between two arbitrary options: @ or %. To allow the option with the highest possible payoff to change between blocks, both the game against nature and the coordination game came in two versions: ‘Game Left’ (% optimal) and ‘Game Right’ (@ optimal). We used these labels to avoid obvious focal points. For example, labels ‘A’ or ‘B’ might have allowed participants to coordinate by simply choosing ‘A’. Arbitrary symbols prevented this.

The points reflected in Figures 1 and 2 represent the expected points for the participants’ choice, although points were influenced by a random disturbance drawn from a normal distribution of mean (*M*) = 0, SD = 20 points (Molleman & Gächter, 2018). This disturbance was drawn independently for each participant in each period and occasionally meant that choosing the ‘suboptimal’ option would give higher points than choosing the ‘optimal’ option (McElreath et al., 2005).

### 2.3. Materials

The participants read an instructional booklet specific to the game being played in their session (see Appendix 2). The participants played anonymously at individual PCs. The games were ran via Z-Tree version 3.5 (Fischbacher, 2007; Appendix 3). All participants answered a demographics survey at the end and social learners stated their social learning preferences (see Supplementary Material S1).

### 2.4. Procedure and design

Participants were tested in groups of 20–30. To start the session, six participants were randomly assigned to play as demonstrators (labelled Type A in game). The remaining participants were social learners (labelled Type B in game). The numbers were even in each session so that the game could be played in pairs. Pairs were constrained so that demonstrators only played with other demonstrators and social learners only played with other social learners. To begin, the participants answered questions confirming their understanding of the game's instructions.

The games in each session lasted for 22 blocks of four periods. The pair for each block remained the same, although the pairs could randomly change between blocks. Each block started with the computer randomly assigning the demonstrators to play Game Left (% optimal) or Game Right (@ optimal). The optimal option could change between but not within blocks. The demonstrators played first in each block. They did not know which game they were playing but saw the points they had earned after each choice. They could thus learn which option was likely to be optimal across the four periods within the block. The noise applied to the points in Figures 1 and 2 was essential to ensure that the blocks ended with a mixture of demonstrators answering optimally and suboptimally. If performance was at ceiling, then there would be no variation for our social learners to respond to.

**Figure 3.**
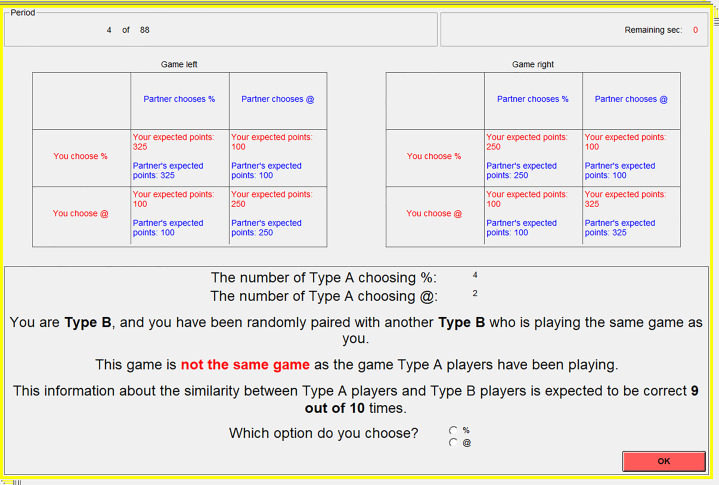
A typical round for the social learners. Type A participants were demonstrators and Type B participants were social learners. We avoided the term ‘demonstrator’ or ‘social learner’ in case it led the participants to respond in certain ways. The top half of the screen reminds the participants of the expected pay-offs from Game Left and Game Right (for the coordination game in this case). The bottom half of the screen contains frequency-dependent information (i.e. the number of demonstrators who chose @ or %), similarity information (i.e. whether the demonstrators were identified as playing the same or different game version to the social learners) and the reliability information.

The social learners chose in the final period of the block and saw no feedback. They could only base their decision on social information, including (a) the frequency of demonstrators who chose @ or % in the final period of the block (first-order social information), (b) a signal informing them that they played the same or different game version to the demonstrators (second-order social information) and (c) the reliability of the similarity signal (third-order social information). Figure 3 depicts a typical round for social learners.

The frequency information displayed the actual in-game choices of the demonstrators for the final period of that block. We manipulated both the similarity information (same or different) and the reliability information (reliably correct, uninformative, and reliably incorrect) in a 2 × 3 within-subjects design. The similarity information concerned the game being played. A ‘same’ signal implied that the social learners and demonstrators played the same game version (e.g. both played Game Left) and a ‘different’ signal implied the social learners played the other game (e.g. the demonstrators played Game Left, but the social learners played Game Right).

The reliability information was conveyed as the probability that this similarity signal was correct: 1 in 10 times for a reliably incorrect signal; 5 in 10 times for an uninformative signal; and 9 in 10 times for a reliably correct signal. To assign these, the computer started each block by randomly assigning the social learners into three similarly sized groups: ‘reliably correct’, ‘uninformative’ and ‘reliably incorrect’. The computer then tracked whether the game type assigned to the social learners matched that assigned to the demonstrators. The actual games being played, plus the reliability group to which the social learner was assigned, determined the similarity signal so that the probabilities were not deceptive. For example, if the social learner and the demonstrator were both assigned to play Game Left, and the social learner was in the ‘reliably correct’ group, then the social learner would see that they played the ‘same’ game version as the demonstrators with a probability of 0.9, and a signal indicating that they played a ‘different’ game to the demonstrators with the remaining probability of 0.1.

Finally, the participants answered the survey and were paid separately based on the points they earned (₹14 per point for the game against nature, ₹23 per point for the coordination game). Including a ₹100 show-up fee, the participants made an average of ₹958.53 (SD = ₹24.34) during the game against nature and ₹827.23 (SD = ₹86.81) during the coordination game. This was equivalent to £9.60 (SD = 24p) (game against nature) and £8.95 (SD = 56p) (coordination game) based on the current exchange rate. This study was self-certified via the Royal Holloway University of London's School of Psychology ethical criteria (see Appendix 4). The authors assert that all procedures contributing to this work comply with the ethical standards of the relevant national and institutional committees on human experimentation and with the Helsinki Declaration of 1975, as revised in 2008.

### 2.5. Analysis

The analysis had three steps. First, we confirmed that the demonstrators provided varied – but usually accurate – social information (see Section 3.1). Second, we investigated how flexible the social learners were when adjusting their choices to the social information (see Section 3.2). Third, we investigated how efficient the social learners were at extracting optimal social information from the different groups from whom they learned (see Section 3.3; see Appendix 5 for scripts).

The second analysis investigated the flexibility in the social learners’ frequency-dependent social learning strategies by predicting whether they chose % based on the social information. Our predictors were: (a) the centred proportion of demonstrators who chose %; (b) dummies for each combination of the similarity-reliability information; and (c) interactions between these dummies and the centred proportion of demonstrators who chose %. The dummies were: reliably incorrect–different signals; uninformative–similar signals; uninformative–different signals; reliably correct–similar signals; and reliably correct–different signals. The omitted category was reliably incorrect–similar signals. We analysed these as dummies to avoid modelling three-way interactions, which can be difficult to interpret in large analyses.

The social learners may be flexible in their choices but still experience trade-offs if they find some social information easier to process than others. To investigate this, our third analysis predicted whether the social learners chose the social learner optimum based on: (a) the centred proportion of demonstrators who chose the demonstrator optimum; (b) the similarity and reliability dummies as above; and (c) the interaction between these dummies and the centred proportion of demonstrators who chose the demonstrator optimum.

Demographics such as age (Wen et al., 2019) and gender (Mesoudi et al., 2015) can influence conformity preferences. All analyses were repeated again, with certain demographics entered as control predictors (see Appendices 7 and 9). Although we report the full data in-text, one session of participants did not complete the demographics owing to a crash during the coordination game only. Thus, the coordination game analysis with control predictors in Appendices 7 and 9 only gives the data for *N* = 278 participants, as we excluded the crashed session. For transparency, we repeat all analyses with this crashed session removed for the coordination game in Supplementary Material S2. The methods, *a-priori* sampling plan and power size were preregistered. However, we did not preregister a specific analysis plan as we wished to remain explorative owing to the novelty of testing social information use to a third-order complexity.

### 2.6. Predicted social learning strategies

The uninformative signal rendered the similarity information at chance level of being correct and so we only predict social learning strategies for the informationally equivalent trials (i.e. reliably correct and reliably incorrect signals of similarity and difference). As this study was novel in testing three orders of social information, we do not make exact hypotheses of how we expect the social learners to respond. We do note, however, the social learning strategies that would allow the social learner to choose the social learner optimum (assuming that the demonstrators chose the demonstrator optimum). When the social learners played the same game as the demonstrators, then the social learner and demonstrator optimum were likely to align. The social learners could copy the majority of demonstrators if they wished to answer optimally. Both a reliably correct signal of similarity and a reliably incorrect signal of difference implied that the social learner and demonstrator optimum matched, and so the social learners could follow the majority under both these trials (see Table 1). Conversely, a reliably correct signal of difference and a reliably incorrect signal of similarity implied that the demonstrator optimum was different from the social learner optimum. The social learners would thus copy the minority of demonstrators under both these blocks to choose the social learner optimum (see Table 1).

**Table 1.** The strategies that allow the social learners to extract optimal pay-offs when seeing one of the informationally meaningful trials.

		Third-order social information	
		Reliably incorrect	Reliably correct
**Second-order social information**	Similar	Follow the minority	Follow the majority
	Different	Follow the majority	Follow the minority

## 3. Results

### 3.1. Did the demonstrators provide varied yet accurate social information?

As the demonstrators’ choices formed the frequency-dependent social information shown to the social learners, then we must confirm that the demonstrators chose the demonstrator optimum by the final period of the block. This is similar to how individuals master skills and norms via their own trial-and-error. The demonstrators must show more than a trivial preference for the social learners to be able to use social information optimally (Efferson et al., 2016).

Table 2 shows that the demonstrators were more likely to answer optimally on the final period than any other period within a block (‘finalPeriodDummy’: game against nature estimate = 0.378, *p* < 0.001; coordination game estimate = 0.325, *p* = 0.0004), showing trial-and-error learning. We also include the ‘% as optimal’ predictor in Table 2 to see whether the demonstrators showed a trivial preference for one symbol over the other. No such bias existed for the game against nature (‘% as optimal’: estimate = 0.178, *p* = 0.54), but the significant beta for the coordination game suggested a bias for the demonstrators to choose % (‘% as optimal’: estimate = 1.146, *p* < 0.001). This preference to choose % arbitrarily may have helped the demonstrators to coordinate during the coordination game, regardless of the payoffs for this choice.

**Table 2.** A logistic regression modelling whether the demonstrators chose their demonstrator optimum. It includes a dummy for the final period of the blocks and % as optimal as predictors. Model 1 displays the data for the game against nature (asocial skills) and Model 2 displays the data for the coordination game (social norms). Robust standard errors in parentheses are clustered on the demonstrator. We also include the lower and upper limit 95% confidence interval for each estimate below this standard error.

Parameter	Estimate (game against nature)	Estimate (coordination game)
**Intercept**	0.321 * (0.161) 95% CI [0.01, 0.64]	−0.130 (0.154) 95% CI [−0.43, 0.17]
**Final period dummy**	0.378 *** (0.091) 95% CI [0.20, 0.56]	0.325 *** (0.092) 95% CI [0.15, 0.51]
**% as optimal**	0.178 (0.290) 95% CI [−0.39, 0.75]	1.146 *** (0.260) 95% CI [0.64, 1.66]

Asterisks denote the level of significance of our *p*-values, with the following key: *** *p* < 0.001; ** *p* < 0.01; * *p* < 0.05; trend, *p* = 0.05–0.10 significance.

To further investigate how this arbitrary preference may influence the demonstrators’ choices, we calculated the number of demonstrators who answered optimally by the final period of the average block (see Appendix 6). Some 68.64% of demonstrators answered optimally by the average final period for a game against nature, and 68.11% for the coordination game. One-sample *t*-tests confirmed that these percentages were significantly more than the 50% of demonstrators who would have answered optimally by chance (game against nature: (*t*(21) = −2619.9, *p* < 0.001); coordination game: *t*(21) = −2223.70, *p* < 0.001).The demonstrators thus used trial-and-error to answer better than chance. This means that the social learners could use the social information to help them answer optimally on approximately two-thirds of the blocks, provided that the signal was informationally meaningful (i.e. reliably correct or reliably incorrect). The demonstrators thus provided varied but typically accurate social information.

### 3.2. Did the social learners flexibly adjust their strategies to each level of the social information presented?

**Figure 4.**
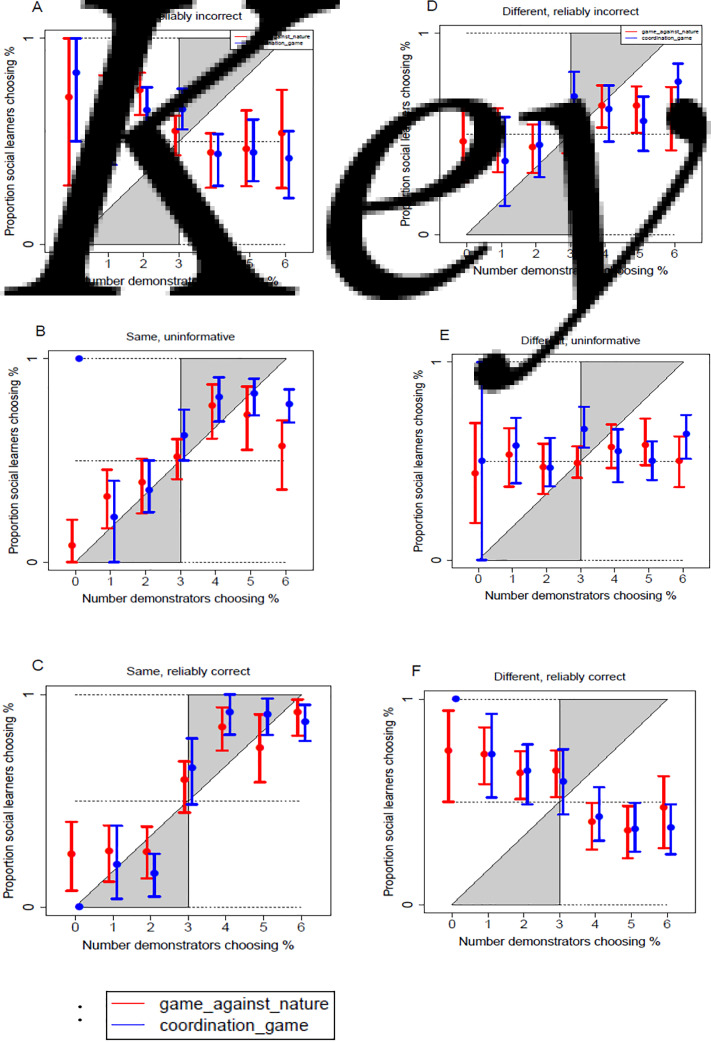
The proportion of social learners choosing % based on the number of demonstrators choosing %. The panels show the social learners’ frequency-dependent social learning strategies for each level of the second- and third-order social information, for both the game against nature (learning skills, in red) and the coordination game (learning social norms, in blue). The error bars give the 95% bootstrapped confidence interval clustered on social learners, to reflect the multiple observations gathered per learner. The regions shaded in grey depict where the social learners’ data would fall if they conformed, whilst the dashed lines give points of reference for proportions of learners choosing % at 0, 0.5, and 1.

To visualise the social learners’ chosen frequency-dependent social learning strategies in response to each level of the similarity and reliability information, Figure 4 plots the proportion of social learners who chose % as a function of the number of demonstrators who chose %.

For clarity going forward, we refer to blocks where the social learners were told that they played the ‘same game’ as demonstrators as learning from ‘similar others’, and blocks where the social learners played a ‘different game’ to the demonstrators as learning from ‘different others’. The social learners typically changed their preference for choosing % as based on whether the majority or the minority of demonstrators had chosen this in Figure 4, showing that they did respond to first-order social information. This was not equivalent for all signals, however. The response to frequency was less pronounced when viewing a reliably incorrect–different signal (Figure 4D), and seemingly random in response to an uninformative–different signal (Figure 4E).

To understand how the social learners responded to second-order social information, one need only compare the side-by-side panels of Figure 4, e.g. reliably correct signals in the bottom row. The social learners followed the majority of reliably correct–similar others (Figure 4C), although the followed the minority choice made by reliably correct–different others (Figure 4F). These matched the strategies predicted in Table 1. The social learners responded to second-order information, in a way which suggested that they were trying to extract their social learner optimum. To understand the social learners’ responses to third-order social information, one need only compare the panels in the same column of Figure 4, e.g. similar others in the left-hand column. The social learners followed the majority when learning from those with uninformative–similar (Figure 4B) and reliably correct–similar signals (Figure 4C), although they followed the minority when learning from those with reliably incorrect–similar signals (Figure 4A). This again matched the strategies depicted in Table 1. The social learners responded to third-order social information in a way that suggested that they tried to extract the social learner optimum. The logistic regression in Table 3 further investigates how the social learners adjusted their strategies to each level of social information.

**Table 3.** The logistic regressions modelling whether social learners chose %. Predictors included (a) the centred number of demonstrators who chose % in their final period, (b) each combination of the similarity and reliability information, minus the omitted category of reliably incorrect–similar signals and (c) the interactions between each of these dummies and the centred proportion of demonstrators who chose %. We centred the proportion so that any block where 3/6 demonstrators chose % became the omitted category of the regression and thus were reflected in the intercept. The robust standard errors given in parentheses were clustered on the social learner to reflect the multiple observations gathered per learner. See Appendix 7 for the regressions with control predictors. As the only significant control predictor was an increased likelihood to choose % as the blocks progressed during the game against nature only, the models reported below just focus on the social information of interest. We also give the 95% confidence interval lower and upper bounds for each estimate.

Parameter	Estimate (game against nature)	Estimate (coordination game)
**Intercept**	0.337 ** (0.12) 95% CI [0.10, 0.57]	0.308 * (0.120) 95% CI [0.07, 0.54]
**Centred proportion of demonstrators choosing %**	−1.493 * (0.581) 95% CI [−2.63, −0.36]	−1.400 ** (0.492) 95% CI [−2.36, −0.44]
**Reliably incorrect**–**different dummy** **[signal indicates different and is correct with 0.1 probability]**	−0.177 (0.145) 95% CI [−0.46, −0.11]	−0.112 (0.175) 95% CI [−0.45, 0.23]
**Uninformative–same dummy** **[signal indicates same and is correct with 0.5 probability]**	−0.251 (0.143) 95% CI [−0.53, −0.03]	−0.007 (0.154) 95% CI [−0.31, 0.29]
**Uninformative–different dummy** **[signal indicates different and is correct with 0.5 probability]**	−0.274. (0.158) 95% CI [−0.58, −0.03]	−0.065 (0.154) 95% CI [−0.37, 0.24]
**Reliably correct–same dummy** **[signal indicates same and is correct with 0.9 probability]**	0.006 (0.152) 95% CI [−0.29, 0.30]	−0.089 (0.197) 95% CI [−0.47, −0.30]
**Reliably correct–different dummy** **[signal indicates different and is correct with 0.9 probability]**	−0.034 (0.137) 95% CI [−0.30, 0.14]	−0.025 (0.168) 95% CI [−0.35, 0.30]
**Centred proportion of demonstrators choosing %** × **reliably in**correct–different **dummy**	2.371 ** (0.750) 95% CI [0.90, 3.84]	2.911 *** (0.751) 95% CI [1.44, 4.38]
**Centred proportion of demonstrators choosing %** × **uninformative**–**same dummy**	4.161 *** (0.802) 95% CI [2.59, 5.73]	4.242 *** (0.752) 95% CI [2.77, 5.71]
**Centred proportion of demonstrators choosing %** × **uninformative**–**different dummy**	1.785 * (0.695) 95% CI [0.43, 3.14]	1.630 * (0.677) 95% CI [0.31, 2.95]
**Centred proportion of demonstrators choosing %** × **reliably correct**–**same dummy**	5.714 *** (0.992) 95% CI [3.77, 7.65]	6.700 *** (0.989) 95% CI [4.77, 8.63]
**Centred proportion of demonstrators choosing %** × **reliably correct**–**different dummy**	−0.438 (0.640) 95% CI [−1.69, −0.81]	−0.576 (0.642) 95% CI [−1.83, 0.68]

Asterisks denote the level of significance of our *p*-values, with the following key: *** *p* < 0.001; ** *p* < 0.01; * *p* < 0.05; trend, *p* = 0.05–0.10 significance.

Table 3 depicts a remarkable consistency between how the social learners played the game against nature and the coordination game. In both games, the social learners were less likely to choose % as more demonstrators chose this (game against nature, effect = −1.49, *p* = 0.01; coordination game, effect = −1.40, p = 0.004). This reflects the estimate for the omitted category of reliably incorrect–similar signals. The social learners seemingly understood that groups who were unlikely to be playing the same game as themselves were in fact likely to play a different game, and so they should follow the minority choice. The social learners also followed the minority in response to reliably correct–different signals (Figure 4F), suggesting that they understood these signals were informationally equivalent.

The significant betas in Table 3 reveal that the social learners had a significantly distinct social learning strategy from the reliably incorrect–similar dummy under the following blocks: reliably incorrect–different; uninformative–similar; uninformative–different; and reliably correct–similar. Note that this beta merely confirms a significant difference. The random response to uninformative–different others (Figure 4E) was not meaningful, although it was significantly different to the social learners’ minority-based social learning strategy on the omitted block of reliably incorrect similar signals (Figure 4A). Interestingly, the social learners followed the majority in response to both reliably correct–similar (Figure 4C) and reliably incorrect–different blocks (Figure 4D), although this was less pronounced on the latter. Broadly speaking, the social learners understood that someone who was unlikely to play a different game to themselves was in fact likely to play the same game. The social learners showed a complex processing of third-order information and broadly understood which signals were informationally equivalent.

To further test the extent of social learners’ flexibility, we perform linear combinations between the social learners’ strategies in response to each block where either the majority of demonstrators (>4) chose %, or none of them had. The logic is this: flexible social learners would adjust to the similarity and reliability information, whilst those that merely followed a simple rule over first-order social information space (i.e. to always follow the majority, or minority) would simply respond differently to blocks where 0 of the demonstrators chose % compared with blocks where the majority had chosen %. This analysis revealed that the social learners adjusted their strategies distinctly to both similarity and reliability information, regardless of how many demonstrators chose %. The only exception was that there was no difference in social learners’ strategies between reliably incorrect–different (Figure 4D) and uninformative–different (Figure 4E) blocks for the game against nature (see Appendix 8).

To summarise, the social learners adjusted somewhat to each level of the social information. Section 3.3 focuses on whether these adjustments are complete and symmetric to all informationally equivalent blocks, by investigating whether the social learners chose their social learner optimum.

### 3.3. Did the social learners choose their social learner optimum?

If the social learners adjusted symmetrically to the social information available, then they should be able to respond optimally to any informationally equivalent block when playing both the game against nature and the coordination game (see Table 4). The social learners had no way of responding optimally to the uninformative blocks beyond chance, as these rendered the similarity information at chance likelihood of being correct.

**Table 4.** Logistic regressions modelling whether the social learners chose the social learner optimum. Predictors included (a) the centred proportion of demonstrators who chose the demonstrator optimum, (b) dummies for each combination of similarity and reliability information, minus the omitted category of reliably incorrect–similar signals and (c) interactions between each of these dummies and the centred proportion of demonstrators who chose the demonstrator optimum. Robust standard error clustered on social learner. See Appendix 9 for the regressions with control predictors, although the only significant control predictor was that the social learners were more likely to answer optimally on blocks where the % symbol was optimal, suggesting an arbitrary preference to choose this symbol across both games. We also give the 95% confidence interval lower and upper bounds for each estimate.

Parameter	Estimate (game against nature, all signals)	Estimate (coordination game, all signals, full data)
**Intercept**	0.210 (0.148) 95% CI [−0.08, 0.50]	0.047 (0.109) 95% CI [−0.17, 0.26]
**Centred proportion of demonstrators choosing the demonstrator optimum**	0.166 (0.642) 95% CI [−1.09, 1.42]	0.613 (0.462) 95% CI [−0.29, 1.52]
**Reliably in**correct–different **dummy** **[indicates different and is correct with 0.1 probability]**	−0.136 (0.184) 95% CI [−0.50, 0.22]	0.184 (0.165) 95% CI [−0.14, 0.51]
**Uninformative**–same **dummy** **[indicates same and is correct with 0.5 probability]**	−0.193 (0.201) 95% CI [−0.59, 0.20]	−0.026 (0.159) 95% CI [−0.34, 0.29]
**Uninformative**–different **dummy** **[indicates different and is correct with 0.5 probability]**	−0.131 (0.193) 95% CI [−0.51, −0.25]	−0.136 (0.160) 95% CI [−0.45, 0.18]
**Reliably correct**–same **dummy** **[indicates same and is correct with 0.9 probability]**	−0.278 (0.222) 95% CI [−0.71, −0.16]	0.254 (0.204) 95% CI [−0.14, 0.65]
**Reliably** correct–different **dummy** **[indicates different and is correct with 0.9 probability]**	−0.048 (0.192) 95% CI [−0.42, −0.33]	−0.017 (0.168) 95% CI [−0.35, 0.31]
**Centred proportion of demonstrators choosing optimum** × **reliably in**correct–different **dummy**	0.374 (0.815) 95% CI [−1.22, 1.97]	0.786 (0.622) 95% CI [−0.43, 2.00]
**Centred proportion of demonstrators choosing optimum** × **uninformative**–same **dummy**	−0.787 (0.819) 95% CI [−2.39, 0.81]	−1.197 * (0.595) 95% CI [−2.36, −0.03]
**Centred proportion of demonstrators choosing optimum** × uninformative–different **dummy**	−0.901 (0.805) 95% CI [−2.48, 0.67]	−0.023 (0.616) 95% CI [−1.23, 1.18]
**Centred proportion of demonstrators choosing optimum** × **reliably correct**–same **dummy**	2.53 ** (0.911) 95% CI [0.75, 4.31]	1.460. (0.770) 95% CI [−0.05, 2.96]
**Centred proportion of demonstrators choosing optimum** × **reliably** correct–different **dummy**	0.724 (0.785) 95% CI [−0.81, 2.26]	0.573 (0.605) 95% CI [−0.61, 1.76]

Asterisks denote the level of significance of our *p*-values, with the following key: *** *p* < 0.001; ** *p* < 0.01; * *p* < 0.05; trend, *p* = 0.05–0.10 significance.

In the game against nature, the social learners were significantly more likely to choose their social learner optimum as more demonstrators chose the demonstrator optimum for a reliably correct- similar dummy (effect = 2.53, *p* = 0.005). The social learners conformed to the majority of groups with reliably correct–similar signals (Figure 4C), which allowed them to choose the social learner optimum on this trial. We further confirmed this preference to learn from reliably correct–similar others with linear combinations (see Appendix 10). The social learners were more likely to respond optimally when viewing reliably correct–similar signals than uninformative–similar signals, although this was expected as the social learners could only respond optimally to the uninformative signals by chance. Interestingly, the social learners were also more likely to answer optimally when viewing reliably correct–similar signals than when viewing both reliably incorrect–similar signals, or reliably correct–different signals. This suggests a bias in processing information, as these signals were informationally equivalent. This implies a trade-off in the depth of social information that the learners processed, as they were more likely to master an asocial skill when learning from similar others with reliably correct signals.

For the coordination game, the social learners were less likely to choose their social learner optimum as more demonstrators chose their demonstrator optimum on uninformative–similar blocks (effect = −1.20, *z* = −2.01, *p* = 0.04). The uninformative signals provided a baseline trial in which the social learners could choose the strategy they preferred. Despite this, the social learners seemingly employed a strategy of ‘copy similar others even if the blocks give uninformative information’ (Figure 4B). This perhaps helped the social learners to coordinate, although they were more likely to coordinate on the suboptimal option. Linear combinations (Appendix 11) again confirmed that the social learners were more likely to answer optimally when responding to reliably correct–similar others than uninformative–similar, reliably incorrect–similar and reliably correct–different others during the coordination game. The bias to copy reliably similar others was perhaps less pronounced on a coordination game than a game against nature, although.

A caveat of these results is that the Z-Tree code worked with the probabilities given in the economic games (0.1 for reliably incorrect, 0.5 for uninformative and 0.9 for reliably correct signals). Thus, the similarity signal was not always accurate. For example, if the social learner was assigned to the reliably incorrect group and was told that she played the same game version as the demonstrators (i.e. reliably incorrect–similar signal). This signal could correctly depict that the social learner and the demonstrators played the same game with a probability of 0.1 but was more likely to provide incorrect information with a probability of 0.9. The fact that the similarity signal could be incorrect could have affected the social learners’ ability to choose their social learner optimum.

We thus analysed how the social learners responded to similarity signals that happened to be correct vs. those that happened to be incorrect, for both the game against nature (see Appendix 12) and the coordination game (see Appendix 13). Broadly speaking, these analyses revealed that the social learners responded to reliably correct signals as if they always provided correct information and responded to reliably incorrect signals as if they always provided false information. This shows a complex processing of reliability information. The social learners also treated an uninformative signal of similarity as if these were always correct during the coordination game, perhaps as this strategy facilitated coordination.

To summarise, the social learners may have occasionally coordinated on the suboptimal option during the coordination game, but they were usually flexible at adjusting to social information when acquiring a social norm. There was remarkable consistency between how the learners acquired both an asocial skill and a social norm, with a preference to learn from reliably correct–similar others in both games.

### 3.4. Summary of the social learning strategies

The social learners adjusted their frequency-dependent social learning strategies to (a) frequency information, (b) signals cuing whether the group played a similar or different game to the participant and (c) the reliability of similarity signals. Frequency-dependent social learning strategies were flexible to three orders of social information but with a trade-off. The social learners were more likely to respond optimally when learning from reliably correct–similar others, for both an asocial skill and a social norm.

## 4. Discussion

This study found that social learners used frequency-dependent social learning strategies that were functions of first-order information (choice frequencies), second-order information (signal of similarity) and third-order information (reliability of similarity signals). Frequency-dependent strategy spaces are more extensive than one-dimensional responses to choice frequencies. These results corroborate a growing body of literature suggesting that social learning strategies are contingent, flexible and facultative (Deffner et al., 2020; Efferson et al., 2016; Kendal et al., 2018; Rendell et al., 2011). More technically, social learning strategies should be thought of as functions defined over relatively high-dimensional domains. This kind of high dimensionality is important because it implies that the trade-offs typically assumed to hold in gene–culture models of social learning will not necessarily hold (Efferson et al., 2016).

In our experiment, social learners responded differently to choice frequencies in a group of (reliably) similar others vs. a group of (reliably) different others. Perhaps a history of intergroup contact (Boyd & Richerson, 1985: chapter 7; Deffner et al., 2020; Efferson et al., 2008b) exposed ancestral humans to different others often enough to select for the processing of second-order social information. Being able to adjust strategies to second-order social information may attenuate the trade-off associated to conformity use around different others (Efferson et al., 2016).

We implemented second-order information by telling participants whether they were making decisions in an environment similar to or different from the demonstrators being observed. Previous research, in contrast, has focused on whether participants *look* similar (House et al., 2013; Jiménez & Mesoudi, 2019; Molleman et al., 2019a; Salali et al., 2015; Shutts et al., 2010). Similarity in appearance may sometimes be a useful way to identify those who need the same skills or share the same social norms as one's self (Efferson et al., 2008b; Richerson et al., 2016; Fischer, 2009; Wood et al., 2013). In our case, however, we explicitly conveyed similarity to avoid participants having their own view of what similarity of appearance might mean, which can be hard to measure.

Future work could nonetheless explore frequency-dependent social learning strategies based on apparent similarity to explore ethnocentrism (Hales & Edmonds, 2019) and out-group prejudices (Efferson et al., 2008b; Konrad & Morath, 2012; Vogt et al., 2013). Participants could have on-screen avatars, for example, where similarities among avatars could reliably reveal similar environments (cf. reliably correct). On other trials, similarity amongst avatars may suggest that the demonstrators play the opposite game type to that expected, (cf. reliably incorrect) or the avatars may fail to become meaningfully linked to the decision-making environment (cf. uninformative).

Social learners also processed third-order social information, although they performed best when learning from a group of reliably similar others. This supports case (b) in the introduction. Participants can process three orders of social information, but adjustments were asymmetric. Social learners also copied the minority choice when responding to reliably incorrect signals of similarity. The ability to process reliably incorrect signals may be necessary as ethnic markers can be faked to exploit another group (Sosis et al., 2007), or can be signalled subtly or hidden entirely (Smaldino et al., 2018). The social learners clearly adjusted to the reliability information given alongside groups of similar others, although they did not adjust as definitively to the reliability signal given alongside different others. This is evidence of an asymmetric adjustment, as reliability was seemingly up-weighted when responding to groups of similar others.

This asymmetric adjustment may make sense. Outsiders would pretend to be similar to others to exploit another group's resources (Sosis et al., 2007), although it is hard to envision a case where an individual would pretend to be different to others. After all, failing to coordinate one's actions to the rest of the group may have devastating social consequences (Chudek & Henrich, 2011; Molleman et al., 2019b). Of course, there are cases where people may look different and still share similar optima, as is the case with a migrant to a new social group. This makes the inclusion of the reliably incorrect signal of difference an interesting test of cognition. A fully flexible domain-general processing system should be able to respond to all social signals (Bolhuis et al., 2011; Shenhav & Greene, 2010). Our social learners did not respond to reliably incorrect signals from different others, which instead suggests that social learner cognition is certainly flexible, but not endlessly so.

The origins of this asymmetric adjustment may be genetic or cultural. The bias to copy reliably similar others in the current study may be due to an evolved in-group preference, as we were more likely to encounter and learn from reliably similar in-group members in the ancestral past (Henrich & Boyd, 1998; Mercier & Morin, 2019; Molleman et al., 2014). Alternatively, individuals may socially learn how to learn from others (Kendal et al., 2018; Heyes, 2016; Mesoudi et al., 2016). Social learners may have responded to both reliably correct and reliably incorrect signals of similarity in the current study as they may have encountered both cases enough times in their own lives to have learned to respond to these signals. It is worth noting that this study cannot distinguish whether the asymmetric adjustment to learn from reliably similar others is genetic or cultural in nature, although future work should explore this. Instead, our novel contribution was to show that social learners can adjust their frequency-dependent social learning strategies at least up to three orders of social information.

Regardless of the origin, social learners made more money when responding under reliably correct–similar signals than under reliably incorrect–similar or reliably incorrect–different signals. An outstanding question remains as to whether all participants processed third-order social information and experienced the asymmetric adjustment, or whether some participants processed all three orders of social information flexibly whilst others merely followed a rule to ‘follow the majority’ or ‘the minority’. In the latter case, there would be variation in individual strategies that produce an average adjustment that is asymmetric at the aggregate level. To investigate this caveat, we build scatterplots and heatmaps which visually depict the range of social learning strategies at the individual level (see Appendices 14 and 15).

These graphs reveal two important findings. First, most participants lie somewhere different in the social learning strategy space when responding to reliably correct and reliably incorrect signals, showing that all participants attempt to adjust to three orders of social information. Second, while most participants attempted to adjust to three orders of social information, few did so optimally. Take similar others as an example. The optimal response to reliably correct signals would be to always copy the majority, and for reliably incorrect signals to always follow the minority. We found that some social learners did this. We also found that some social learners did the exact opposite of this, while some social learners were between these two extremes. Interestingly, the individuals who partially adjusted were not necessarily the same individuals who reported ignoring social information in the end survey (Supplementary Materials). Thus, asymmetric adjustment is not an artefact of averaging; it was a meaningful, if suboptimal, strategy.

Suboptimal responses to frequency-dependent information have appeared before in experiments without second- and third-order information. In two incentivised studies, Efferson et al. (2008a) find that a subset of participants will avoid conforming even when it would be suboptimal to do so, while Goeree and Yariv (2015) find that a subset of participants will conform even when conformity is suboptimal. Future research should investigate if this preference exists outside of laboratory studies, as people who are less responsive to frequency information and do not use it optimally are likely to have profound effects on the cultural evolutionary outcomes of information spread in their group.

Perhaps some social learners could adjust to the social information, and others only adjusted asymmetrically, owing to different cognitive strategies. Dual system approaches to social learner cognition suggest that learners can follow both simple learning rules and show complex adjustments (Heyes, 2016). System 1 processing is fast and effortless but driven by rules-of-thumb. This is consistent with how conformity has previously been modelled over one order of social information (Boyd and Richerson, 1985: chapter 7; Henrich, 2004; Henrich & Boyd, 2001). System 1 processing may explain the conformity preference around reliably similar others in our study. System 2 processing is slow and effortful, flexibly guiding who and when to copy (Efferson et al., 2016; Kendal et al., 2018; Rendell et al., 2011; Wood et al., 2013). Those capable of engaging System 2 cognition may have responded flexibly to all orders of social information in the current study, although not all of our participants adjust to third-order social information completely as System 2 thinking is difficult to engage. Indeed, a failure to update beyond simplistic System 1 biases in complex learning environments may explain why individually maladaptive behaviour, such as mob behaviour, can be upheld at the group level (Kendal et al., 2018; Mesoudi, 2009; Richerson & Boyd, 2005: Chapter 5).

Thus far, we have discussed the asymmetric adjustment to the three orders of social information to represent social learner cognition, although this could apply to human cognition more broadly. We suggested that the lack of response to reliably incorrect–different signals may be due to genetic or cultural evolutionary trade-offs, although this signal may also be difficult to respond to as processing double negatives is cognitively demanding (Cutmore et al., 2015; Johnson-Laird & Tridgell, 1972). The participants know to copy the majority of reliably similar others as they are likely to share optima. They must flip this logic to copy the minority of reliably correct–different others. The participants must then flip this logic again, to understand that they should follow the minority of reliably incorrect–different others as they are likely to have the same optima as themselves. Social learning may be just one task required of a cognitive system that cannot process information endlessly (Heyes, 2016; Krafft et al., 2021; Mesoudi, 2011).

For this paper, we treat a reliably correct and a reliably incorrect signal as informationally equivalent. Strictly speaking, this would only be true on tasks like our economic games where the participants choose between two options. Following the minority becomes less efficient on tasks with three or more options, as it can only rule out one strategy at a time. To illustrate with an example, imagine a hunter who wishes to know whether she should use a spear, a club or a net to hunt the local game. She observes fishermen using the net. This rules out the net as clearly that is designed for fishing. However, she is still none the wiser as to whether the club or the spear should be used to hunt. The use of two choice options is a clear limitation of our design, but it also allowed us to create four settings that packaged social information in four different but informationally equivalent ways.

Our results suggest that we should extend our view of frequency-dependent social learning strategy spaces to incorporate, somehow, at least three orders of social information. Future research may wish to model the gene–culture coevolution of such complex strategies to examine theoretical plausibility (Muthukrishna & Henrich, 2019). Future research should also move beyond frequency dependence to see whether individuals also process similarity and reliability information when learning from prestigious (Chudek et al., 2012) or socially dominant others (Flynn & Whiten, 2012).

In summary, social learners flexibly adjusted their frequency-dependent social learning strategies to (a) choice frequencies among demonstrators, (b) whether demonstrators were identified as learning in a similar or different environment and (c) the reliability of this similarity information. Taken together, these results suggest that definitions of frequency-dependent social learning strategies should be expanded to consider three orders of social information, as observed strategies were at least this complex. Social learners processed this information asymmetrically. They were more likely to master asocial skills and social norms when learning from reliably similar others. These complex strategies imply that the typical trade-offs of conformity in early gene–culture models may not hold. Instead, any trade-offs in the use of frequency-dependent social learning strategies are likely to be more nuanced than previously believed.

## Data Availability

The data that support the findings of this study are openly available in ‘What is a social learning strategy, anyway?’ at http://osf.io/t5d8h, reference number [DOI 10.17605/OSF.IO/T5D8H]. Data file is titled, ‘*coordGame_GameAgainstNat.RData’*.

## Research transparency and Reproducibility

The data collected for this study, alongside full materials, Appendices and Supplementary Materials, can be found at OSF | What is a social-learning strategy, anyway?

## Supplementary material

For supplementary material accompanying this paper visit https://doi.org/10.1017/ehs.2022.11. click here to view supplementary material
