# Supplementary material for: What is the extent of a frequency-dependent social learning strategy space?
Source: Evol Hum Sci. 2022 Apr 13;4:e13. doi: 10.1017/ehs.2022.11 (PMC10426114; doi:10.1017/ehs.2022.11)
Supplement: Supplementary file 1 [file S2513843X22000111sup.zip › S2513843X22000111sup002.pdf]

Appendices for ‘Chapter 3: What is the extent of a frequency-dependent social learning strategy space?’

Appendix 1A: Rationale for testing in India.

There is a bias to recruit WEIRD participants which current research trends must move away from (Henrich et al., 2010). Previous researchers have begun investigating the differences in social learning style preferences between British and Chinese participants (Molleman & Gachter, 2018). British participants are highly individualistic whilst Chinese participants are from highly collectivist cultures. This means that social learning preferences have been tested at polarised ends of Hofstede’s (1980) individualism scale. This data may not represent countries who are less differentiated and more central on this scale, and so the current study decided to recruit in India to address this gap. India ranks 21st on Hofstede’s individualism scale with a score of 48 (see https://clearlycultural.com/geert-hofstede-cultural-dimensions/individualism/ for classifications).

Henrich, J., Heine, S., & Norenzayan, A. (2010). The weirdest people in the world? *Behavioral and Brain Sciences, 33*(2-3), 61-83. doi:10.1017/S0140525X0999152X.

Hofstede, G. (1980). Culture and organizations. *International Studies of Management & Organization*, *10*(4), 15-41. https://doi.org/10.1080/00208825.1980.11656300.

Appendix 1B: Protocol given to researchers in CESS lab to ensure standardised testing across research labs. Note that the italicised text represents additional instructions that were used for the coordination game only.

Instructions for experimenters (experimenter use only)

1. Accept only an even number of participants. If odd, randomly select one person who showed up to send home.

2. Run the welcome treatment (.ztt file) provided on enough PCs for the invited number of participants. Turn off the PC screens before participants arrive. The welcome treatment displays a screen saying "Welcome, press OK", but participants will not be able to see it initially because their screens are off.

3. Provide an instructions booklet and pen at every client PC depending on the game type played.

4. As participants enter, direct them to their seat and tell them to start reading their instructions booklet. Tell them that when they have finished reading, they should fill in the multiple-choice questions and consent form at the back of the booklet if they wish to take part. They should then raise their hand to inform the researcher when they have finished. Please ensure that all participants are over the age of eighteen years old, capable of giving fully-informed consent and can speak English.

5. When participants raise their hand, use the answer sheet provided overleaf to check their understanding on the multiple-choice questions. If they have **not** answered all questions correctly, then ask them to try again referring back to the instructions booklet. If they have answered correctly, turn on their PC screen. At this point, they will see that they need to press “OK.” With the clients table open in z-Tree, this OK press will be visible to the

experimenter. In this way, the experimenter can keep track of who has answered all comprehension questions correctly.

6.A protocol regarding verbal instructions for those with poorer English abilities is provided overleaf. These participants will still have to answer the multiple-choice questions to show understanding however, and if they cannot understand these then they should be removed from the session with their show-up fee.

7.When the experimenter has verified that everyone has answered all comprehension questions correct, which again can be seen in the clients table, read the “Summary for participants” aloud in front of the entire session of participants.

8.Run the appropriate *coordGameWithinSubjectsIndependentSignals*ztt* or *bestChoiceWithinSubjectsIndependentSignals*ztt* file, depending on the game type played. Encourage participants to enter their unique ID that they input on the first screen on to the top of their consent form. After this, do not talk to participants unless they have a question to ask. Use the instructions, debrief, or this protocol to help you answer any questions that a participant may have. If there are any questions that you cannot answer, please tell the participant to contact me using the email address that I have provided both in their instructions and debrief.

9.Once all participants have finished the game, call participants up to pay them privately according to your standard procedure. Remember to take their consent form. Please provide the debrief form to the participants with every payment.

<u>Protocol for verbal instructions</u>

Read the below aloud in cases where the participant is slow to read English. They will still need to look at the screen-shots and answer pre-game questions. If they still do not understand after the verbal summary, then they may have to be removed. Where possible try

to avoid testing participants who may be of poor English ability. It is preferable that participants read rather than relying on this verbal summary. If they have further questions, then use the protocol/instructions booklet to answer them. Do not provide them with information that is not in the booklet, or help them answer questions, as this may give them an unfair advantage. Note that text in *italics* need only be read for the coordination game specifically.

"You are about to play a game on the computer which involves choosing between two options (@ or %). The order of these options on-screen may change throughout the game, so pay attention when choosing. You will play the game for 88 periods, which are divided into 22 blocks of 4 periods each. Your exact number of turns depends on your participant type. The computer starts by randomly choosing six of you to be Type A Participants. The others are Type B Participants.

Type A Participants make a decision in every period and thus make 88 choices. Type B Participants decide in the last period of every block, making 22 choices overall. Don't worry- Type B Participants will have the chance to earn equal points (and therefore, money) to Type A Participants.

The game is played in pairs. Your pair will be the same type as you (meaning that As play with As, and Bs play with Bs). You will be assigned a new partner in each new block of the game. Your points earned will also depend on what your partner chooses (see the points table in the booklet).

The computer will randomly decide which game Type A Participants play, and which game Type B Participants play. The two games are Game Left and Game Right. *Regardless of game-type, you will earn more points if you match your choice to your partner's (e.g. both pick %).* Which option (% or @) it is worth the most points to match on depends on which

game you are playing. Type A and Type B Participants may be playing the same game, or different. You do not know which game you are playing.

Type A Participants choose every period and immediately see the points that they have earned based on their choice. Note that a random shock will be applied to these points, so you may earn more or less points than the expected points shown in the table on-screen. This shock represents the effect of things beyond your control that affect your decisions in every-day life.

Type B Participants only choose in the final period of each block. Before choosing, they will see (i) how many Type A Participants have chosen @ or % on their final (fourth) turn, (ii) a signal indicating whether they are playing the same game (Left or Right) as Type A Participants, and (iii) the probability that this signal is correct. If you are a Type B participant, you and your partner will always see the same information. The information may change, however, from one block to the next, so please pay attention to the on-screen information.

Type B Participants will not see the points that they have earned based on their choices, until they see their total points earned at the end of the game. Each of Type B's choices are played four times, with four separate random shocks, so that they earn similar points to Type A Participants.

After playing the game, you will complete a short survey about how you played the game. Fill in the multiple-choice answers by clicking the option that most applies to you with your mouse, and fill in text answers by clicking inside the purple box and typing your answer.

We ask that you remain seated at the end of the study and wait until you hear your ID number being called to accept your pay and debrief before you leave.

If you have any questions, or would like to leave for whatever reason, then please ask now. Remember not to talk to your fellow participants during the game. If you wish to leave during the game, or have any questions, then raise your hand to let a researcher know.

If you are happy to take part, then please now fill in your consent form and answer the multiple-choice questions in the back of your booklet. You must answer all questions correctly to start playing".

Predicted questions and how to answer (for experimenter use only)

• **What is the experiment actually about?**

This study will help us understand how people use information to make decisions, and the diversity of this process, particularly in regards to decisions made in groups.

• **What will the findings be used for?**

The findings will be used as part of a PhD project being ran at Royal Holloway University of London. The lead researcher's details are in your instructions booklet and on the debrief given to you at the end. This study may be published but we have taken care to ensure that your decisions are anonymous and will not be traced back to you individually.

• **I'm unsure what a Type A Participant does**

You will see some tables denoting game left or game right. The computer has chosen one of these games to play, though you do not know which. *You are playing the same game as your partner.* You should choose between @ or %, and hit OK when you're happy with your choice. You can then see the points that you have earned *based on both your decisions, and your partner's decisions,* after everyone has answered. One option will be worth more points than the other option, *provided that you and your partner choose the same option.* You have four turns at choosing in every block. Read the instructions again for further information.

•I'm unsure what a Type B Participant does

Type B Participant have one chance to match their choice of % or @ with their partner's in every block. You are playing Game left or Game right, and this may be the same game as Type A participant's or different. You will see some information on-screen to help you make your choice. This includes the number of Type A Participants choosing @ or %, and whether you are playing the same or different game to these participants. You will also be told how likely it is that this information about playing the same or different game is to be correct. Remember that the partner you are paired to play the game with always sees the same information as you. The on-screen information and your assigned partner can change between blocks however, so you should play close attention each time you make a decision. Read the instructions again for more information.

•How do I answer the survey?

Try to answer these questions as honestly as you can. Answer the multiple-choice questions by using your mouse to click the white button next to your desired answer. Please select the answer that you feel most applies to you. The final field requires text answers. Click inside the purple text box and manually type your responses. When you are done hit OK and then raise your hand to let the researcher know.

•You may have to remind people to type their age and years lived in their city as numbers instead of text (i.e. "22" instead of "twenty-two").

•If the participant sees an error message in a different language: this means that they have not clicked the option or typed their answer properly and must do so to proceed. Tell them to hit OK and answer again.

•If participants see an extra question screen at the end repeating the age questions, then the individual has entered their years lived in a country as more than their age by

mistake in the first survey. Tell them that they are being asked to rectify any small mistakes in a separate screen at the end.

- Any other questions, ask them to email me after the experiment using the address that I included on both the instructions and the debrief.

Appendix 2: The instructions given to the participants including pre-game questions to check understanding.

Appendix 2A: Instructions for the game against nature.

Welcome! You are invited to participate in a study for approximately **1 ½ hours.** You can earn points during this study, which will be converted to money at the following rate:

14 points = ₹1

You will also be paid a show-up fee of **₹100** on top of the money you earn. The choices participants make during the study will be anonymous. This means you will not be able to identify the specific participants in the room who make certain choices, and none of the participants will be able to trace your choices back to you.

Please do not communicate with the other participants. If you have questions, or need to withdraw, then please raise your hand and tell the researcher.

Please read this instruction sheet **carefully**. You will then answer some questions to check that you have understood the study. We will not be able to proceed until everyone answers **all** questions correctly. You will also respond to a brief **survey** after the main study.

The study:

To begin, the computer will randomly choose six of you to be Type A Participants. Others will be Type B Participants. As explained later, your type will determine how often you make choices and the information you have when you do so. The study lasts for 88 periods. We will divide these 88 periods into 22 blocks of 4 periods each. Type A Participants will choose every period, which means they will make 88 choices. Type B Participants will only make a choice in the final period of each block, which means that Type B Participants will make 22 choices. Don't worry. Though Type A and Type B Participants

do not make the same number of choices, they will have exactly the same opportunity to earn points. We will explain this in detail later. The upper left-hand corner of your screen will have a counter that displays the current period you are in.

The games:

At the beginning of each block of 4 periods, the computer will randomly pair you with another participant of the same type to play a game. A Type A Participant will always be paired with another Type A, and a Type B Participant will always be paired with another Type B Participant. Every time you play, both you and your partner must choose between one of two options, either option "%" or option "@".

Specifically, there are two games, which we call "Game Left" and "Game Right". At the beginning of each block, the computer will randomly pick which game Type A Participants play and which game Type B Participants play. The computer decides this completely randomly, giving four possible combinations, which are all equally likely to occur (each with a 1 in 4 probability). The four possibilities are:

(i) both types play Game Left,

(ii) Type A Participants play Game Left, Type B Participants play Game Right,

(iii) Type A Participants play Game Right, Type B Participants play Game Left,

(iv) both types play Game Right.

Note that we do not tell you if you are playing Game Left or Game Right. The following tables show you how your points will depend on the choices you make, for each of the games that you might play.

Game left

	Partner chooses %	Partner chooses @
You choose %	Your expected points: 150<br>Partner's expected points: 150	Your expected points: 150<br>Partner's expected points: 120
You choose @	Your expected points: 120<br>Partner's expected points: 150	Your expected points: 120<br>Partner's expected points: 120

Game right

	Partner chooses %	Partner chooses @
You choose %	Your expected points: 120<br>Partner's expected points: 120	Your expected points: 120<br>Partner's expected points: 150
You choose @	Your expected points: 150<br>Partner's expected points: 120	Your expected points: 150<br>Partner's expected points: 150

As you can see, one option is worth more points and will therefore result in you earning more money, on average, if you pick this option. The option (% or @) that is worth the most points is different depending on whether you are playing Game Left or Game Right.

You can also see that your payoff will **not** depend on what your partner chooses in any way. Nor will your partner's payoff depend on what you choose.

Lastly, points will also be affected by forces outside of your control, as in real life. The tables above shows the expected points you will earn, but random shocks will be applied to these values. These random shocks can lead you to earn **more OR less** points than the expected values shown in the tables.

For example, assume you are playing Game Left. It is possible that you could earn more by choosing @ than you could by choosing % for a single choice. It is more likely, however, that % will earn more than @, and so when choosing repeatedly % is highly likely to produce the most points.

Similarly, assume you are playing Game Right. It is possible that you could earn more by choosing % than by choosing @ for a single choice. It is more likely, however, that

@ will earn more than %, and so when choosing repeatedly @ is highly likely to produce the most points.

In summary, the points that you earn will depend on three things.

1. **The game being played:** Game Left or Game Right.
2. **The option you choose:** Option % or Option @.
3. **The random shock:** Random shocks are added to an expected payoff. Random shocks are independent of each other. This means that sometimes they will lead to more points than expected, and sometimes they lead to less points than expected.

IMPORTANT: The computer chooses the games being played at the beginning of each block. The games being played can change from one block to the next, and you will be paired with a different partner of the same type as you in each new block.

Type A vs. Type B Participants

As explained above, before the study begins, the computer will randomly select six participants to be Type A participants. Others will be Type B participants. Your type will not change.

Type A Participants -> Type A Participants choose every period, and each Type A Participant immediately sees the points he or she earns after making a choice.

IMPORTANT: The computer chooses the games being played at the beginning of each block of four periods. Note that the game being played does **NOT** change across the four periods within a block. Here is an example of a choice screen for a Type A Participant:

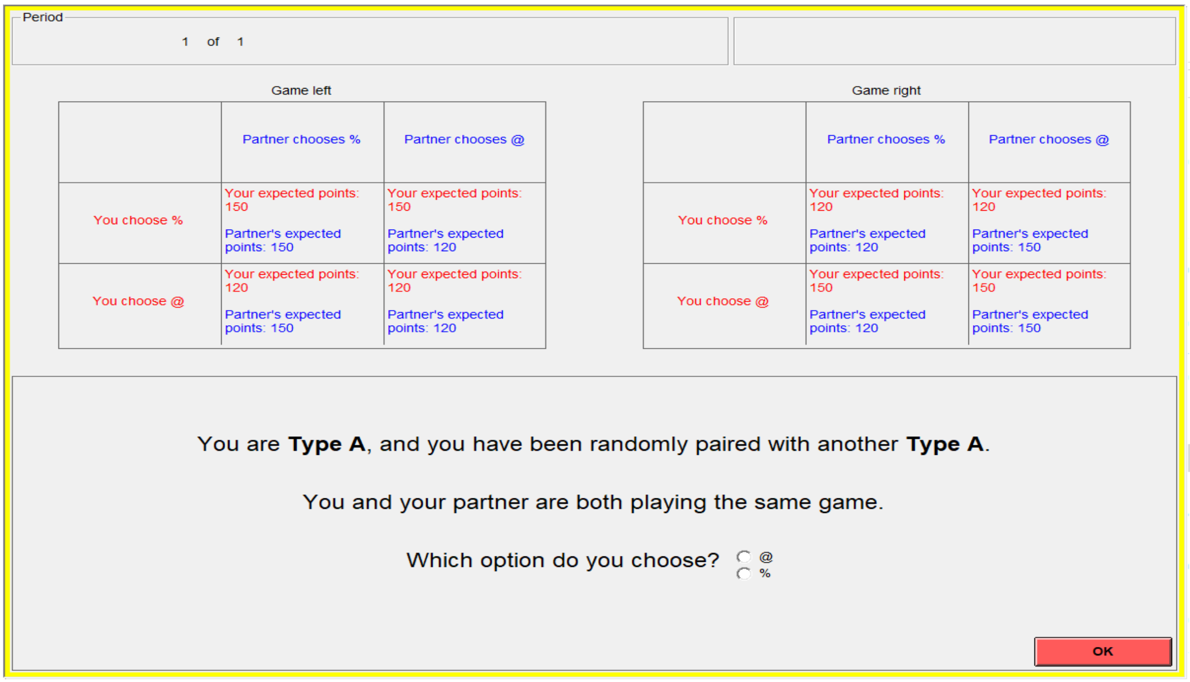

In this example immediately above, Option @ is listed first. Note, however, that the option listed first can change randomly from one period to the next. That is, you will sometimes see % listed first instead. This is true for everyone. In any given period some people will see % listed first, while others will see @ listed first. This means that you should pay close attention when choosing.

After making a choice and receiving a payoff, each Type A participant will immediately learn how many points he or she earned. Here is an example of a feedback screen that a Type A Participant might see:

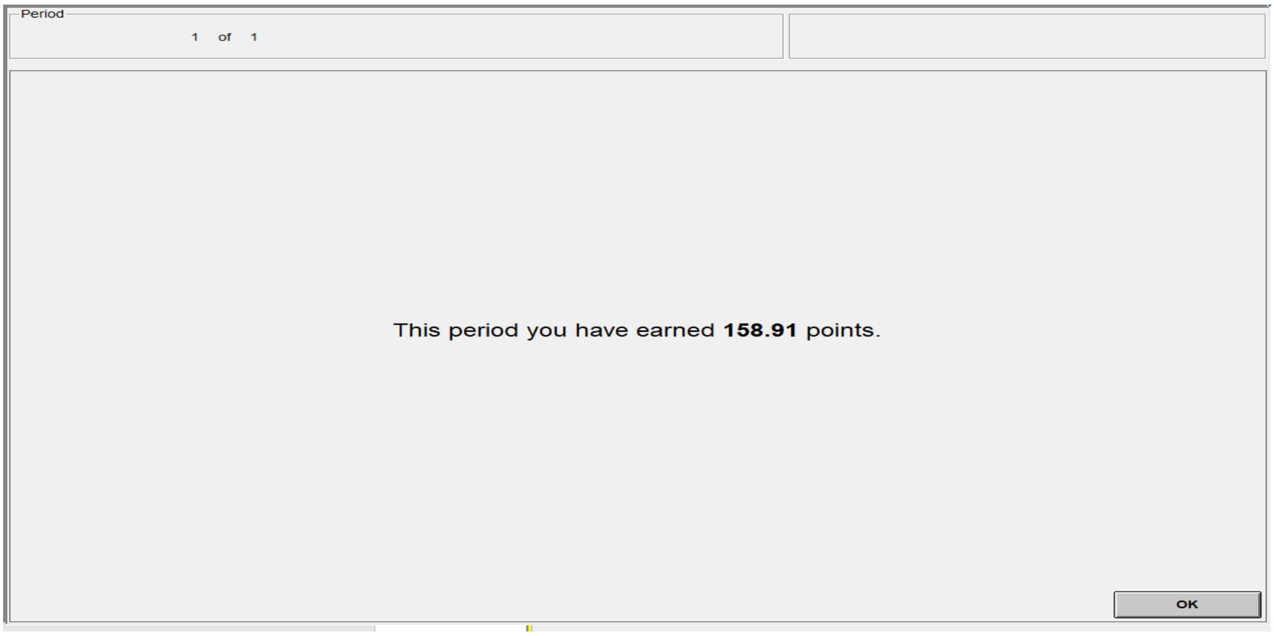

Type B Participants -> Type B Participants do NOT choose every period. Instead, they only choose in the final period of every block. In earlier periods of a block, Type B Participants simply wait. Here is an example choice screen that a Type B Participant might see in the final period of a block:

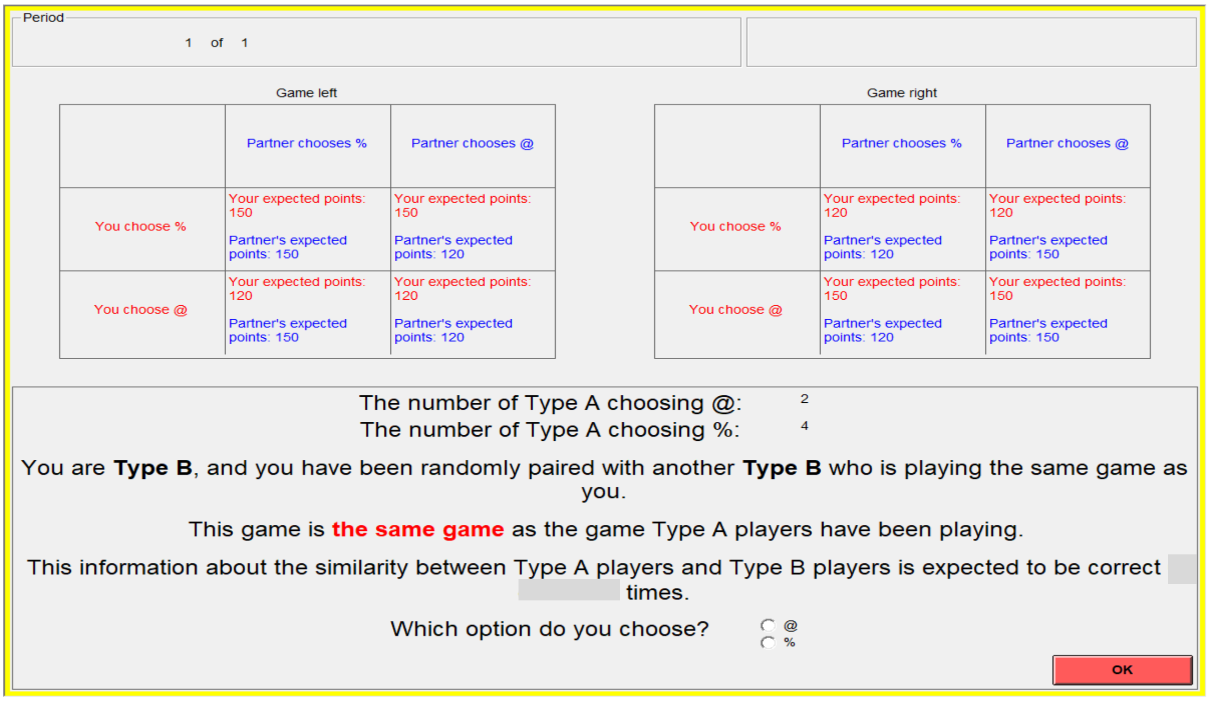

In this example immediately above, Option @ is listed first. Note, however, that the option listed first can change randomly from one period to the next. That is, you will sometimes see % listed first instead. This is true for everyone. In any given period some people will see % listed first, while others will see @ listed first. This means that you should pay close attention when choosing.

Importantly, when Type B Participants make a choice in the final period of a block, they will see the following information.

- The number of Type A Participants who chose option % and the number who chose option @ in the final (fourth) period of the block.
- A SIGNAL indicating if Type B Participants are playing the SAME game as Type A Participants or a DIFFERENT game (Game Left or Game Right). **IMPORTANTLY, you and your partner will always see the same signal.** As explained above, the computer begins each block by randomly choosing which game all Type A Participants play. It then randomly chooses the game that all Type B Participants play

separately. This means that Type B Participants **may or may not** be playing the same game as Type A Participants.

- The probability that the above signal is correct. The signal indicating if Type A Participants play the same game as Type B Participants is not always correct. This signal will only be correct with a certain probability. We do not tell you what this probability is here, but you will see it on the screen in **bold** every time you make a choice. The above screen shot provides an example in which we have blurred out the probability that the signal is correct. **IMPORTANTLY, the probability the signal is correct may change from one block to the next, so please pay attention every time you make a choice. The probability will ALWAYS be the same for you and your partner.**

The following table summarizes the relationship between games and signals:

	**Type A Participants play Game Left**	**Type A Participants play Game Right**
**Type B Participants play Game Left**	Correct signal: SAME<br><br>Incorrect Signal: DIFFERENT	Correct signal: DIFFERENT<br><br>Incorrect Signal: SAME
**Type B Participants play Game Right**	Correct Signal: DIFFERENT<br><br>Incorrect Signal: SAME	Correct Signal: SAME<br><br>Incorrect Signal: DIFFERENT

After making a choice, the Type B Participants will receive four separate pay-offs based on their choice and the game they are playing. Four separate random shock values will be added to these pay-offs. This means that, even though Type B Participants make fewer choices than Type A Participants, they have the exact same number of opportunities to earn points.

Type B Participants will not see the points they earn after choosing. Instead, they will only see the total number of points earned across all blocks at the very end of the study.

***

Final instructions

Once you have played the last block of the game, you will complete a short survey. Then, please wait until the researcher calls your seat number to receive your payment. Your earnings will not be told to any other participant.

Now please sign the consent form and answer the 10 pre-game questions. Raise your hand to alert the researcher when you are finished. Everyone must answer **ALL** pre-game questions correctly before we can begin.

Please keep these instructions to refer back to during the study.

If you have any further questions, feel free to contact the lead PhD researcher (Aysha Bellamy) at: **pejt007@live.rhul.ac.uk**

Comprehension questions

Please answer the following multiple-choice questions, by circling your chosen answer. Everyone must answer all 10 questions correctly before we can begin. You may use your instructions to help you:

***Q1*: Which of the following statements is true, in regards to the number of choices that each participant (Type A and B) makes?**

a) Type A participants make fewer choices than Type B participants.

b) Type A participants make 4X as many choices as Type B participants, but both types of participant have the same opportunities to earn points.

c) Type A Participants make 4X as many choices as Type B Participants, and thus earn 4X as much.

***Q2*: Which of the following statements is true, in regards to the points that you can earn if you were playing Game Left?**

a) You would expect to receive 150 points for choosing the % option.

b) You would expect to receive 120 points for choosing the % option.

***Q3*: Which of the following statements is true, in regards to the partner you are assigned to play the game with?**

a) I get paired with a new partner who is the same type as me in every block.

b) I will play with the same partner throughout the whole study.

c) I get paired with a new partner who is a different type from me in every block.

***Q4:* Which of the following statements is true, in regards to the things that may affect the points that you can earn?**

a) My points earned depend only on which game I am playing, and which option I choose.

b) My expected points depend on **both** the game I am playing and my partner's decisions, though the points that I can earn will also be affected by a random shock.

c) My expected points depend on which game I am playing, and which option I choose, though the points that I can earn will also be affected by a random shock.

***Q5:* Which of the following statements is true, in regards to whether you are playing the same game (Left or Right) as other participants?**

a) I always play the same game as my partner, though Type A and Type B Participants may play the same game or different games.

b) I play a different game to my partner, though Type A and Type B Participants play the same game.

c) All participants play the same game.

***Q6*: Which of the following statements is true, in regards to the feedback that Type A Participants receive?**

a) Type A participants see no information

b) Type A participants see the points made by other Type A participants, but not their own points.

c) Type A participants see their own points, but not the points of other Type A participants.

Q7: **If you are a Type B Participant, and the signal tells you that you're playing a different game from Type A Participants, is this information necessarily correct?**

a) This information is always correct.

b) This information will sometimes be correct, with a certain probability, and sometimes incorrect, with the remaining probability.

c) This information is never correct.

Q8: **If you are a Type B Participant, when will you see the probability that the signal (telling you that you are playing the same game as Type A's, or a different game) is correct?**

a) At the very end of the game.

b) After I make each choice.

c) It will be with the information on-screen before I make my choice.

Q9: **If you are a Type B Participant, you and your partner will always see the same signal indicating whether you're playing the same game as Type A Participants.**

a) True

b) False

Q10: **If you are a Type B Participant, the probability that this signal (see Q9) is correct will always be the same for you and your partner.**

a) True

b) False

Please now raise your hand and alert the researcher, who will check your answers to the multiple-choice comprehension questions.

Summary for participants (Experimenter will read aloud just after everyone has answered all comprehension questions correctly)

- Welcome to the main portion of the study! Today's session will consist of 88 periods divided into 22 blocks of 4 periods each. When we begin, the computer will randomly assign you to play as a Type A or Type B Participant.
- At the beginning of each block, you will be assigned to play with a partner of the same type as you for all periods in the block.
- For each choice, you will choose between two options (% or @). One option is expected to result in more points, though the option expected to be worth more depends on whether you are playing Game Left or Right. You do not know which of these games you are playing. The game can change between blocks, but not within blocks. You and your partner will always be playing the same game.
- Type A Participants choose every period and see the points earned immediately after every choice.
- Type B Participants only choose in the final period of each block. Before choosing, they will see (i) how many Type A Participants have chosen @ or %, (ii) a signal indicating whether they are playing the same game (Left or Right) as Type A Participants, and (iii) the probability that this signal is correct. If you are a Type B participant, you and your partner will always see the same information. The information may change, however, from one block to the next, so please pay attention to the on-screen information.
- If you have any questions, please ask them now. Remember not to talk to your fellow participants during the game.

✂ ____

Consent form continued

Client ID number: _______________

Thank you very much for reading the instructions sheet. If you have any questions, then feel free to raise your hand and ask the researcher.

If you are happy to take part in this study, then please sign below:

I have now read the instructions sheet and understood the study. I can confirm that I would still like to take part in this study.

Name in block letters:

____

Sign here: __________________________________ Date: ___________________________

<u>Comprehension questions answer sheet (for experimenter use only)</u>

This sheet contains only the correct options. Use this to check the participants understanding. If they have failed a certain question, direct them towards the relevant section of the instructions booklet.

***Q1*: b**

***Q2*: a**

***Q3*: a**

***Q4:* c**

***Q5:* a**

***Q6*: c**

***Q7:* b**

***Q8:* c**

***Q9:* a**

***Q10:* a**

Appendix 2B: Instructions for the coordination game.

Welcome! You are invited to play a game for approximately **1 ½ hours.** You can earn points during this study, which will be converted to money at the following rate:

25 points = ₹1

You will be paid a show-up fee of **₹100** on top of the money you earn. The choices that participants make during the study will be anonymous. This means you will not be able to identify the specific participants in the room who make certain choices, and none of the participants will be able to trace your choices back to you.

Please do not communicate with the other participants. If you have questions, or need to withdraw, then please raise your hand and tell the researcher.

Please read this instruction sheet **carefully**. You will then answer some questions to check that you have understood the study. We will not be able to proceed until everyone answers **all** questions correctly. You will also respond to a brief **survey** after the main study.

The study:

To begin, the computer will randomly choose six of you to be Type A Participants. Others will be Type B Participants. As explained later, your type will determine how often you make choices and the information you have when you do so. The study lasts for 88 periods. We will divide these 88 periods into 22 blocks of 4 periods each. Type A Participants will choose every period, which means they will make 88 choices. Type B Participants will only make a choice in the final period of each block, which means that Type B Participants will make 22 choices. Don't worry. Though Type A and Type B Participants do not make the same number of choices, they will have exactly the same opportunity to earn

points. We will explain this in detail later. The upper left-hand corner of your screen will have a counter that displays the current period you are in.

The games:

At the beginning of each block of 4 periods, the computer will randomly pair you with another participant of the same type to play a game. A Type A Participant will always be paired with another Type A, and a Type B Participant will always be paired with another Type B Participant. Every time you play, both you and your partner must choose one of two options, either option "%" or option "@".

Specifically, there are two games, which we call "Game Left" and "Game Right". At the beginning of each block, the computer will randomly pick which game Type A Participants play and which game Type B Participants play. The computer decides this completely randomly, giving four possible combinations, which are all equally likely to occur (each with a 1 in 4 probability). The four possibilities are:

(i) both types play Game Left,

(ii) Type A Participants play Game Left, Type B Participants play Game Right,

(iii) Type A Participants play Game Right, Type B Participants play Game Left,

(iv) both types play Game Right.

Note that we do not tell you if you are playing Game Left or Game Right. The following tables show you how your points will depend on the choices made by both you and your partner, for each of the games that you might play:

Game left

	Partner chooses %	Partner chooses @
You choose %	Your expected points: 325<br>Partner's expected points: 325	Your expected points: 100<br>Partner's expected points: 100
You choose @	Your expected points: 100<br>Partner's expected points: 100	Your expected points: 250<br>Partner's expected points: 250

Game right

	Partner chooses %	Partner chooses @
You choose %	Your expected points: 250<br>Partner's expected points: 250	Your expected points: 100<br>Partner's expected points: 100
You choose @	Your expected points: 100<br>Partner's expected points: 100	Your expected points: 325<br>Partner's expected points: 325

As you can see, one option is worth more points and will therefore result in you earning more money, on average, if you pick this option. The option (% or @) that is worth the most points is different depending on whether you are playing Game Left or Game Right.

You can also see that your payoff depends on what your **partner** chooses. Likewise, your partner's payoff depends on what you choose.

Lastly, points will also be affected by forces outside of your control, as in real life. The tables above shows the expected points you will earn, but random shocks will be applied to these values. These random shocks can lead you to earn **more OR less** points than the expected values shown in the tables. These random shocks can also lead your **partner** to earn **more OR less** points than those shown in the tables.

For example, assume you are playing Game Left. It is possible that you could earn more by choosing @ than you could by choosing % for a single choice, provided that your partner chooses the same option as you. It is more likely, however, that you and your partner will earn more points for choosing % than @, and so when choosing repeatedly % is highly likely to produce the most points.

Similarly, assume you are playing Game Right. It is possible that you could earn more by choosing % than by choosing @ for a single choice, provided that your partner chooses the same option as you. It is more likely, however, that you and your partner will earn more points for choosing @ than %, and so when choosing repeatedly @ is highly likely to produce the most points.

In summary, the points that you earn will depend on four things.

4. **The game being played:** Game Left or Game Right.
5. **The option you choose:** Option % or Option @.
6. **The option your partner chooses:** Option % or Option @.
7. **The random shock:** Random shocks are added to an expected payoff. Random shocks are independent of each other. This means that sometimes they will lead to more points than expected, and sometimes they lead to less points than expected.

IMPORTANT: The computer chooses the games being played at the beginning of each block. The games being played can change from one block to the next, and you will be paired with a different partner of the same type as you in each new block.

Type A vs. Type B Participants

As explained above, before the study begins, the computer will randomly select six participants to be Type A participants. Others will be Type B participants. Your type will not change.

Type A Participants -> Type A Participants choose every period, and each Type A Participant immediately sees the points he or she earns after making a choice.

IMPORTANT: The computer chooses the games being played at the beginning of each

block of four periods. Note that the game being played does **NOT** change across the four periods within a block. Here is an example choice screen for a Type A Participant:

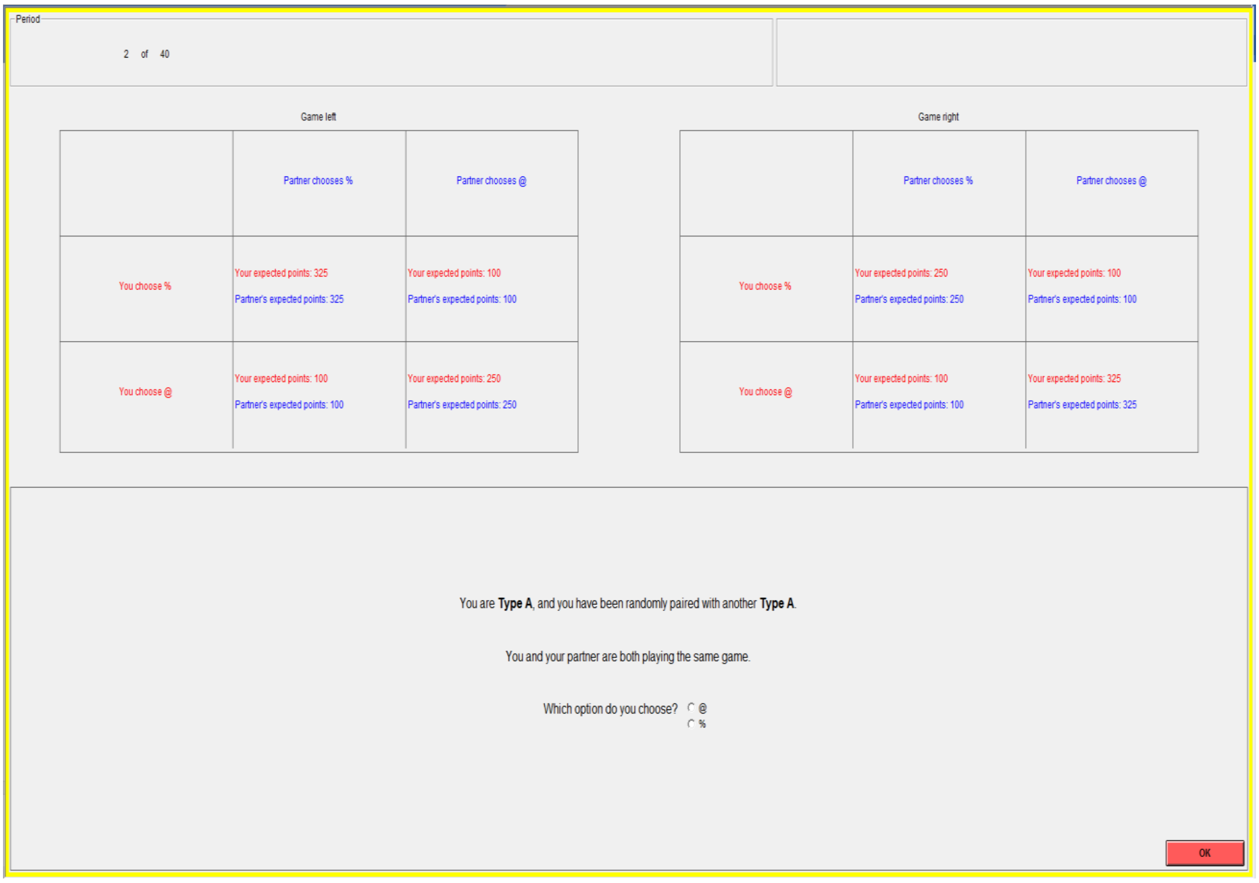

In this example immediately above, Option @ is listed first. Note, however, that the option listed first can change randomly from one period to the next. That is, you will sometimes see % listed first instead. This is true for everyone. In any given period some people will see % listed first, while others will see @ listed first. This means that you should pay close attention when choosing.

After making a choice and receiving a payoff, each Type A participant will immediately learn how many points he or she earned. Here is an example points screen a Type A Participants might see:

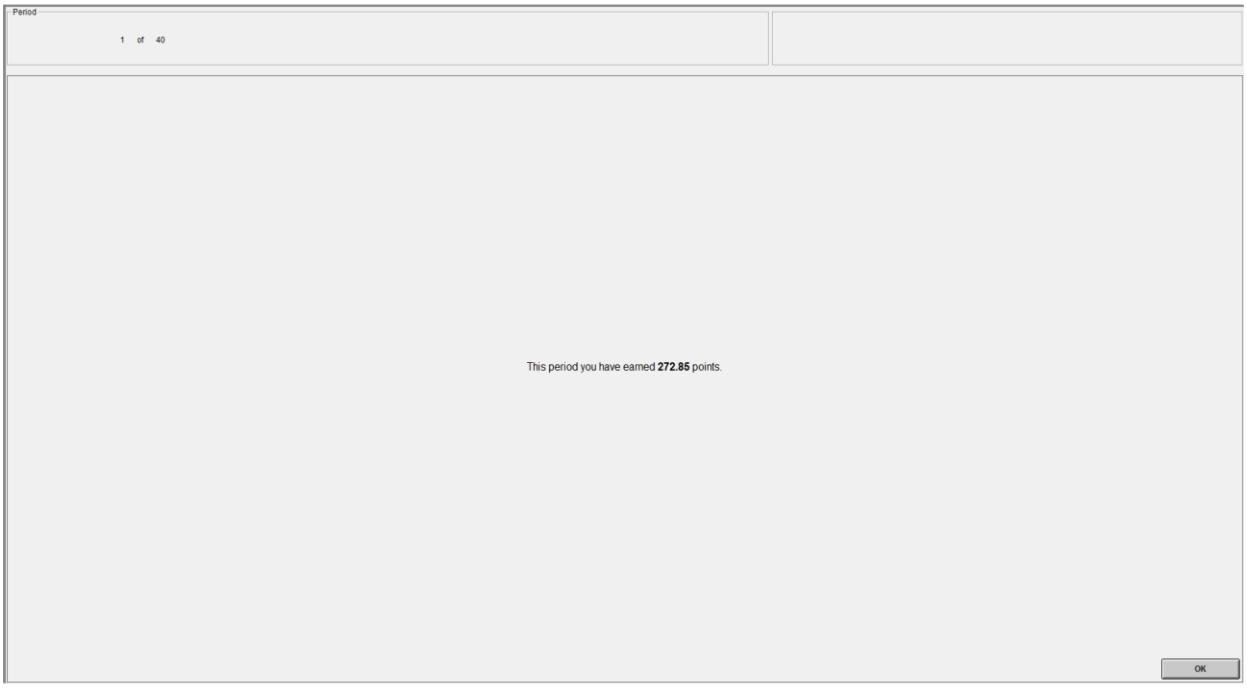

Type B Participants -> Type B Participants do NOT choose every period. Instead, they only choose in the final period of every block. In earlier periods of a block, Type B Participants simply wait. Here is an example of a choice screen a Type B Participant might see in the final period of a block:

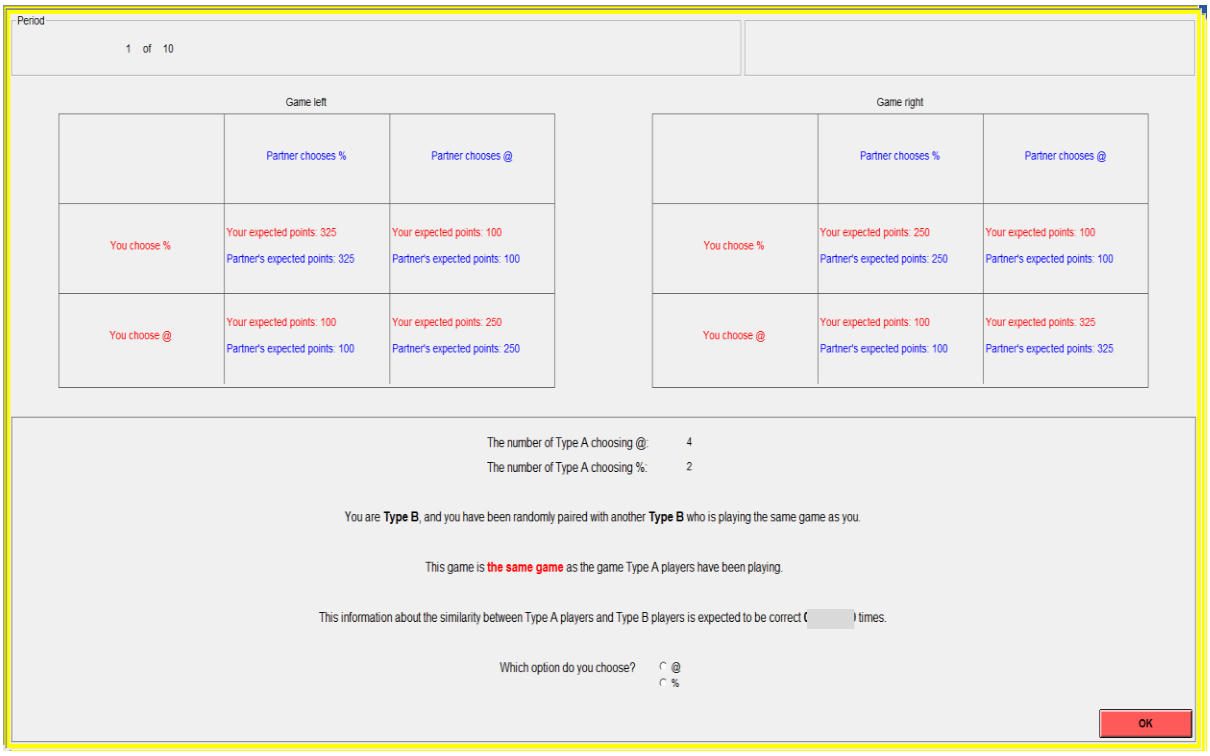

In this example immediately above, Option @ is listed first. Note, however, that the option listed first can change randomly from one period to the next. That is, you will sometimes see % listed first instead. This is true for everyone. In any given period some people will see % listed first, while others will see @ listed first. This means that you should pay close attention when choosing.

Importantly, when Type B Participants make a choice in the final period of a block, they will see the following information.

- The number of Type A Participants who chose option % and the number who chose option @ in the final (fourth) period of the block.
- A SIGNAL indicating if Type B Participants are playing the SAME game as Type A Participants or a DIFFERENT game (Game Left or Game Right). **IMPORTANTLY, you and your partner will always see the same signal.** As explained above, the

computer begins each block by randomly choosing which game all Type A Participants play. It then randomly chooses the game that all Type B Participants play separately. This means that Type B Participants **may or may** not be playing the same game as Type A Participants.

- The probability that the above signal is correct. The signal indicating if Type A Participants play the same game as Type B Participants is not always correct. We do not tell you what this probability is here, but you will see it on the screen in **bold** every time you make a choice. The screen shot provides an example in which we have blurred out the probability that the signal is correct. **IMPORTANTLY, the probability the signal is correct may change from one block to the next, so please pay attention every time you make a choice. The probability will ALWAYS be the same for you and your partner.**

The following table summarizes the relationship between games and signals:

	**Type A Participants play Game Left**	**Type A Participants play Game Right**
**Type B Participants play Game Left**	Correct signal: SAME<br><br>Incorrect Signal: DIFFERENT	Correct signal: DIFFERENT<br><br>Incorrect Signal: SAME
**Type B Participants play Game Right**	Correct Signal: DIFFERENT<br><br>Incorrect Signal: SAME	Correct Signal: SAME<br><br>Incorrect Signal: DIFFERENT

After making a choice, the Type B Participants will receive four separate pay-offs based on their choice, their partner's choice and the game they are playing. Four separate random shock values will be added to these pay-offs. This means that, even though Type B Participants make fewer choices than Type A Participants, they have the exact same number of opportunities to earn points.

Type B Participants will not see the points they earn after choosing. Instead, they will only see the total number of points earned across all their blocks at the very end of the study.

***

Final instructions

Once you have played the last block of the game, you will see a short survey you should then complete. This survey asks about how you played the game, and for some background information. You can answer the multiple-choice questions by using the mouse to select your chosen answer, and the remaining questions can be answered by clicking in the purple text box and typing in an answer. The study is finished once you answer this survey, and hit OK. You will then see a screen showing your total points and money earned. Please wait until the researcher calls your seat number to receive your payment. Your earnings will not be told to any other participant.

Now turn the page to sign the consent form and answer the 11 pre-game questions. Raise your hand to alert the researcher when you are finished. Everyone must answer **ALL** pre-game questions correctly before we can begin.

Please keep these instructions to refer back to during the study.

If you have any further questions, feel free to contact the lead PhD researcher (Aysha Bellamy) at: **pejt007@live.rhul.ac.uk**

Comprehension questions

Please answer the following multiple-choice questions, by circling your chosen answer. Everyone must answer all 11 questions correctly before we can begin. You may use your instructions to help you:

***Q1*: Which of the following statements is true, in regards to the number of choices that each participant (Type A and B) makes?**

a) Type A participants make fewer choices than Type B participants.

b) Type A participants make 4X as many choices as Type B participants, but both types of participant have the same opportunities to earn points.

c) Type A Participants make 4X as many choices as Type B Participants, and thus earn 4X as much.

***Q2*: Which of the following statements is true, in regards to the points that you can earn if you were to choose the same option as your partner whilst playing Game Left?**

a) You would expect to receive 325 points for choosing the % option.

b) You would expect to receive 250 points for choosing the % option.

***Q3:* Which of the following statements is true, in regards to the number of points that you should earn if you choose a different option to your partner?**

a) You will get 0 points.

b) You should get 100 points, but as earnings in real-life are affected by more than one decision, then a random shock applied to these points may mean that some participants receive more or less points than 100.

c) You should get 250 points, but as earnings in real-life are affected by more than one decision, then a random shock applied to these points may mean that some participants receive more or less points than 250.

***Q4*: Which of the following statements is true, in regards to the partner you are assigned to play the game with?**

a) I get paired with a new partner who is the same type as me in every block.

b) I will play with the same partner throughout the whole study.

c) I get paired with a new partner who is a different type from me in every block.

Q5: **Which of the following statements is true, in regards to the things that may affect the points that you can earn?**

a) My points earned depend only on which game I am playing, and which option I choose.

b) My expected points depend on **both** the game I am playing and which option me and my partner choose, though the points that I can earn will also be affected by a random shock.

c) My expected points depend on which game I am playing, and which option I choose, though the points that I can earn will also be affected by a random shock.

Q6: **Which of the following statements is true, in regards to whether you are playing the same game (Left or Right) as other participants?**

a) I always play the same game as my partner, though Type A and Type B Participants may play the same game or different games.

b) I play a different game to my partner, though Type A and Type B Participants play the same game.

c) All participants play the same game.

***Q7*: Which of the following statements is true, in regards to the feedback that Type A Participants receive?**

a) Type A participants see no information

b) Type A participants see the points made by other Type A participants, but not their own points.

c) Type A participants see their own points, but not the points of other Type A participants.

***Q8:* If you are a Type B Participant, and the signal tells you that you're playing a different game from Type A Participants, is this information necessarily correct?**

a) This information is always correct.

b) This information will sometimes be correct, with a certain probability, and sometimes incorrect, with the remaining probability.

c) This information is never correct.

***Q9:* If you are a Type B Participant, when will you see the probability that the signal (same game as Type A or different game) is correct?**

a) At the very end of the game.

b) After I make each choice.

c) It will be with the information on-screen before I make my choice.

Q10: **If you are a Type B Participant, you and your partner will always see the same signal indicating whether you're playing the same game as Type A Participants.**

a) True
b) False

Q11: **If you are a Type B Participant, the probability that this signal (see Q9) is correct will always be the same for you and your partner.**

A. True
B. False

Please now raise your hand and alert the researcher, who will check your answers to the multiple-choice comprehension questions.

Summary for participants (Experimenter will read aloud just after everyone has answered all comprehension questions correctly)

- Welcome to the main portion of the study! Today's session will consist of 88 periods divided into 22 blocks of 4 periods each. When we begin, the computer will randomly assign you to play as a Type A or Type B Participant.

- At the beginning of each block, you will be assigned to play with a partner of the same type as you for all periods in the block.

- For each choice, you will choose between two options (% or @). One option is expected to result in more points if both you and your partner choose it at the same time, though the option that is expected to be worth more depends on whether you are playing Game Left or Right. You do not know which of these games you are playing. The game can change between blocks, but not within blocks. You and your partner will always be playing the same game.

- Type A Participants choose every period and see the points earned immediately after every choice.

- Type B Participants only choose in the final period of each block. Before choosing, they will see (i) how many Type A Participants have chosen @ or %, (ii) a signal indicating whether they are playing the same game (Left or Right) as Type A Participants, and (iii) the probability that this signal is correct. If you are a Type B participant, you and your partner will always see the same information. The information may change, however, from one block to the next, so please pay attention to the on-screen information.

- If you have any questions, please ask them now. Remember not to talk to your

fellow participants during the game.

✂ ....

Consent form continued

Client ID number: ______________

Thank you very much for reading the instructions sheet. If you would like to clarify anything, then please raise your hand and ask a researcher. If for whatever reason you no longer wish to participate in this study, then please inform a researcher without signing this sheet.

If you are happy to take part in this study, then please sign below:

I have now read the instructions sheet and understood the study. I can confirm that I would still like to take part in this study.

Name in block letters:

____

Sign here: ________________________________ Date: _________________________

***

Comprehension questions answer sheet (for experimenter use only)

This sheet contains only the correct options. Use this to check the participants understanding. If they have failed a certain question, direct them towards the relevant section of the instructions booklet.

Q1: b

Q2: a

Q3: b

Q4: a

Q5: b

Q6: a

Q7: c

Q8: b

Q9: c

Q10: a

Q11: a

Appendix 3: The script used to run the experiment via Z-Tree version 3.5.

Appendix 3A: The link to the script used to run the game against nature, for a total of 30 participants in a session:

https://www.dropbox.com/sh/x5luey0br97kcb1/AABGw-2GQV-3HZHt0294wM_Va/session_2_14120218/session_2_14122018?dl=0&preview=bestChoiceWithinSubjectsIndependentSignals_totalOf30.ztt&subfolder_nav_tracking=1

Appendix S3B: **The link to the script used to run the coordination game, for a total of 30 participants in a session:**

https://www.dropbox.com/sh/x5luey0br97kcb1/AABbKs8hh_PW6krj4EXnePO1a/session_1_13120218/session_1_13122018?dl=0&preview=coorWithinSubjectsIndependentSignals_totalOf30.ztt&subfolder_nav_tracking=1

Appendix 4: Ethical requirements for study.

Appendix 4A: The debrief given to the participants at the end of both games:

ID number ___________

Thank you for taking part in **The gene-culture co-evolution of group identities** study via CESS. The data you have provided will be used in my PhD project. It will help us to understand how people make decisions based on social information. Specifically, we are interested in how your similarity to others during the game, and the reliability of this information (1/5/9 in 10 reliable) affected the way that Type B Participants used social information. We will also use your responses to the survey to further understand when and why people use social information when making decisions.

What happens now?

You have been paid for your time according to the points you earned plus a show-up fee. CESS will keep a copy of your raw data, and send us an anonymised copy. We will use this data in our analysis, which may be published, though I not identify any one's data specifically. Your unique subject ID codes will ensure that any published data is anonymous (i.e. cannot be traced back to you personally). Your data will be stored securely in accordance with the Data Protection Act 1988.

Thank you!

We are extremely grateful for the time you have given to take part in this study. If you would like any further information about the study, have concerns about your data, or are interested in any of the topics, then please contact me (Aysha Bellamy) with the contact details below. As you leave, **please remember to take this sheet with you.**

Aysha Bellamy *Work email*: pejt007@rhul.live.ac.uk

Appendix 4B: Proof of self-certified ethical clearance from Royal Holloway, University of London.

ROYAL HOLLOWAY UNIVERSITY OF LONDON

Ethics Review Details

You have chosen to self certify your project.	
Name:	Bellamy, Aysha (2017)
Email:	PEJT007@live.rhul.ac.uk
Title of research project or grant:	The gene-culture co-evolution of group identities.
Project type:	Royal Holloway postgraduate research project/grant
Department:	Psychology
Academic supervisor:	Dr. Charles Efferson
Email address of Academic Supervisor:	Charles.Efferson@rhul.ac.uk
Funding Body Category:	No external funder
Funding Body:	
Start date:	01/11/2018
End date:	01/09/2020

Research question summary:

The theory of gene-culture coevolution suggests that cultural evolution is an important influence on human behaviour. For cultural evolution to take place, one of the key assumptions in this field is that we preferentially learn from those of the same culture as ourselves. In experiments, this should translate to participants preferentially learning from a group of similar others (as similar people are more likely to belong to the same social group). Very few previous studies have investigated this assumption. Those studies that did investigate similarity have not taken into account that our ability to calculate our similarity to others may not always be reliable. These experiments will be the first to investigate a more-realistic, graded similarity-signal, to see how this impacts social-learning style preference. Conformity (or the disproportionate trend to adapt the same behaviour as the majority of a group) is thought to be important in homogenising cultural groups (i.e. making people of the same culture more similar), and in allowing costly levels of cooperation to emerge in human societies. Thus, conformity should be more likely in a group of similar others. Experiment 2 will also address whether conformity can allow cooperation to emerge, by investigating how participants learn from similar and different others during a Social Dilemma task structure.

In summary, the main research question behind these two studies is how people use similarity-information to modify their choice of social-learning strategy. Conformity and cooperation will also be investigated, and these studies will run in a lab in India in the aims of expanding our knowledge of social-learning strategies to those from a diverse range of cultures.

Note that instructions given below will be modified only slightly to accommodate the coordination and social dilemma games.

Research method summary:

Experiments run via Centre for Experimental Social Sciences (CESS) labs in Pune, India. Experiment 1 tests a group of participants en-masse in a best-choice or coordination game, and Experiment 2 tests a Social Dilemma game. Regardless of game-type, participants are assigned to play as individual- or social-learners. The aim of all three games is to choose between two options (@ or %). Individual-learners have four turns in a row, with immediate feedback from their decisions (points). Points are converted to pay with a random shock to reflect exogenous factors influencing decision-making. Individual-learners learn to choose the best option. Social-learners will not see the feedback based on their choices until the end of the game. Instead, they see the number of individual-learners who chose @ or % on their final turn. They also see a sentence telling them that they play the same or different game to individual-learners, and that this signal is likely to be correct only 1/5/9 in 10 of the time (representing an unreliable, chance guess and a reliable signal respectively). The same game means that the same option produces the highest points for individual- and social-learners (e.g. both choose @). At the end of the game, a survey asks social-learners what social-learning strategies they chose and why. Every participant gives key demographics, including age, gender, country of residence and length of time living there. This experiment is ran via Z-Tree, and individuals play at separately-screened computers, anonymising data. CESS provides anonymised files for analysis. The 'game' also involves paper-based instructions and multiple-choice questions to check understanding. The coordination game involves trying to sync your choice to another participant, with whom you cannot interact, whilst Social Dilemmas involve choosing between an individual-maximising strategy (defection) in favour of a strategy which is better when both parties choose it (cooperation).

Risks to participants

Does your research involve any of the below?

Children (under the age of 16),

No

Participants with cognitive or physical impairment that may render them unable to give informed consent,

No

Participants who may be vulnerable for personal, emotional, psychological or other reasons,

No

Participants who may become vulnerable as a result of the conduct of the study (e.g. because it raises sensitive issues) or as a result of what is revealed in the study (e.g. criminal behaviour, or behaviour which is culturally or socially questionable),

No

Participants in unequal power relations (e.g. groups that you teach or work with, in which participants may feel coerced or unable to withdraw),

No

Participants who are likely to suffer negative consequences if identified (e.g. professional censure, exposure to stigma or abuse, damage to professional or social standing),

No

Details,

Design and Data

Does your study include any of the following?

Will it be necessary for participants to take part in the study without their knowledge and/or informed consent at the time?,
No

Is there a risk that participants may be or become identifiable?,
No

Is pain or discomfort likely to result from the study?,
No

Could the study induce psychological stress or anxiety, or cause harm or negative consequences beyond the risks encountered in normal life?,
No

Does this research require approval from the NHS?,
No

If so what is the NHS Approval number,

Are drugs, placebos or other substances to be administered to the study participants, or will the study involve invasive, intrusive or potentially harmful procedures of any kind?,
No

Will human tissue including blood, saliva, urine, faeces, sperm or eggs be collected or used in the project?,
No

Will the research involve the use of administrative or secure data that requires permission from the appropriate authorities before use?,
No

Will financial inducements (other than reasonable expenses and compensation for time) be offered to participants?,
No

Is there a risk that any of the material, data, or outcomes to be used in this study has been derived from ethically-unsound procedures?,
No

Details,
Financial inducements are based on in-game performance. These cannot be negative (i.e. participants cannot lose pay) and points are set to be high, allowing for all participants to feel they are good at playing the game. Inducements will also have a random shock applied to them, so it will be made clear to participants that their pay earned in no way reflects how 'smart' they are at playing the game, so this use of financial incentives is not deemed to be unethical.

Risks to the Environment / Society

Will the conduct of the research pose risks to the environment, site, society, or artifacts?,
No

Will the research be undertaken on private or government property without permission?,
No

Risks to Researchers/Institution

Does your research present any of the following risks to researchers or to the institution?

Is there a possibility that the researcher could be placed in a vulnerable situation either emotionally or physically (e.g. by being alone with vulnerable, or potentially aggressive participants, by entering an unsafe environment, or by working in countries in which there is unrest)?,
No

Is the topic of the research sensitive or controversial such that the researcher could be ethically or legally compromised (e.g. as a result of disclosures made during the research)?,
No

Will the research involve the investigation or observation of illegal practices, or the participation in illegal practices?,

No

Could any aspects of the research mean that the University has failed in its duty to care for researchers, participants, or the environment / society?,
No

Is there any reputational risk concerning the source of your funding?,
No

Is there any other ethical issue that may arise during the conduct of this study that could bring the institution into disrepute?,
No

Details,

Declaration
By submitting this form, I declare that the questions above have been answered truthfully and to the best of my knowledge and belief, and that I take full responsibility for these responses. I undertake to observe ethical principles throughout the research project and to report any changes that affect the ethics of the project to the University Research Ethics Committee for review.

Certificate produced for user ID, PEJT007

Date:	05/11/2018 10:11
Signed by:	Bellamy, Aysha (2017)
Digital Signature:	Aysha Bellamy
Certificate dated:	11/5/2018 10:41:50 AM
Files uploaded:	Self-Assessment-851-2018-03-12-14-36-PEJT007.pdf<br>ins_india_BC_5Nov2018.docx

Ethics Self Assessment

Your answers indicate that you do not need ethical approval. If your research includes use of animals as research subjects, you will have been emailed separate guidance which must be followed before you begin your research. Should the circumstances of your research alter in any way please revisit this process to validate your project.

Applicant details

Declaration

By clicking the 'submit form' button, I declare that the questions above have been answered truthfully and to the best of my knowledge and belief, and that I take full responsibility for these responses. I undertake to observe ethical principles throughout the research project and to report any changes that affect the ethics of the project to the University Research Ethics Committee for review.

Project type:	Royal Holloway postgraduate research project/grant
Name:	Bellamy, Aysha (2017)
Email:	PEJT007@live.rhul.ac.uk
Academic supervisor:	Dr. Charles Efferson
Department:	Psychology
Title of research project or grant:	The gene-culture co-evolution of group identities.
Email address of Academic Supervisor:	Charles.Efferson@rhul.ac.uk
Funding Body Category:	No external funder
Funding Body:	

Information about the Research Project

Will the research project involve the use of human participants or human tissue (with or without their knowledge or consent at the time)?, No

Are the results of the research project likely to expose any person or community to physical or psychological harm?, No

Will the research project involve the use of animals as research subjects?, No

Will you have access to personal information that allows you to identify individuals or company confidential information (that is not covered by confidentiality terms within an agreement or by a separate confidentiality agreement)?, No

Does the conduct of the research project present a significant risk to the environment or society?, No

Are there any other ethical issues raised by this research project that in the opinion of the PI require further ethical review?, No

Does the PI believe that the results of this research could reasonably lead to legal action or negative press coverage, for which the PI would require University support?, No

Certificate produced for user ID PEJT007

Certificate dated 3/12/2018 2:36:57 PM

Appendix 5: All analysis scripts used in RStudio.

Note that the following includes the script to run all regressions (*analysis_CAG_GN_together_doubleCheck_30Ap21.R*), the function to create bootstrapped confidence intervals *(estAndBootImitationFunctions_CG_app.R*), and the script to create the graph seen in Figure 5 *(plotBoot_Graphs_BC_CG_Together_line.R*). The script called individualVariance_AnalaysisScatter2.R creates the heatmaps and scatterplots for assessing individual heterogeneity in social learning strategies. The script called *linearCombo_USE_2021.R* runs the linear combinations, and the raw data is available under *coordGame_GameAgainstNat.RData.*

These analysis scripts can be accessed at the following link:

OSF | What is a social-learning strategy, anyway?

Appendix 6: Histograms displaying individual learner data

These histograms show the frequency of demonstrators who chose the demonstrator optimum distributed across the final periods of all blocks for a) the game against nature and b) the coordination game. The positive skew in both histograms shows that the demonstrator was more likely to answer optimally than not. This confirms that they did provide varied– but on the whole accurate– social information to the social learners.

A

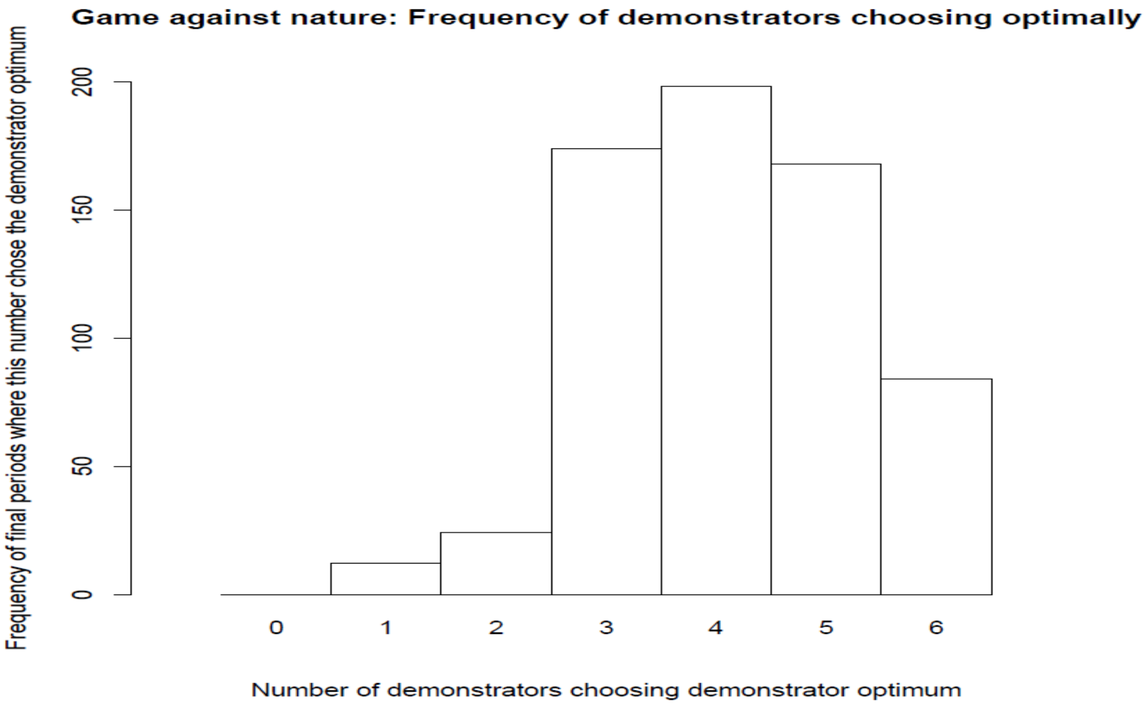

B

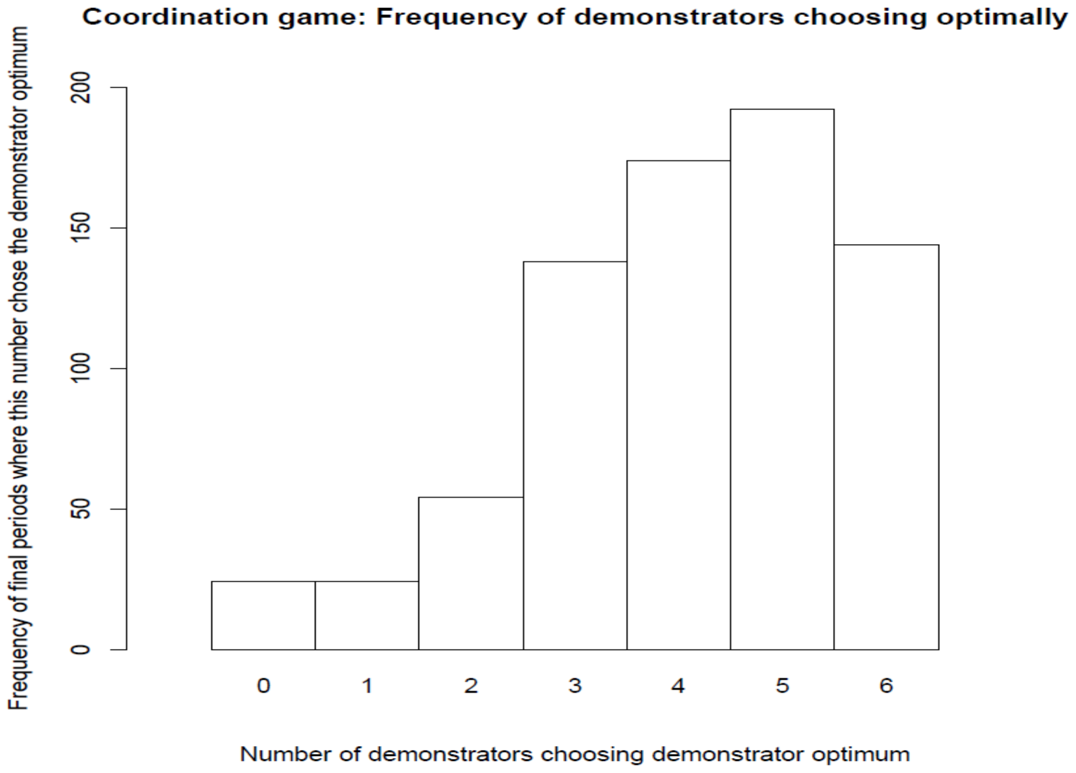
Coordination game: Frequency of demonstrators choosing optimally
Frequency of final periods where this number chose the demonstrator optimum
0
50
100
150
200
0
1
2
3
4
5
6
Number of demonstrators choosing demonstrator optimum

Appendix 7: The logistic regression modelling whether the social learners chose % with controls.

Predictors included (i) the centred number of demonstrators who chose % on their final period, (ii) each combination of the similarity and reliability information, minus the omitted category of reliably incorrect- similar signals, (iii) the interactions between each of these dummies and the centered proportion of demonstrators who chose % and (iv) demographic variables and other controls. The robust standard errors given in parentheses were clustered on the social learner to reflect the multiple observations gathered per learner.

**Parameter**	**Estimate (game against nature, with controls)**	**Estimate (coordination game, with controls)**
**Intercept**	-0.895<br>(1.267)<br>95% CI [-3.37, 1.58]	-0.168<br>(0.599)<br>95% CI [-1.34, 1.00]
**Centred proportion of demonstrators choosing %**	-1.530 **<br>(0.573)<br>95% CI [-2.65, --0.41]	-1.685 **<br>(0.557)<br>95% CI [-2.77, -0.60]
**Reliably incorrect-different dummy [signal indicates different and is correct with 0.1 probability]**	-0.177<br>(0.145)<br>95% CI [-0.46, 0.11]	-0.242<br>(0.208)<br>95% CI [-0.65, 0.16]
**Uninformative-same dummy [signal indicates same and is correct with 0.5 probability]**	-0.240<br>(0.147)<br>95% CI [-0.53, 0.05]	0.032<br>(0.191)<br>95% CI [-0.34, 0.40]
**Uninformative-different dummy [signal indicates different and is correct with 0.5 probability]**	-0.274 .<br>(0.160)<br>95% CI [-0.59, 0.04]	-0.244<br>(0.176)<br>95% CI [-0.59, 0.10]
**Reliably correct-same dummy [signal indicates same and is correct with 0.9 probability]**	0.013<br>(0.154)<br>95% CI [-0.29, 0.31]	-0.255<br>(0.219)<br>95% CI [-0.68, 0.17]

**Reliably correct-different dummy [signal indicates different and is correct with 0.9 probability]**	-0.039<br>(0.140)<br>95% CI [-0.31, 0.23]	-0.053<br>(0.193)<br>95% CI [-0.43, 0.32]
**Centred proportion of demonstrators choosing % X reliably incorrect-different dummy**	2.274 **<br>(0.750)<br>95% CI [0.81, 3.74]	3.352 ***<br>(0849)<br>95% CI [1.69, 5.01]
**Centred proportion of demonstrators choosing % X uninformative-same dummy**	4.167 ***<br>(0.803)<br>95% CI [2.60, 5.74]	4.093 ***<br>(0.838)<br>95% CI [2.46, 5.73]
**Centred proportion of demonstrators choosing % X uninformative-different dummy**	1.710 *<br>(0.693)<br>95% CI [0.36, 3.06]	1.883 *<br>(0.745)<br>95% CI [0.43, 3.43]
**Centred proportion of demonstrators choosing % X reliably correct-same dummy**	5.692 ***<br>(1.009)<br>95% CI [3.72, 7.66]	6.997 ***<br>(1.132)<br>95% CI [4.79, 9.21]
**Centred proportion of demonstrators choosing % X reliably correct-different dummy**	-0.473<br>(0.643)<br>95% CI [-1.73, 0.78]	-0.356<br>(0.700)<br>95% CI [-1.72, 1.01]
**Percentage as optimal dummy [signal indicates percentage is optimal option]**	0.044<br>(0.089)<br>95% CI [-0.13, 0.22]	0.020<br>(0.103)<br>95% CI [-0.18, 0.22]
**Age**	0.008<br>(0.030)<br>95% CI [-0.05, 0.07]	0.033<br>(0.028)<br>95% CI [-0.02, 0.09]
**Gender**	-0.104<br>(0.161)<br>95% CI [-0.42, 0.21]	-0.04<br>(0.155)<br>95% CI [-0.34, 0.26]
**Time in residence**	0.007<br>(0.011)<br>95% CI [-0.01, 0.03]	-0.011<br>(0.009)<br>95% CI [-0.03, 0.01]
**Block Index**	-0.016 *<br>(0.006)	0.001<br>(0.007)<br>95% CI [-0.01, 0.01]

	95% CI [-0.03, 0.00]	
**India dummy**	1.214<br>(1.066)<br>95% CI [-0.87, 3.30]	-

The asterisks denote the level of significance of our p values, with the following key:

*** (p<0.001)

** (p<0.01)

* (p<0.05)

· (trend: p=0.05 – 0.10 significance)

Note that the only significant control predictor was Block Index for the game against nature. That is, the social learners were less likely to choose % in the later blocks of the game against nature. The analysis script in appendix 5 confirmed a non-significant trend for Game Version Left to be less likely to be played in the later periods of the sessions. That is, % was less likely to be optimal as the games progressed and so the social learners' choices may be appropriate to the random effects of the code used.

Appendix 8: Linear combinations

Appendix 8A: The code used to calculate the linear combinations.

The code to calculate the linear combinations *(linearCombo_USE_2021.R*) can be found at the following link:

OSF | What is a social-learning strategy, anyway?

Appendix 8B: The linear combinations produced for social-learner choices for the game against nature.

Note there is a significant difference between the social learners' strategies in response to all levels of social information, with the exception of different-reliably incorrect versus different-uninformative signals. The social learners' strategies to these two signals were not significantly distinct (see Figure 5D and 5E in the main text).

Second-order (similar versus different)

- Reliably incorrect signals (0/6:$F(1,2628) = 11.33$, $p=0.0008$;

  6/6: $F(1,2628) = 6.39$, $p=0.01$).

  5/6: $F(1,2628)=4.59$, $p=0.03$)

  4/6: $F(1,2628)=1.33$, $p=0.25$
- Uninformative signals (0/6: $F(1,2628)=9.88$, $p=0.002$;

  6/6: $F(1,2628)=9.63$, $p=0.002$.

  5/6: $F(1,2628)=8.00$, $p=0.005$

  4/6: $F(1,2628)=4.35$, $p=0.04$
- Reliably correct signals (0/6: $F(1,2628) = 40.79$, $p<0.001$;

  6/6: $F(1,2628)=37.00$, $p<0.001$).

5/6: $F(1,2628)=32.07$, $p<0.001$

4/6: $F(1,2628)=19.54$, $p<0.001$

<u>Third-order (comparing reliability)</u>

- Similar, reliably incorrect vs uninformative (0/6: $F(1,2628) = 32.25$, $p<0.001$;

  6/6: $F(1,2628) = 17.25$, $p<0.001$).

  5/6: $F(1,2628)=12.84$, $p=0.003$.

  4/6: $F(1,2628)=4.60$, $p=0.03$
- Similar, reliably incorrect vs correct (0/6: $F(1,2628) = 32.66$, $p<0.001$;

  6/6: $F(1,2628) = 28.38$, $p<0.001$).

  5/6: $F(1,2628)=25.07$, $p<0.001$

  4/6: $F(1,2628) =16.13$, $p<0.001$
- Similar, uninformative vs reliably correct (0/6: $F(1,2628) = 2.02$, $p=0.16$;

  6/6: $F(1,2628)=5.02$, $p=0.025$.

  5/6: $F(1,2628)=5.17$, $p=0.02$

  4/6: $F(1,2628)=5.04$, $p=0.02$
- Different, reliably incorrect vs uninformative (0/6: $F(1,2628) = 0.38$, $p=0.54$;

  6/6: $F(1,2628)=2.15$, $p=0.14$

  5/6: $F(1,2628)=2.16$, $p=0.14$

  4/6: $F(1,2628)=1.66$, $p=0.20$
- Different, reliably incorrect vs correct (0/6: $F(1,2628) = 16.24$, $p<0.001$;

  6/6: $F(1,2628) = 12.72$, $p=0.0004$).

  5/6: $F(1,2628)= 9.86$, $p=0.002$

  4/6: $F(1,2628)=3.72$, $p=0.054$
- Different, uninformative vs reliably correct (0/6: $F(1,2628) = 14.42$, $p=0.0001$;

  6/6: $F(1,2628) = 7.42$, $p=0.006$)

5/6: F(1,2628)=4.37, p=0.04

4/6: F(1,2628)=0.53, p=0.47

Appendix 8C: The linear combinations produced for the social-learner choices for the coordination game.

Note that the social learner shows a significantly distinct strategy for each level of the similarity and reliability information.

<u>Second-order (similar versus different)</u>

- Reliably incorrect signals (0/6: F(1,2372)=11.14, p=0.0009;

  6/6: F(1,2372) = 14.71, p<0.001

  5/6: F(1,2372)=12.14, p=0.0005

  4/6: F(1,2372)=4.66, p=0.03
- Uninformative signals (0/6: F(1,2372)=9.54, p=0.002;

  6/6: F(1,2372) = 16.09, p<0.001

  5/6: F(1,26372)=14.53, p=0.0001

  4/6: F(1,2372)=8.47,p=0.004
- Reliably correct signals (0/6: F(1,2372) =46.85, p<0.001;

  6/6: F(1,2372) = 50.89, p<0.001

  5/6: F(1,2372)=44.78, p<0.001

  4/6:F(1,2372)=25.73, p<0.001

Third-order (comparing reliability)

- Similar, reliably incorrect vs uninformative (0/6: F(1,2372) = 23.89, p<0.001;

  6/6: F(1,2372)=31.77, p<0.001

  5/6: F(1,2372)=28.14, p<0.001

  4/6: F(1,2372)=15.63, p<0.001
- Similar, reliably incorrect vs correct (0/6: F(1,2372)=37.95, p<0.001;

  6/6: F(1,2372) = 41.69, p<0.001

  5/6: F(1,2372)=35.73, p<0.001

  4/6: F(1,2372)=18.7, p<0.001
- Similar, uninformative vs reliably correct (0/6: F(1,2372) =5.85, p=0.02;

  6/6: F(1,2372)=7.99, p=0.005

  5/6: F(1,2372)=6.79, p=0.009

  4/6: F(1,2372)=2.83, p=0.09
- Different, reliably incorrect vs uninformative (0/6: F(1,2372)=3.02, p=0.08;

  6/6:F(1,2372)=4.45, p=0.04

  5/6: F(1,2372)=3.25, p=0.07

  4/6: F(1,2372)=0.97,p=0.33
- Different, reliably incorrect vs correct (0/6: F(1,2372) = 14.66, p<0.001;

  6/6:F(1,2372)=26.77, p<0.001

  5/6: F(1,2372)=23.16, p<0.001

  4/6: F(1,2372)=9.14, p=0.003
- Different, uninformative vs reliably correct (0/6: F(1,2372) =8.90, p=0.003;

  6/6: F(1,2372)=19.56, p<0.001

  5/6: F(1,2372)=14.84, p=0.0001

  4/6: F(1,2372)=4.60, p=0.03

Appendix 9: The regressions predicting social-learner optimality with control predictors included.

Appendix 9A: Logistic regression modelling whether the social learner chose the social learner optimum for the game against nature, with controls.

Predictors included: (i) the centered proportion of demonstrators who chose the demonstrator optimum, (ii) dummies for each combination of similarity and reliability information, minus the omitted category of reliably incorrect- similar signals, (iii) interactions between each of these dummies and the centered proportion of demonstrators who chose the demonstrator optimum and (iv) demographic variables and other control predictors. Robust standard error clustered on social learner.

Parameter	Estimate (game against nature, all signals, controls)	Estimate (game against nature, correct signals, controls)	Estimate (game against nature, incorrect signals, controls)
**Intercept**	-0.250 (0.478) 95% CI [-1.18, 0.68]	-0.408 (0.474) 95% CI [-1.33, 0.52]	-0.232 (0.4442) 95% CI [-1.10, 0.63]
**Centred proportion of demonstrators choosing demonstrator optimum**	0.270 (0.637) 95% CI [-0.97, 1.51]	-0.428 (0.308) 95% CI [-1.03, 0.17]	1.537 *** (0.323) 95% CI [0.91, 2.17]
**Reliably incorrect-different dummy [signal indicates different and is correct with 0.1 probability]**	-0.077 (0.182) 95% CI [-0.43, 0.28]	-0.124 (0.546) 95% CI [-1.19, 0.94]	-0.008 (0.131) 95% CI [-0.26, 0.25]
**Uninformative-same dummy [signal indicates same and is correct with 0.5 probability]**	-0.200 (0.202) 95% CI [-0.59, 0.19]	-0.177 (0.218) 95% CI [-0.60, 0.25]	-0.109 (0.215) 95% CI [-0.53, 0.31]

**Uninformative-different dummy [signal indicates different and is correct with 0.5 probability]**	-0.157 (0.195) 95% CI [-0.54, 0.22]	-0.136 (0.187) 95% CI [-0.50, 0.23]	-0.353 * (0.178) 95% CI [-0.70, -0.01]
**Reliably correct-same dummy [signal indicates same and is correct with 0.9 probability]**	-0.205 (0.226) 95% CI [-0.65, 0.24]	0.044 (0.170) 95% CI [-0.29, 0.38]	0.980 . (0.575) 95% CI [-2.10, 0.14]
**Reliably correct-different dummy [signal indicates different and is correct with 0.9 probability]**	-0.055 (0.194) 95% CI [-0.43, 0.32]	0.094 (0.145) 95% CI [-0.19, 0.38]	-0.141 (0.529) 95% CI [-1.17, 0.89]
**Centred proportion of demonstrators choosing optimum X reliably incorrect-different dummy**	0.079 (0.814) 95% CI [-1.51, 1.67]	-1.348 (2.107) 95% CI [-5.46, 2.77]	-1.040 * (0.526) 95% CI [-2.07, -0.01]
**Centred proportion of demonstrators choosing optimum X uninformative-same dummy**	-0.698 (0.836) 95% CI [-2.33, 0.93]	3.496 *** (1.027) 95% CI [1.49, 5.50]	-4.255 *** (0.942) 95% CI [-6.09, -2.41]
**Centred proportion of demonstrators choosing optimum X uninformative-different dummy**	-0.747 (0.806) 95% CI [-2.32, 0.83]	0.076 (0.690) 95% CI [-1.27, 1.42]	-2.385 *** (0.525) 95% CI [-3.41, -1.36]
**Centred proportion of demonstrators choosing optimum X reliably correct-same dummy**	2.346 * (0.920) 95% CI [0.55, 4.14]	4.016 *** (0.892) 95% CI [2.27, 5.76]	-4.356 (2.835) 95% CI [-9.90, 1.18]
**Centred proportion of demonstrators choosing optimum X reliably correct-different dummy**	0.766 (0.781) 95% CI [-0.76, 2.29]	1.737 ** (0.617) 95% CI [0.53, 2.94]	-4.518 . (2.589) 95% CI [-9.57, 0.54]
**Percentage as optimal dummy [signal indicates percentage is optimal option]**	0.398 ** (0.145) 95% CI [0.11, 0.68]	0.458 ** (0.148) 95% CI [0.17, 0.75]	0.390 ** (0.150) 95% CI [0.10, 0.68]
**Age**	0.007 (0.184) 95% CI [-0.03, 0.04]	0.009 (0.019) 95% CI [-0.03, 0.05]	0.003 (0.018) 95% CI [-0.03, 0.04]
**Gender**	0.164 . (0.089)	0.162 . (0.091)	0.174 (0.090)

	95% CI [-0.01, 0.34]	95% CI [-0.02, 0.0]	95% CI [0, 0.35]
**Time in residence**	-0.011 (0.007)	-0.010 (0.007)	-0.012 (0.007)
	95% CI [-0.03, 0.00]	95% CI [-0.02, 0]	95% CI [-0.03, 0]
**Block Index**	0.009 (0.006)	0.008 (0.007)	0.008 (0.006)
	95% CI [0.0, 0.02]	95% CI [0, 0.02]	95% CI [-0.01, 0.02]
**India dummy**	0.065 (0.262)	0.024 (0.257)	0.099 (0.246)
	95% CI [-0.45, 0.58]	95% CI [-0.48, 0.53]	95% CI [-0.38, 0.58]

The asterisks denote the level of significance of our p values, with the following key:

**** (p<0.001)*

*** (p<0.01)*

** (p<0.05)*

· *(trend: p=0.05 – 0.10 significance)*

For the blocks containing just incorrect similarity signals (right-hand column of Table 8A), the social learners' were less likely to coordinate on their social learner optimum in response to uninformative-different blocks. They were also less likely to answer optimally as more demonstrators coordinated on the demonstrator optimum for reliably incorrect-different blocks. This suggests that the learner shows a meaningful adjustment to both reliably incorrect and uninformative signals from different others, but this only pays off when the blocks happen to give incorrect information. Otherwise, the effects match those depicted in table 4 of the main text.

The only significant control predictor is Percentage as optimal dummy. That is, the social learners were significantly more likely to answer optimally when % happened to be optimal. This suggests a bias to choose % rather than @ throughout the game against nature. This suggests an arbitrary preference for the % symbol.

Appendix 9B: The logistic regression modelling whether the social learners chose the social learner optimum for the coordination game, with controls.

Predictors included: (i) the centered proportion of demonstrators who chose the demonstrator optimum, (ii) dummies for each combination of similarity and reliability information, minus the omitted category of reliably incorrect- similar signals, (iii) interactions between each of these dummies and the centered proportion of demonstrators who chose the demonstrator optimum and (iv) demographic variables and control predictors. Robust standard error clustered on social learner.

Parameter	Estimate (Coordination game, all signals, controls)	Estimate (Coordination game, correct signals, controls)	Estimate (coordination game, incorrect signals, controls
**Intercept**	-0.633<br>(0.518)<br>95% CI [-1.64, 0.38]	-0.439<br>(0.491)<br>95% CI [-1.40, 0.52]	-0.328<br>(0.489)<br>95% CI [-1.28, 0.63]
**Centred proportion of demonstrators choosing demonstrator optimum**	1.184 *<br>(0.493)<br>95% CI [0.22, 2.15]	0.067<br>(0.232)<br>95% CI [-0.39, 0.52]	1.879 ***<br>(0.305)<br>95% CI [1.28, 2.47]
**Reliably incorrect-different dummy [signal indicates different and is correct with 0.1 probability]**	0.244<br>(0.178)<br>95% CI [-0.10, 0.59]	0.903<br>(0.663)<br>95% CI [-0.39, 2.20]	0.203<br>(0.159)<br>95% CI [-0.11, 0.51]
**Uninformative-same dummy [signal indicates same and is correct with 0.5 probability]**	-0.086<br>(0.184)<br>95% CI [-0.45, 0.27]	-0.488 *<br>(0.223)<br>95% CI [-0.92, -0.05]	-0.274<br>(0.217)<br>95% CI [-0.70, 0.15]
**Uninformative-different dummy [signal indicates different and is correct with 0.5 probability]**	0.020<br>(0.190)<br>95% CI [-0.35, 0.39]	-0.368<br>(0224)<br>95% CI [-0.81, 0.07]	0.374<br>(0.235)<br>95% CI [-0.08, 0.83]
**Reliably correct-same dummy [signal indicates same and is correct with 0.9 probability]**	0.678 **<br>(0.252)	0.216<br>(0.547)	0.182<br>(0.297)

	95% CI [0.19, 1.17]	95% CI [-0.28, 0.71]	95% CI [-0.40, 0.76]
**Reliably correct-different dummy [signal indicates different and is correct with 0.9 probability]**	-0.033 (0.189) 95% CI [-0.40, 0.34]	-0.143 (0.148) 95% CI [-0.43, 0.15]	0.928 (1.039) 95% CI [-1.10, 2.96]
**Centred proportion of demonstrators choosing optimum X reliably incorrect-different dummy**	-0.218 (0.681) 95% CI [-1.55, 1.11]	-2.615 (2.02) 95% CI [-6.56, 1.33]	-0.707 (0.577) 95% CI [-1.83, 0.42]
**Centred proportion of demonstrators choosing optimum X uninformative-same dummy**	-1.214 * (0.609) 95% CI [-2.40, -0.02]	3.378 *** (0.835) 95% CI [1.75, 5.01]	-4.044 *** (0.812) 95% CI [-5.63, -2.46]
**Centred proportion of demonstrators choosing optimum X uninformative-different dummy**	-0.876 (0.673) 95% CI [-2.19, 0.44]	0.664 (0.749) 95% CI [-0.80, 2.13]	-2.359 * (0.950) 95% CI [-4.21, -0.50]
**Centred proportion of demonstrators choosing optimum X reliably correct-same dummy**	-0.209 (0.835) 95% CI [-1.84, 1.42]	4.313 *** (1.143) 95% CI [2.08, 6.54]	-6.955 *** (1.761) 95% CI [-10.39, -3.52]
**Centred proportion of demonstrators choosing optimum X reliably correct-different dummy**	0.654 (0.635) 95% CI [-0.59, 1.89]	1.934 *** (0.572) 95% CI [0.82, 3.05]	-6.080 * (2.739) 95% CI [-11.43, -0.73]
**Percentage as optimal dummy [signal indicates percentage is optimal option]**	0.706 *** (0.14) 95% CI [0.41, 1.00]	0.656 *** (0.144) 95% CI [0.37, 0.94]	0.660 *** (0.148) 95% CI [0.37, 0.95]
**Age**	0.003 (0.235) 95% CI [-0.04, 0.05]	0.00002 (0.023) 95% CI [-0.05, 0.05]	-0.011 (0.023) 95% CI [-0.06, 0.03]
**Gender**	0.128 (0.108) 95% CI [-0.08, 0.34]	0.129 (0.109) 95% CI [-0.08, 0.34]	0.094 (0.112) 95% CI [-0.13, 0.31]
**Time in residence**	-0.005 (0.008) 95% CI [-0.02, -0.01]	-0.0018 (0.751) 95% CI [-0.02, 0.01]	0.007 (0.008) 95% CI [-0.01, 0.02]

**Block Index**	0.011 (0.007) 95% CI [0, 0.02]	0.014 * (0.007) 95% CI [0, 0.03]	0.012 . (0.007) 95% CI [0, 0.03]

The asterisks denote the level of significance of our p values, with the following key:
**** (p<0.001)*
*** (p<0.01)*
** (p<0.05)*
· *(trend: p=0.05 – 0.10 significance)*

On blocks with incorrect similarity information (right-hand column of Table 8B), the social learners were less likely to answer optimally as more demonstrators did for uninformative-different signals, suggesting that the learners in the full sessions only did show a meaningful adjustment to this signal.

On blocks with correct signals (middle column of Table 8B), the social learners were less likely to coordinate on the social-learner optimum for reliably incorrect-different signals, which suggests that the social learners treat these signals as if they were always incorrect.

On blocks with all correct and incorrect signals collapsed together (left-hand column of Table 8B), the social learners are more likely to coordinate on the social learner optimum as more demonstrators answer optimally (Centered proportion of demonstrators choosing the demonstrator optimum effect). This suggests that the learners respond meaningfully to frequency-dependent social information for the omitted category of reliably incorrect signals from similar others. They are also the most likely to answer optimally to blocks with reliably correct-similar signals. All other significant effects match those displayed in table 5 of the main text, thus confirming similarity between the basic model and full model with controls.

Finally, there is a significant bias to answer optimally if percentage happened to be optimal (Percentage as optimal dummy effect). This suggests a bias whereby the learner followed a rule of ‘just choose %’, perhaps due to the pressure to coordinate. Secondly, the social learners were more likely to answer optimally as the blocks progressed for blocks

where the similarity signals happened to be correct only (Block index effect). This suggests a rate of learning over the blocks as the learners became more used to the coordination game.

Appendix 10: Linear combinations comparing the social-learners' ability to respond optimally across each level of the social information, for the game against nature.

Note that the social learners found it easier to respond optimally to reliably-similar than reliably-different others and found it easier to respond to reliably correct-similar than uninformative-similar or reliably incorrect-similar others. These biases persist for all signals and correct signals.

<u>All signals, game against nature</u>

<u>Second-order social information</u>

Reliably incorrect: 0/6: $F(1,2628)= 0.36$, $p=0.55$

6/6: $F(1,2628)=0.02$, $p=0.88$

5/6: $F(1,2628)=0.003$, $p=0.96$

4/6: $F(1,2628)=0.24$, $p=0.62$

Uninformative: 0/6: $F(1,2628)=0.06$, $p=0.81$

6/6: $F(1,2628)<0.001$, $p=0.98$

5/6: $F(1,2628)=0.02$, $p=0.88$

4/6: $F(1,2628)=0.12$, $p=0.73$

Reliably correct: 0/6: $F(1,2628)=4.72$, $p=0.03$

6/6: $F(1,2628)=4.86$, $p=0.03$

5/6: $F(1,2628)=3.48$, $p=0.06$

4/6: $F(1,2628)=0.26$, $p=0.61$

Third-order social information

Similar others, reliably incorrect versus uninformative. 0/6: $F(1,2628)=0.13$, $p=0.72$

6/6: $F(1,2628)=3.41$, $p=0.07$

5/6: $F(1,2628)=4.76$, $p=0.03$

4/6: $F(1,2628)=4.50$, $p=0.03$

Similar others, reliably incorrect versus correct: 0/6: $F(1,2628)=5.91$, $p=0.02$

6/6: $F(1,2628)=8.86$, $p=0.002$

5/6: $F(1,2628)=7.30$, $p=0.007$

4/6: $F(1,2628)=0.88$, $p=0.35$

Similar others, uninformative vs reliably correct: 0/6: $F(1,2628)=9.74$, $p=0.002$

6/6: $F(1,2628)=22.10$, $p<0.001$

5/6: $F(1,2628)=22.31$, $p<0.001$

4/6: $F(1,2628)=10.52$, $p=0.001$

Different others, reliably incorrect versus uninformative: 0/6: $F(1,2628)=1.95$, $p=0.16$

6/6: $F(1,2628)=5.62$, $p=0.02$

5/6: $F(1,2628)=5.03$, $p=0.02$

4/6: $F(1,2628)=1.89$, $p=0.17$

Different others, reliably incorrect versus correct: 0/6: $F(1,2628)=0.04$, $p=0.84$

6/6: $F(1,2628)=0.83$, $p=0.36$

5/6: $F(1,2628)=1.09$, $p=0.30$

4/6: $F(1,2628)=1.10$, $p=0.29$

Different others, uninformative versus reliably correct: 0/6: $F(1,2628)=1.88$, $p=0.17$

6/6: $F(1,2628)=10.14$, $p=0.01$

5/6: F(1,2628) =11.89, p=0.0006

4/6: F(1,2628)=6.74, p=0.009

Just correct signals, game against nature

Second order

Reliably incorrect. 0.6: F(1,2628) = 0.07, p=0.79)

6/6: F(1,2628)=0.63, p=0.43

5/6: F(1,2628)=0.75, p=0.39

4/6: F(1,2628)=0.56, p=0.45

Uninformative: 0/6: F(1,2628) = 7.52, p=0.006

6/6: F(1,2628) = 17.98, p<0.001

5/6: F(1,2628)16.26, p<0.001

4/6: F(1,2628)=5.73, p=0.02

Reliably correct. 0/6: F(1,2628) = 5.61, p=0.02

6/6: F(1,2628)=8.91, p=0.003

5/6: F(1,2628)=8.41, p=0.004

4/6: F(1,2628) =3.47, p=0.06

Third order

Similar others, reliably incorrect vs uninformative. 0/6: F(1,2628)=8.47, p=0.004

6/6: F(1,2628)=13.18, p=0.0003

5/6: F(1,2628)=11.62, p=0.0007

4/6: F(1,2628)=4.16, p=0.04

Similar others, reliably incorrect vs correct. 0/6: $F(1,2628)=14.46$, $p=0.0001$)

6/6: $F(1,2628)=31.09$, $p<0.001$

5/6: $F(1,2628)=32.60$, $p<0.001$

4/6: $F(1,2628) =21.56$, $p<0.001$

Similar others, uninformative vs reliably correct. 0/6: $F(1,2628)=0.16$, $p=0.69$

6/6: $F(1,2628)=1.62$, $p=0.20$

5/6: $F(1,2628)=2.15$, $p=0.14$

4/6: $F(1,2628) = 2.32$, $p=0.13$

Different others, reliably incorrect vs uninformative. 0/6: $F(1,2628)=0.04$, $p=0.84$

6/6: $F(1,2628)=0.29$, $p=0.59$

5/6: $F(1,2628) =0.33$, $p=0.56$

4/6: $F(1,2628)=0.22$, $p=0.64$

Different others, reliably incorrect vs correct. 0/6: $F(1,2628)=0.5$, $p=0.48$.

6/6: $F(1,2628)=2.98$, $p=0.08$

5/6: $F(1,2628)=3.41$, $p=0.06$

4/6: $F(1,2628)=2.56$, $p=0.11$

Different others, uninformative vs reliably correct. 0/6: $F(1,2628)=1.55$, $p=0.21$)

6/6: $F(1,2628)=11.43$, $p=0.0007$

5/6: $F(1,2628)=13.51$, $p=0.0002$

4/6: $F(1,2628)=9.35$, $p=0.002$

Just incorrect signals, game against nature

Second order

Reliably incorrect. 0/6: $F(1,2628)=0.54$, $p=0.46$

6/6: $F(1,2628)=1.40$, $p=0.24$

5/6: $F(1,2628)=1.35$, $p=0.25$

4/6: $F(1,2628)=0.81$, $p=0.37$

Uninformative. 0/6: $F(1,2628)=1.60$, $p=0.21$

6/6: $F(1,2628)=6.98$, $p=0.008$

5/6: $F(1,2628)=8.50$, $p=0.004$

4/6: $F(1,2628)=5.76$, $p=0.02$

Reliably correct. 0/6: $F(1,2628)=0.10$, $p=0.76$

6/6: $F(1,2628)=0.47$, $p=0.49$

5/6: $F(1,2628)=1.36$, $p=0.24$

4/6: $F(1,2628)=2.93$, $p=0.09$

Third order

Similar others, reliably incorrect vs uninformative. 0/6: $F(1,2628)=10.85$, $p=0.001$

6/6: $F(1,2628)=32.17$, $p<0.001$

5/6: $F(1,2628)=33.57$, $p<0.001$

4/6: $F(1,2628)=18.00$, $p<0.001$

Similar others, reliably incorrect vs correct. 0/6: $F(1,2628)=0.37$, $p=0.54$

6/6: $F(1,2628)=9.13$, $p=0.003$

5/6: $F(1,2628)=15.70$, $p<0.001$

4/6: $F(1,2628)=21.79$, $p<0.001$

Similar others, uninformative vs reliably correct: 0/6: $F(1,2628)=0.20$, $p=0.65$

6/6: $F(1,2628)=0.89$, $p=0.34$

5/6: $F(1,2628)=2.60$, $p=0.11$

4/6: $F(1,2628) = 6.78$, $p=0.009$

Different others, reliably incorrect vs uninformative. 0/6: $F(1,2628)=1.37$, $p=0.24$

6/6: $F(1,2628)=3.38$, $p=0.07$

5/6: $F(1,2628)=2.93$, $p=0.09$

4/6: $F(1,2628)=0.70$, $p=0.40$

Different others, reliably incorrect vs correct: 0/6: $F(1,2628)=0.90$, $p=0.34$

6/6: $F(1,2628)=3.96$, $p=0.05$

5/6: $F(1,2628)=5.02$, $p=0.03$

4/6: $F(1,2628)=3.50$, $p=0.06$

Different others, uninformative vs reliably correct. 0/6: $F(1,2628)=0.25$, $p=0.62$

6/6: $F(1,2628)=1.76$, $p=0.18$

5/6: $F(1,2628)=2.49$, $p=0.11$

4/6: $F(1,2628)=2.18$, $p=0.14$

Appendix 11: Linear combinations comparing the social-learners' ability to respond optimally across each level of the social information, for the coordination game.

Note that the bias to respond optimally to reliably-similar signals only exists on trials where the majority of demonstrators chose % and not for 0/6, and so this bias is less pronounced in the coordination game than the game against nature.

<u>All signals, coordination game</u>

<u>Second order</u>

Reliably incorrect. 0/6: F(1,2372)= 0.25, p=0.62

6/6: F(1,2372)=4.59, p=0.03

5/6: F(1,2372)=5.53, p=0.02

4/6: F(1,2372)= 4.74, p=0.03

Uninformative: 0/6: F(1,2372)=3.39, p=0.07

6/6: F(1,2372)=5.28, p=0.02

5/6: F(1,2372)=3.59, p=0.06

4/6: F(1,2372)=0.46, p=0.50

Reliably correct. 0/6: F(1,2372)=0.12, p=0.73

6/6: F(1,2372)=5.77, p=0.02

5/6: F(1,2372) =7.65, p=0.006

4/6: F(1,2372)=7.03, p=0.008

Third order

Similar, reliably incorrect vs uninformative. 0/6: $F(1,2372)=2.17$, $p=0.14$

6/6: $F(1,2372)=5.07$, $p=0.02$

5/6: $F(1,2372)=4.50$, $p=0.03$

4/6: $F(1,2372)=2.23$, $p=0.14$

Similar others, reliably incorrect vs correct. 0/6: $F(1,2372)=0.78$, $p=0.38$.

6/6: $F(1,2372)=10.8$, $p=0.001$.

5/6: $F(1,2372)=13.49$, $p=0.0002$

4/6: $F(1,2372)=10.05$, $p=0.002$

Similar others, uninformative vs reliably correct. 0/6: $F(1,2372)=4.48$, $p=0.03$.

6/6: $F(1,2372)=33.51$, $p<0.001$.

5/6: $F(1,2372)=38.87$, $p<0.001$

4/6: $F(1,2372)=25.12$, $p<0.001$

Different others, reliably incorrect vs uninformative. 0/6: $F(1,2372)=0.04$, $p=0.83$.

6/6: $F(1,2372)=7.87$, $p=0.005$

5/6: $F(1,2372)=10.05$, $p=0.002$

4/6: $F(1,2372)=9.58$, $p=0.002$

Different others, reliably incorrect vs correct. 0/6: $F(1,2372)=0.06$, $p=0.80$

6/6: $F(1,2372)=1.34$, $p=0.24$

5/6: $F(1,2372)=2.01$, $p=0.16$

4/6: $F(1,2372)=2.57$, $p=0.11$

Different others, uninformative vs reliably correct. 0/6: $F(1,2372)=0.21$, $p=0.64$.

6/6: $F(1,2372)=2.58$, $p=0.11$

5/6: $F(1,2372)=2.89$, $p=0.09$

4/6: $F(1,2372)=2.30$, $p=0.13$

Just correct signals, coordination game

Second-order

Reliably incorrect. 0/6: $F(1,2372) = 4.55$, $p=0.03$)

6/6: $F(1,2372) = 0.63$, $p=0.43$

5/6:$F(1,2372)<0.001$, $p=0.99$

4/6: $F(1,2372)=1.77$, $p=0.18$

Uninformative. 0/6: $F(1,2372) = 11.00$, $p=0.0009$

6/6: $F(1,2372) = 15.54$, $p<0.001$

5/6:$F(1,2372)=12.46$, $p=0.0004$

4/6: $F(1,2372)=3.93$, $p=0.05$

Reliably correct: 0/6: $F(1,2372) = 11.15$, $p=0.0009$

6/6: $F(1,2372) = 21.05$, $p<0.001$

5/6: $F(1,2372)=20.27$, $p<0.001$

4/6: $F(1,2372)= 11.51$, $p=0.0007$

Third order

Similar, reliably incorrect vs uninformative. 0/6: $F(1,2372)=20.19$, $p<0.001$

6/6: $F(1,2372)=15.56$, $p<0.001$

5/6; $F(1,2372)=9.77$, $p=0.002$

4/6: $F(1,2372)=0.67$, $p=0.41$

Similar, reliably incorrect vs correct. 0/6: $F(1,2372)=25.06$, $p<0.001$)

6/6: $F(1,2372)=42.73$, $p<0.001$

5/6: $F(1,2372)=41.05$, $p<0.001$

4/6: $F(1,2372)=22.17$, $p<0.001$

Similar, uninformative vs reliably correct. 0/6: $F(1,2372)=0.46$, $p=0.50$

6/6: $F(1,2372)=5.98$, $p=0.01$

5/6: $F(1,2372)=7.44$, $p=0.006$

4/6: $F(1,2372)=7.57$, $p=0.006$

Different, reliably incorrect vs uninformative. 0/6: $F(1,2372)=5.12$, $p=0.03$

6/6: $F(1,2372)=0.27$, $p=0.60$

5/6: $F(1,2372)=0.21$, $p=0.65$

4/6: $F(1,2372)=3.67$, $p=0.06$

Different, reliably incorrect vs correct. 0/6: $F(1,2372)=6.89$, $p=0.009$

6/6: $F(1,2372)=2.24$, $p=0.13$

5/6: $F(1,2372)=0.33$, $p=0.57$

4/6: $F(1,2372)=1.14$, $p=0.29$

Different, uninformative vs reliably correct. 0/6: $F(1,2372)=1.01$, $p=0.32$)

6/6: $F(1,2372)=9.25$, $p=0.002$.

5/6: $F(1,2372)-9.32$, $p=0.002$

4/6: $F(1,2372)=5.78$, $p=0.02$

Just incorrect signals, coordination game

Second order

Reliably incorrect. 0/6: $F(1,2372)=0.24$, $p=0.62$

6/6: $F(1,2372)=0.64$, $p=0.42$

5/6: $F(1,2372)=1.28$, $p=0.26$

4/6: $F(1,2372)=2.07$, $p=0.15$

Uninformative. 0/6: $F(1,2372)=8.65$, $p=0.003$

6/6: $F(1,2372)=29.84$, $p<0.001$

5/6: $F(1,2372)=30.73$, $p<0.001$

4/6: $F(1,2372)=15.77$, $p<0.001$

Reliably correct. 0/6: $F(1,2372)=0$, $p=0.99$

6/6: $F(1,2372)=1.53$, $p=0.22$

5/6: $F(1,2372)=2.19$, $p=0.14$

4/6: $F(1,2372)=2.19$, $p=0.13$

Third order

Similar, reliably incorrect vs uninformative. 0/6: $F(1,2372)=18.65$, $p<0.001$

6/6: $F(1,2372)=49.84$, $p<0.001$

5/6: $F(1,2372)=48.34$, $p<0.001$

4/6: $F(1,2372)=27.29$, $p<0.001$

Similar, reliably incorrect vs correct. 0/6: $F(1,2372)=18.31$, $p<0.001$

6/6: $F(1,2372)=16.64$, $p<0.001$

5/6: $F(1,2372)=13.89$, $p=0.0002$

4/6: $F(1,2372)=6.37$, $p=0.01$

Similar, uninformative vs reliably correct. 0/6: $F(1,2372)=2.96$, $p=0.09$

6/6: $F(1,2372)=0.81$, $p=0.37$

5/6: $F(1,2372)=0.35$, $p=0.56$

4/6: $F(1,2372)=0.01$, $p=0.91$

Different, reliably incorrect vs uninformative. 0/6: $F(1,2372)=0$, $p=0.99$

6/6: $F(1,2372)=1.55$, $p=0.21$

5/6: $F(1,2372)=2.13$, $p=0.14$

4/6: $F(1,2372)=2.31$, $p=0.13$

Different, reliably incorrect vs correct. 0/6: $F(1,2372)=5.90$, $p=0.02$

6/6: $F(1,237)=7.45$, $p=0.006$

5/6: $F(1,2372)=4.26$, $p=0.04$

4/6: $F(1,2372)=0.16$, $p=0.69$

Different, uninformative vs reliably correct. 0/6: $F(1,2372)=5.62$, $p=0.02$

6/6: $F(1,2372)=5.15$, $p=0.02$

5/6: $F(1,2372)=2.24$, $p=0.13$

4/6: $F(1,2372)=0.02$, $p=0.88$

Appendix 12: Logistic regressions predicting whether the social learners chose their social learner optimum during the game against nature in response to correct and incorrect similarity signals.

Predictors included: (i) the centered proportion of demonstrators who chose the demonstrator optimum, (ii) dummies for each combination of similarity and reliability information, minus the omitted category of reliably incorrect- similar signals, and (iii) interactions between each of these dummies and the centered proportion of demonstrators who chose the demonstrator optimum. Robust standard error clustered on social learner.

**Parameter**	**Estimate (game against nature, correct signals)**	**Estimate (game against nature, wrong signals)**
**Intercept**	0.083<br>(0.080)<br>95% CI [-0.07, 0.24]	0.110 .<br>(0.065)<br>95% CI [-0.02, 0.24]
**Centred proportion of demonstrators choosing the demonstrator optimum**	-0.460<br>(0.318)<br>95% CI [-1.08, 0.16]	1.204 ***<br>(0.303)<br>95% CI [0.61, 1.80]
**Reliably incorrect- different dummy [indicates different and is correct with 0.1 probability]**	-0.149<br>(0.524)<br>95% CI [-1.17, 0.88]	-0.021<br>(0.129)<br>95% CI [-0.27, 0.23]
**Uninformative-same dummy [indicates same and is correct with 0.5 probability]**	-0.172<br>(0.212)<br>95% CI [-0.59, 0.24]	-0.032<br>(0.216)<br>95% CI [-0.45, 0.39]
**Uninformative-different dummy [indicates different and is correct with 0.5 probability]**	-0.076<br>(0.183)<br>95% CI [-0.43, 0.28]	0.052<br>(0.212)<br>95% CI [-0.36, 0.47]

**Reliably correct-same dummy [indicates same and is correct with 0.9 probability]**	-0.020<br>(0.166)<br>95% CI [-0.16, 0.40]	-0.956<br>(0.582)<br>95% CI [-1.59, 0.50]
**Reliably correct-different dummy [indicates different and is correct with 0.9 probability]**	0.122<br>(0.144)<br>95% CI [-0.16, 0.40]	-0.134<br>(0.551)<br>95% CI [-1.21, 0.94]
**Centred proportion of demonstrators choosing optimum X reliably incorrect-different dummy**	-1.023<br>(2.037)<br>95% CI [-5.01, 2.96]	-0.541<br>(0.535)<br>95% CI [-1.59, 0.50]
**Centred proportion of demonstrators choosing optimum X uninformative-same dummy**	3.256 ***<br>(0.952)<br>95% CI [1.39, 5.12]	-4.161 ***<br>(0.930)<br>95% CI [-5.98, -2.34]
**Centred proportion of demonstrators choosing optimum X uninformative-different dummy**	-0.241<br>(0.681)<br>95% CI [-1.57, 1.09]	-1.931 *<br>(0.863)<br>95% CI [-3.62, -0.24]
**Centred proportion of demonstrators choosing optimum X reliably correct-same dummy**	4.141 ***<br>(0.875)<br>95% CI [2.43, 5.85]	-4.228<br>(2.825)<br>95% CI [-9.75, 1.30]
**Centred proportion of demonstrators choosing optimum X reliably correct-different dummy**	1.576 *<br>(0.618)<br>95% CI [0.37, 2.79]	-4.154<br>(2.594)<br>95% CI [-9.23, 0.92]

The asterisks denote the level of significance of our p values, with the following key:
**** (p<0.001)*
*** (p<0.01)*
** (p<0.05)*
· (trend: p=0.05 – 0.10 significance)

For blocks that gave incorrect similarity information, the social learners were more likely to respond optimally as more demonstrators did (centred proportion of demonstrators choosing the demonstrator optimum effect). This effect reflected the omitted category of reliably incorrect - similar dummy. As a reliably incorrect signal of similarity was only correct with a probability of 0.1, then smart social learners should have treated this signal as if it were always incorrect. Reliably incorrect-similar signals can be flipped to understand that

the learners were more likely to be playing a different game version to the demonstrators. Indeed, the social learners followed the minority of the demonstrators under both reliably incorrect-similar and reliably correct-different signals (Figures 5A and 5F), suggesting that they understood this complexity. Of course, the strategy of following the minority on reliably incorrect-similar blocks would only work if the similarity signal was in fact incorrect. The probabilities implemented meant that the social learners would have played the same game version as the demonstrators on approximately 10% of blocks with these reliably incorrect-similar blocks. In this case, following the minority would have made the learners answer sub-optimally.

When the blocks gave incorrect similarity signals, the social learners were less likely to choose their social learner optimum as more demonstrators did for blocks with uninformative-similar and uninformative-different signals. The learners did not treat an uninformative signal as if it were always incorrect. As the uninformative signal rendered the similarity information arbitrary, we expected the social learners just to choose the strategy that they preferred on these blocks. Figure 5 reveals that the social learners responded at chance towards the uninformative-different signals (Figure 5E), suggesting that they understood these signals to be arbitrary. Interestingly, the social learners instead copied the majority around uninformative-similar others (Figure 5B). This strategy led to the social learners answering sub-optimally whenever this signal of similarity happened to be incorrect. Taken together, this pattern may imply that some social learners arbitrarily committed to a strategy of copying similar others– even if they had uninformative signals– to improve their chances of answering optimally on the off-chance that the uninformative signal happened to be correct. That is, they may have followed a social-learning rule to ‘copy similar others’.

When the blocks gave correct similarity signals (middle column of Table 4), the social learners were significantly more likely to answer optimally as more demonstrators chose their

demonstrator optimum for the following signals: uninformative-similar, reliably correct-similar, and reliably correct-different. The social learners treated these signals as if they were always correct, which allowed them to respond optimally to these signals when the blocks did provide correct similarity information. This strategy made sense for the reliably correct signals as, based on the probabilities implemented in-game, a reliably correct signal of gave correct information about similarity or difference on approximately 90% of the blocks. The social learners followed the majority on uninformative-similar signals (Figure 5B), though this strategy only allowed the learners to answer optimally on approximately half the blocks when uninformative signals happen to give correct similarity information.

Appendix 13: Logistic regressions predicting whether the social learners chose their social learner optimum during the coordination game in response to correct and incorrect similarity signals.

Predictors included: (i) the centered proportion of demonstrators who chose the demonstrator optimum, (ii) dummies for each combination of similarity and reliability information, minus the omitted category of reliably incorrect- similar signals, and (iii) interactions between each of these dummies and the centered proportion of demonstrators who chose the demonstrator optimum. Robust standard error clustered on social learner.

Parameter	Estimate (coordination game, correct signals, full data)	Estimate (coordination game, incorrect signals, full data)
**Intercept**	0.127 .<br>(0.067)<br>95% CI [0, 0.26]	0.016 .<br>(0.065)<br>95% CI [0-0.11, 0.14]
**Centred proportion of demonstrators choosing the demonstrator optimum**	0.008<br>(0.224)<br>95% CI [-0.43, 0.45]	1.603 ***<br>(0.288)<br>95% CI [1.04, 2.17]
**Reliably incorrect-different dummy [indicates different and is correct with 0.1 probability]**	1.389 *<br>(0.674)<br>95% CI [0.07, 2.71]	0.179<br>(0.146)<br>95% CI [-0.11, 0.46]
**Uninformative-same dummy [indicates same and is correct with 0.5 probability]**	-0.497 *<br>(0.205)<br>95% CI [-0.90, -0.10]	-0.137<br>(0.200)<br>95% CI [-0.53, 0.26]
**Uninformative-different dummy [indicates different and is correct with 0.5 probability]**	-0.306 .<br>(0.184)<br>95% CI [-0.67, 0.05]	-0.041<br>(0.198)<br>95% CI [-0.43, 0.35]
**Reliably correct-same dummy [indicates same and is correct with 0.9 probability]**	-0.129<br>(0.183)	0.309<br>(0.292)<br>95% CI [-0.26, 0.88]

	95% CI [-0.49, 0.23]	
**Reliably correct-different dummy [indicates different and is correct with 0.9 probability]**	-0.109 (0.137) 95% CI [-0.38, 0.16]	0.964 (0.672) 95% CI [-0.35, 2.28]
**Centred proportion of demonstrators choosing optimum X reliably incorrect-different dummy**	-4.14 . (2.217) 95% CI [-8.48, 0.19]	0.010 (0.525) 95% CI [-1.03, 1.03]
**Centred proportion of demonstrators choosing optimum X uninformative-same dummy**	3.894 *** (0.833) 95% CI [2.26, 5.52]	-4.925 *** (0.831) 95% CI [-6.55, -3.30]
**Centred proportion of demonstrators choosing optimum X uninformative-different dummy**	0.138 (0.682) 95% CI [-1.19, 1.47]	-0.437 (0.853) 95% CI [-2.11, 1.23]
**Centred proportion of demonstrators choosing optimum X reliably correct-same dummy**	5.575 *** (0.936) 95% CI [3.75, 7.40]	-7.315 *** (1.651) 95% CI [-10.54, -4.09]
**Centred proportion of demonstrators choosing optimum X reliably correct-different dummy**	1.413 ** (0.548) 95% CI [0.34, 2.48]	-5.983 ** (2.147) 95% CI [-10.18, -1.78]

The asterisks denote the level of significance of our p values, with the following key:
**** (p<0.001)*
*** (p<0.01)*
** (p<0.05)*
· (trend: p=0.05 – 0.10 significance)

When the blocks gave incorrect similarity information, then the social learners were more likely to coordinate on the social learner optimum as more demonstrators did (Centred proportion of demonstrators choosing the demonstrator optimum effect). This effect reflected the omitted category of reliably incorrect- similar dummy. This effect shows that the social learners responded to reliably incorrect signals of similarity as if these were always incorrect. This makes sense as the probabilities implemented in-game meant that reliably incorrect signals gave false information on approximately 90% of blocks.

When the blocks gave incorrect similarity information, the social learners were less likely to coordinate on their optimal option as more demonstrators coordinated on the demonstrator optimum for the following signals: uninformative-similar, reliably correct-similar and reliably-correct different. Interestingly, when the blocks gave correct social information (middle column Table 5), then the social learners were more likely to coordinate on the social learner optimum as more demonstrators did for the same dummies: uninformative-similar, reliably correct-similar, and reliably correct-different. Taking these results together, the social learners treated these signals as if they were always correct. This led them to answer optimally whenever the blocks happened to provide correct similarity information, though led the social learner to answer sub-optimally whenever the block gave incorrect similarity information. Treating the reliably correct signals as if they are always correct makes sense as the probabilities implemented meant that these blocks gave correct signals of similarity or difference approximately 90% of the time. The social learners also responded to an uninformative signal of similarity as if this signal were always correct, though uninformative signals only provided correct similarity information on approximately half the blocks.

Appendix 14: The scatterplots, showing that the participant IDs often change as a function of the signal they see.

This is to show individual level variation in each of the learner's chosen strategies. As these IDs are hard to see, we also repeat this analysis as a heatmap in appendix 15.

Appendix 14i) Game against nature, reliably correct

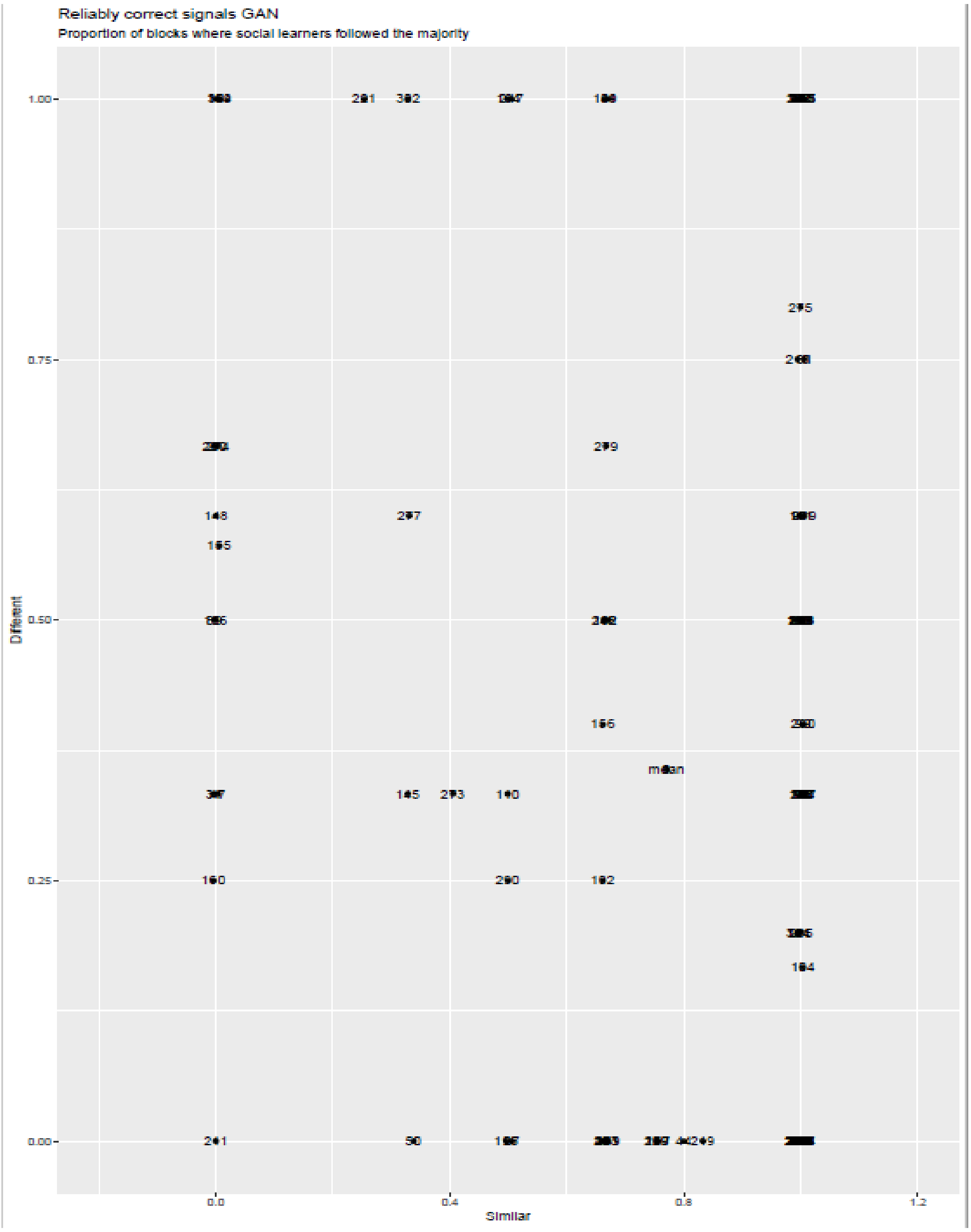

Appendix 14ii) Game against nature, reliably incorrect

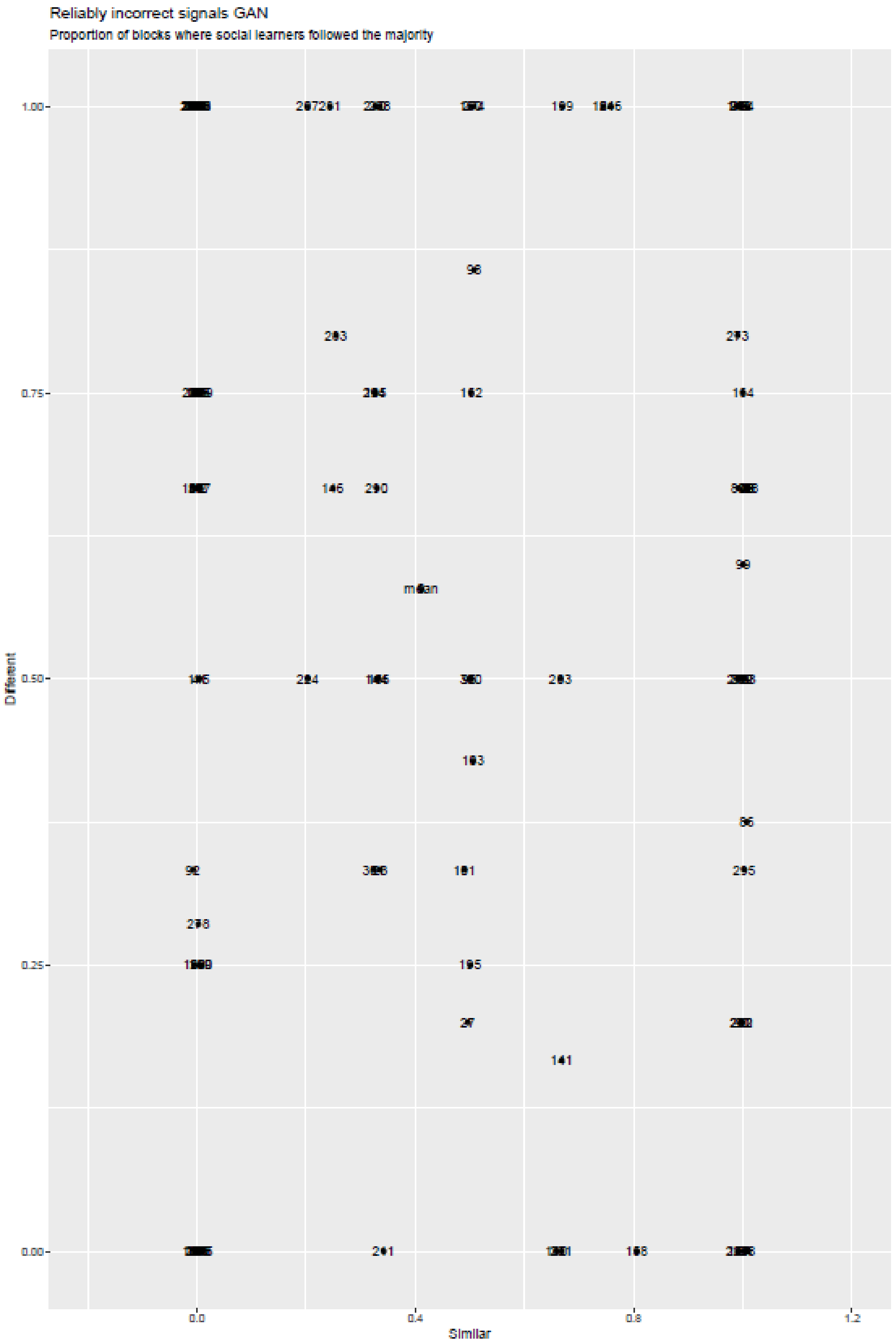

Appendix 14iii) Coordination game, reliably correct

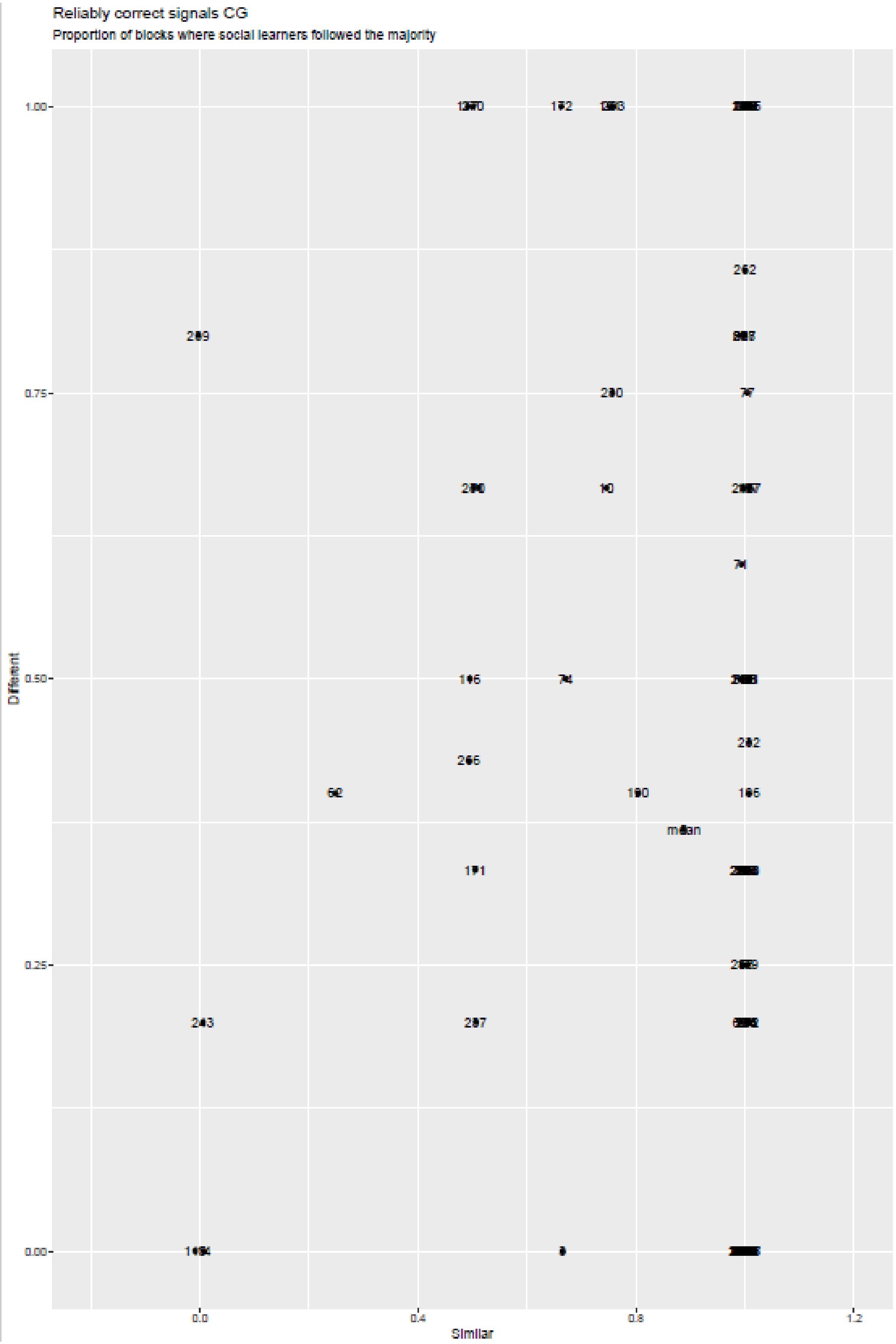

Appendix 14iv) Coordination game, reliably incorrect

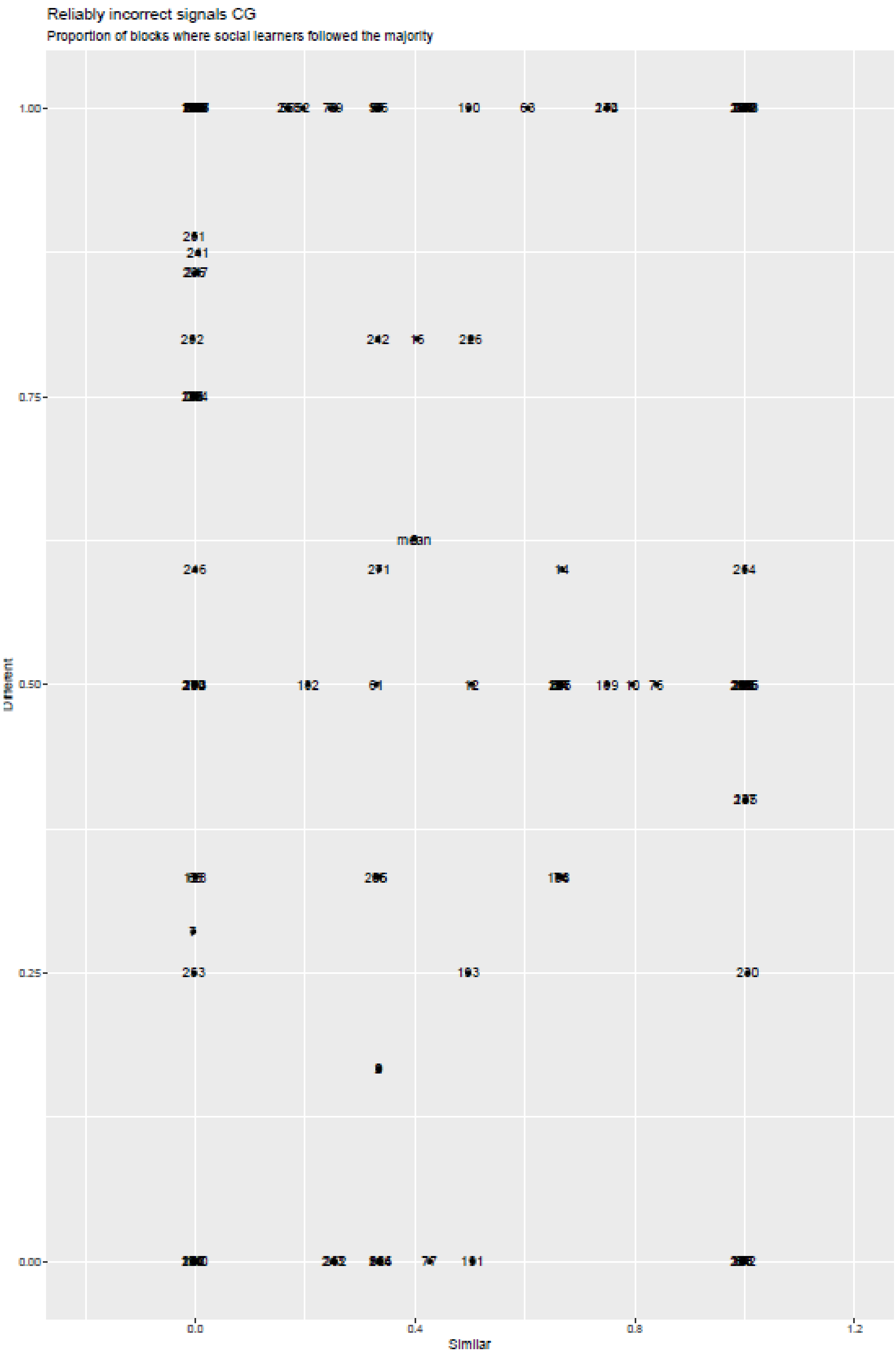

Appendix 15: The heatmaps show the social learner's chosen strategies as a proportion of blocks where they follow the majority of similar others (on the x axis) by the proportion of blocks where they follow the majority of different others (on the y axis). This is to show individual level variation in each of the learner's chosen strategies. The x axis gives 0-1 proportion of all social learners who followed the majority of groups identified as similar, while the y axis gives the 0-1 proportion of all social learners who followed the majority of groups identified as different. The darkness of the grey indicates frequency, with darker colours denoting a denser patch. Any white areas show that none of the learners employed this strategy combination. Finally, the red square denotes the social learning strategy averaged over all participants.

Appendix 15i) Game against nature, reliably correct

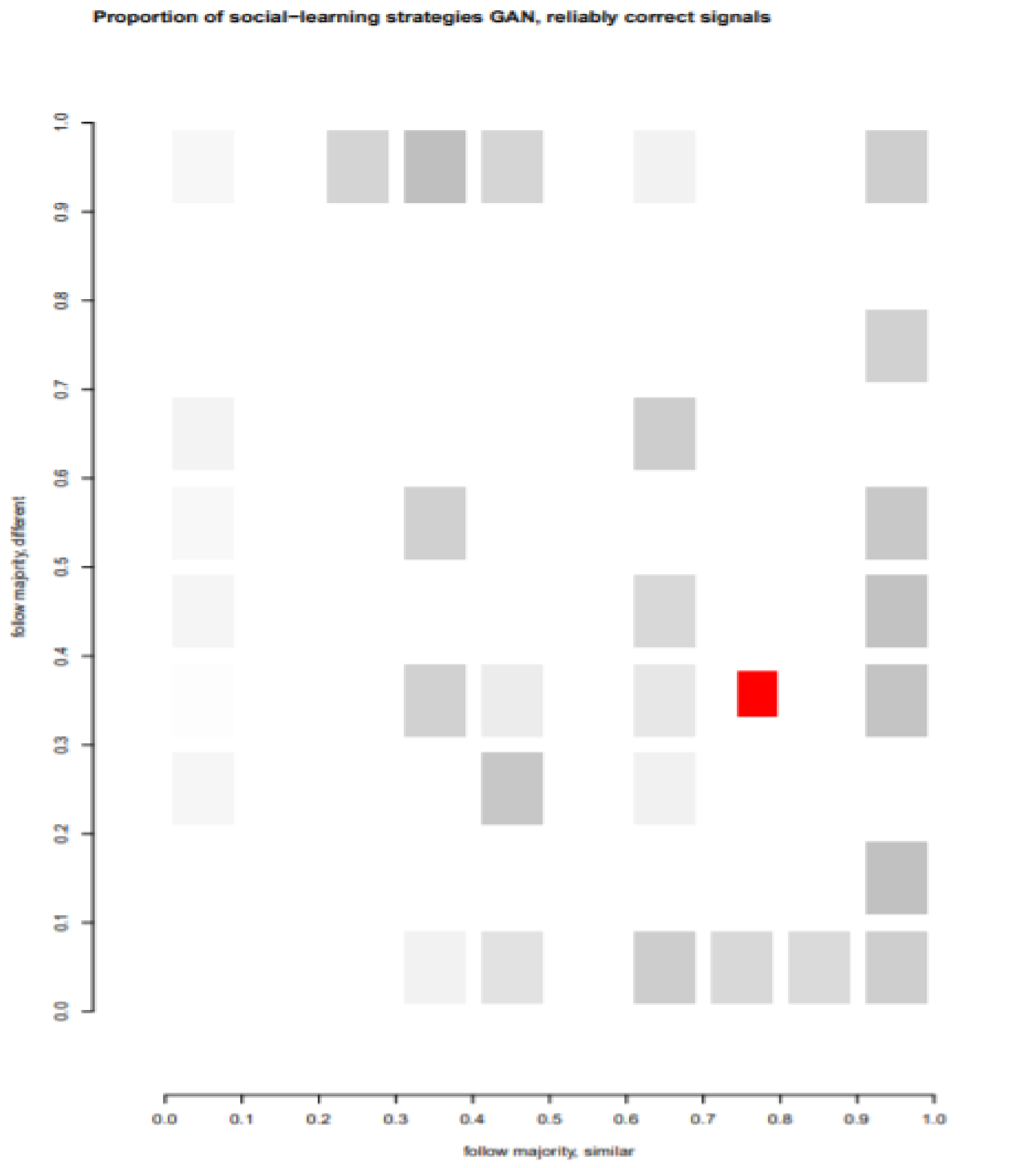

Note that there is a cluster along the top (i.e., these learners always follow the majority of different others, but are less sure when it comes to responding to similar others). There is also a cluster at the side (i.e., these learners always follow the majority of similar others, but they are less sure when it comes to different others). There is also a cluster along the bottom (i.e., these people copy the minority of different others but seem less sure for similar others). There are also some less differentiated strategies in the middle. The average

social learning strategy is to copy the majority of similar others with a probability of ~0.7, and to follow the majority of different others with a probability of ~0.3. This perhaps makes sense: in order to choose their optimum, the social learners should copy the majority of similar others and the minority on different others, and so the average strategy is a part adjustment to this. Note that the optimal strategy is given by the bottom right square: an optimal learning strategy is to always copy the majority of reliably similar others but copy the minority of reliably different others. This square is shaded in fairly darkly, suggesting that a few social learners manage to answer optimally.

Appendix 15ii) Game against nature, reliably incorrect

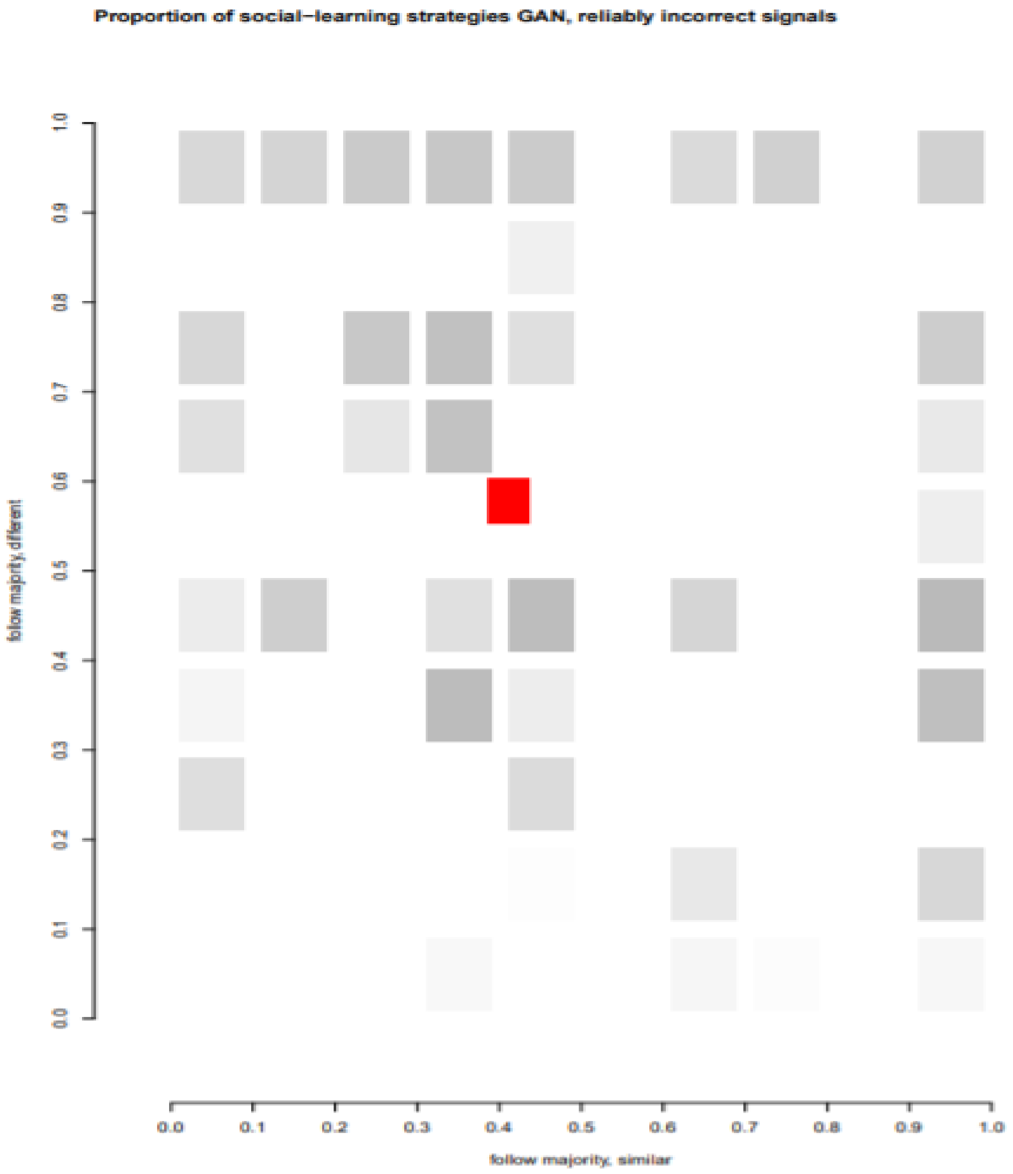

There is definitely a cluster at the top (who always copy the majority of different others with a reliably incorrect signals) and a cluster at the right-hand side (who always copy the majority of similar others with reliably incorrect signals). There also seems to be a cluster at the side, who copy the minority of similar others but are unsure for different others. The optimal square in this case is the top left (always follow the minority of similar others, but majority of different others, with reliably incorrect signals). This is filled in and so some do answer optimally. However, there is a broader range of strategies for the reliably incorrect

signals and so the average strategy cancels out at chance and perhaps gives the impression that learners adjust to the reliably incorrect signal less than they actually do.

Appendix 15iii) Coordination game, reliably correct

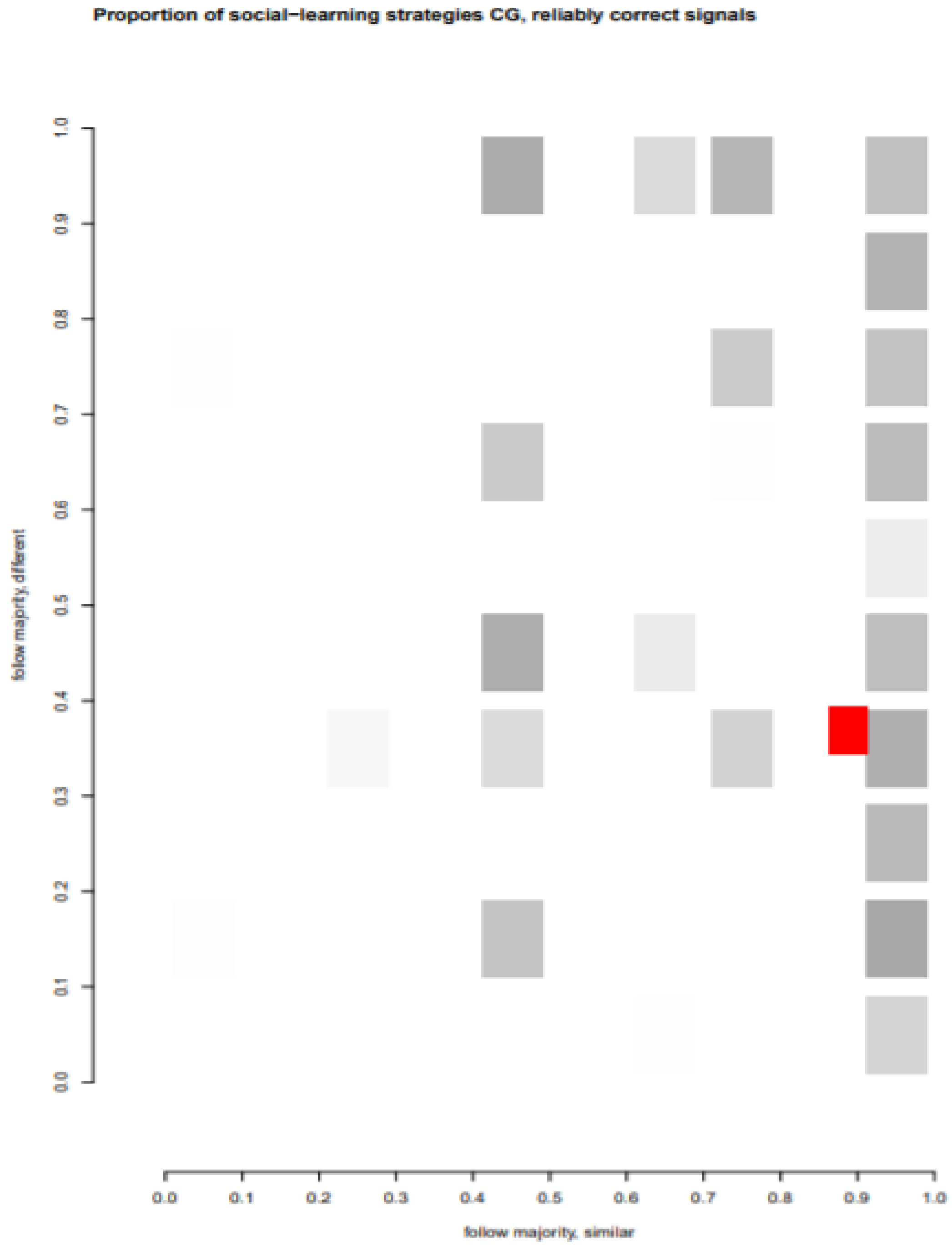

This graph is perhaps the least spread out. The optimal strategy for a reliably correct signal is to follow the majority of similar others but the minority of different others. We find

that this is shaded and so some do answer optimally. There is a cluster along the right-hand side: some learners always copy the majority of similar others but show more variation in the response to different others. There is also a small cluster in the top right: some participants always copy the majority of reliably different others, and trend towards following the majority of similar others. There is also a strip at around 0.4 of following the majority of similar others, where the participants respond differing to the different signal. Although the participants may seemingly respond anywhere to reliably different others, note that all learners avoid following the minority of similar others. This is why the left side of the graph is blank. Thus, following the minority may only be used on a coordination game in response to reliably correct signals of difference or reliably incorrect signals of similarity.

Appendix 15iv) Coordination game, reliably incorrect

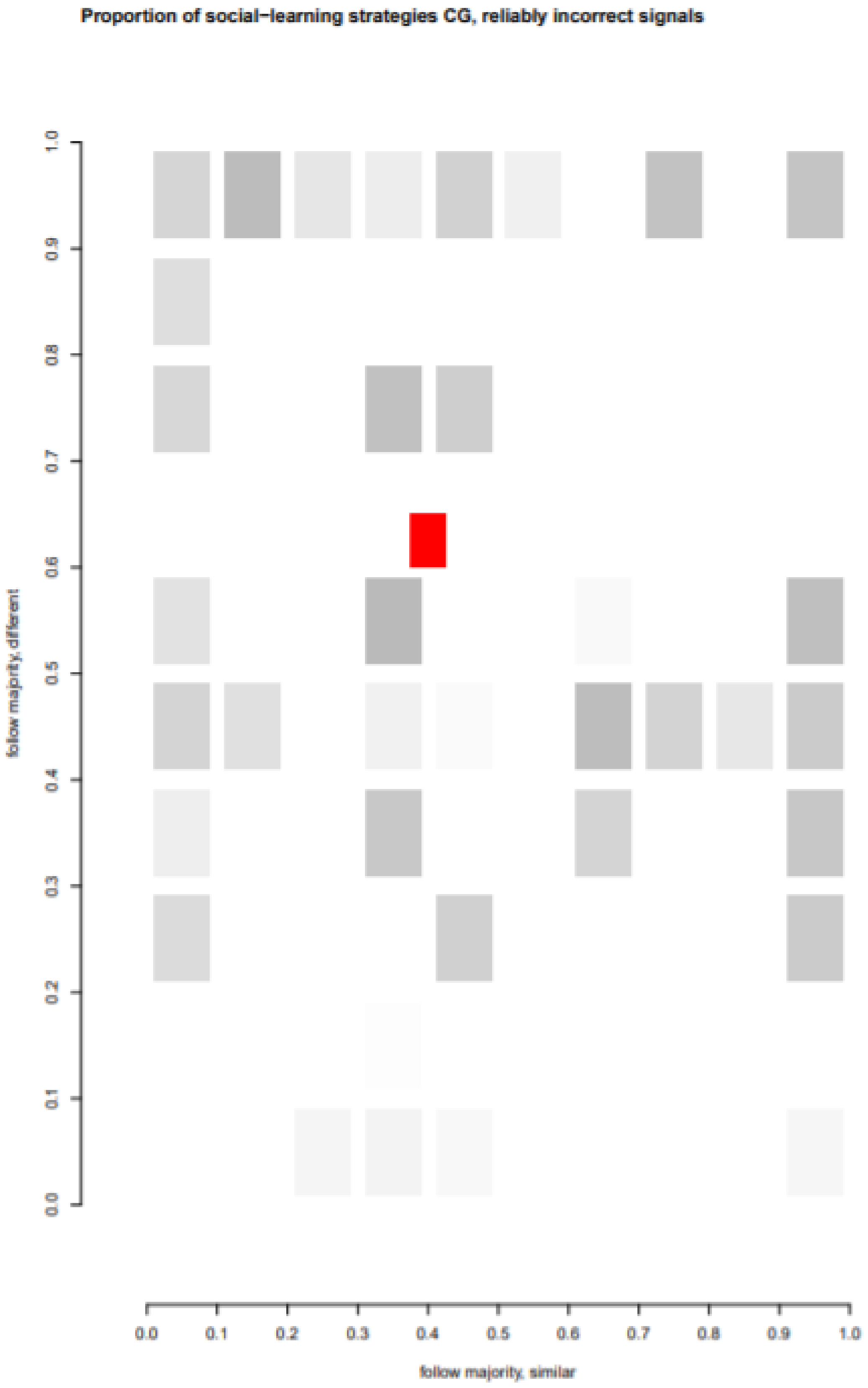

The responses to reliably incorrect signals for the coordination game is perhaps the most varied. The optimal solution is to follow the minority of similar others and the majority of different others for a reliably incorrect signal (given by the top left square). As we can see, some of the learners do show optimal strategies. More broadly, we have three edges: some participants copy the majority of similar others, but there is more noise for different others; a cluster at the top where some always copy the majority of different others but are less differentiated for similar others and a cluster on the left where some participants follow the minority of similar others but are less differentiated for different others with reliably incorrect signals. There is then a range of adjustments in the middle.

It may be tempting to conclude that those participants in the middle are ignoring social information and answering optimally. However, we asked the participants to (anonymously) report whether they actually used the social information. Those that denied using social information are not necessarily those in the middle. Some participants take an undifferentiated strategy in response to three orders of social information on purpose.
