# Supplementary material for: What is the extent of a frequency-dependent social learning strategy space?
Source: Evol Hum Sci. 2022 Apr 13;4:e13. doi: 10.1017/ehs.2022.11 (PMC10426114; doi:10.1017/ehs.2022.11)
Supplement: Supplementary file 1 [file S2513843X22000111sup.zip › S2513843X22000111sup001.docx]

**Supplementary materials for ‘****What is the extent of a frequency-dependent social learning strategy space?’**

Note that the following materials represent the supplementary text for the ‘*What is the extent of a frequency-dependent social learning strategy space?*’ paper. In this document, we investigate how the social information presented in-game and the participant demographics affected our participants’ self-reported social-learning preferences (S1). We then repeat key analyses from our study (S2). One experimental session of the coordination game crashed before the participants could provide demographics. All analyses reported in-text use the full data, but the data in S2 aims to repeat the coordination game analyses with the data from this crashed session removed for transparency purposes.

**S1: The analysis of the participants’ self-reported social -learning strategies.**

**S1A: A screenshot of the end-survey shown to the participants at the end of each experimental session**.
The first 2 boxes on the left ask the social-learner how they used social information. The box on the top right confirms that the participants understood the reliability signal, and the box in the bottom right asks for demographics (note that the demonstrators only saw this bottom right box).

Note that, for the coordination game, only 116 participants completed this survey due to a crash in the final session. The following regressions predicting whether the social learners’ self-reported a preference to follow the majority during the coordination game therefore only contain the data for these 116 participants.


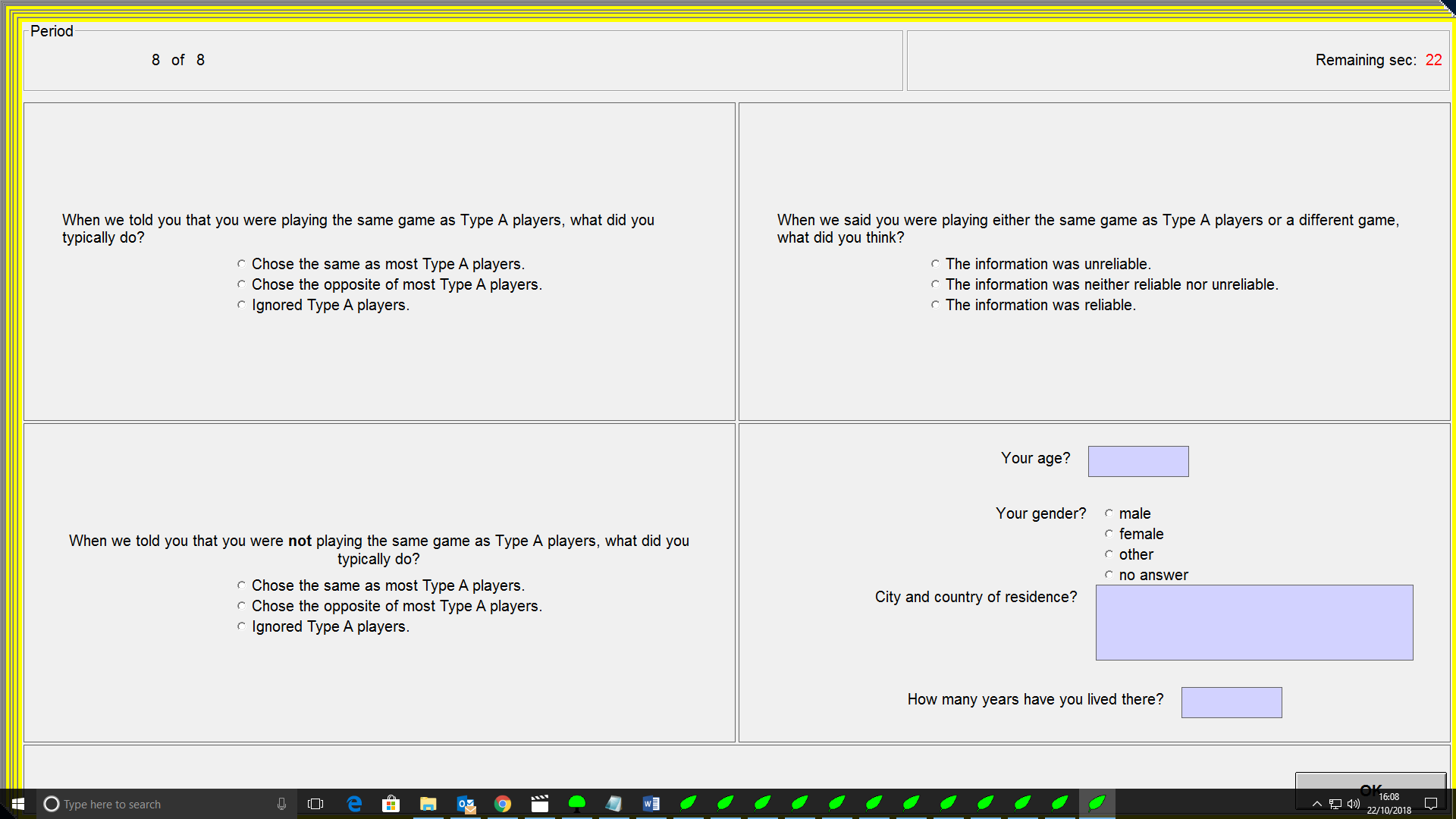


**S1B: These logistic regressions predict whether the social learners self-reported using the social information or not.**
The predictors were: (i) the centered proportion of demonstrators choosing % on each block; (ii) each combination of similarity and reliability signals minus the omitted category of reliably incorrect-similar signals and (iii) interactions between each of these signals and the centered proportion of demonstrators who chose %. Robust standard errors clustered on social learner.

For the game against nature, the social learners reported being more likely to use social information as more demonstrators chose %. They were however less likely to use social information as more demonstrators chose % for the following signals: reliably incorrect and reliably correct signals of difference. This is interesting, as these signals both provide meaningful social information but in different ways. Perhaps this explains the less pronounced response to the reliability signals for different others that was reported in-text for the game against nature: it seems that the social learners as a whole were less likely to use social information from different others.

For the coordination game, none of the signals significantly predict whether the social learners self-reported using social information. There is a significant intercept which may suggest that there is some variation in who uses social information that the signals alone cannot explain.

| Parameter | Estimate (game against nature, no controls) | Estimate (coordination game, crash removed) |
| --- | --- | --- |
| Intercept | 0.88 *** (0.22) 95% CI [0.46-1.31] | 0.89 *** (0.24) 95% CI [-0.42 1.36] |
| Centred proportion of demonstrators choosing % | 1.09 ** (0.42)  95% CI [0.26-1.92] | 0.58 (0.56) 95% CI [-0.51 -1.67] |
| Reliably incorrect-different dummy [signal indicates different and is correct with 0.1 probability] | 0.04 (0.17) 95% CI [-0.30- 0.38] | -0.21 (0.15) 95% CI [-0.51-0.08] |
| Uninformative -same dummy [signal indicates same and is correct with 0.5 probability] | -0.26 (0.14)  95% CI [-0.54-0.02] | 0.09 (0.19) 95% CI [-0.29-0.47] |
| Uninformative -different dummy [signal indicates different and is correct with 0.5 probability] | -0.07  (0.18) 95% CI [-0.41 - 0.28] | 0.24 (0.21) 95% CI [-0.17-0.64] |
| Reliably correct-same dummy [signal indicates same and is correct with 0.9 probability] | 0.04 (0.14)  95% CI [-0.23 -0.32] | 0.08 (0.20) 95% CI [-0.17-0.64] |
| Reliably correct -different dummy [signal indicates different and is correct with 0.9 probability] | -0.16  (0.20)  95% CI [-0.54- 0.22] | 0.04 (0.20) 95% CI [-0.35-0.42] |
| Centred proportion of demonstrators choosing % X reliably incorrect-different dummy | -1.26 * (0.52)  95% CI [-2.27- -0.25] | -0.24 (0.57)  95% CI [-1.35-0.87] |
| Centred proportion of demonstrators choosing % X uninformative-same dummy | -1.14 . (0.59)  95% CI [-2.30-0.02] | <0.001 (0.63)  95% CI [-1.22-1.23] |
| Centred proportion of demonstrators choosing % X uninformative-different dummy | -1.03  (0.54)  95% CI [-2.08 - 0.02] | -0.23 (0.72) 95% CI [-1.64-1.19] |
| Centred proportion of demonstrators choosing % X reliably correct-same dummy | -1.16  (0.63)  95% CI [-2.39 -0.08] | -0.79 (0.53)  95% CI [-1.83-0.26] |
| Centred proportion of demonstrators choosing % X reliably correct-different dummy | -1.21 * (0.55)  95% CI [-2.30- -0.12] | 0.45 (0.66) 95% CI [-0.84-1.74] |

*The asterisks denote the level of significance of our p values, with the following key:
*** (p<0.001)
** (p<0.01)
* (p<0.05)****^.^*** *(trend: p=0.05 – 0.10 significance)*

**S1C:** **The logistic regression modelling whether social learners self-reported using social information, with controls.**
The predictors were: (i) the centered proportion of demonstrators choosing % on each block; (ii) each combination of similarity and reliability signals minus the omitted category of reliably incorrect-similar signals and (iii) interactions between each of these signals and the centered proportion of demonstrators who chose % and (iv) key demographics and other controls. Robust standard errors clustered on social learner.

Interestingly, adding in the control variables meant that the social learners for the game against nature are now significantly less likely to use social information under the following signals: uninformative signals from similar and different others. Taken together from the betas above, this shows that the social learners do not use social information from different others, or for uninformative signals, when learning a game against nature. Perhaps reliably correct and reliably incorrect signals of similarity are all the learners try to account for. The social learners who reported being from India also reported being more likely to use social information during the game against nature only, in comparison to the international students from other countries.

Finally, we note that the social learners under both the game against nature and the coordination game are significantly less likely to use social information as the blocks progressed, suggesting that social information may be up-weighted when the task still feels novel and uncertain. For the coordination game, there are no other significant control predictors except being less likely to use social information as the blocks progressed.

| Parameter | Estimate (game against nature, with controls) | Estimate (coordination game, with controls) |
| --- | --- | --- |
| Intercept | 1.52 (1.52) 95% CI [-1.45-4.50] | -0.41 (1.88) 95% CI [-4.09-3.27] |
| Centred proportion of demonstrators choosing % | 1.09 * (0.46) 95% CI [0.19-1.99] | 0.57 (0.57) 95% CI [-0.54-1.69] |
| Reliably incorrect-different dummy [signal indicates different and is correct with 0.1 probability] | 0.05  (0.17) 95% CI [-0.28- 0.39] | -0.23 (0.15) 95% CI [-0.52-0.05] |
| Uninformative-same dummy [signal indicates same and is correct with 0.5 probability] | -0.26 (0.15) 95% CI [-0.55-0.03] | 0.10 (0.18) 95% CI [-0.25-0.45] |
| Uninformative-different dummy [signal indicates different and is correct with 0.5 probability] | -0.07  (0.18) 95% CI [-0.41- 0.28] | 0.22 (0.19) 95% CI [-0.16-0.60] |
| Reliably correct-similar dummy [signal indicates same and is correct with 0.9 probability] | 0.06 (0.14)  95% CI [-0.22 - 0.33] | 0.07 (0.20) 95% CI [-0.33 -0.41] |
| Reliably correct-different dummy [signal indicates different and is correct with 0.9 probability] | -0.15 (0.20)  95% CI [-0.53- 0.24] | 0.04 (0.19) 95% CI [-0.33 -0.41] |
| Centred proportion of demonstrators choosing % X reliably incorrect-different dummy | -1.22 * (0.59)  95% CI [-2.37- -0.06] | -0.44 (0.57) 95% CI [-1.55- 0.67] |
| Centred proportion of demonstrators choosing % X uninformative-same dummy | -1.23 * (0.61)  95% CI [-2.44--0.03] | -0.28 (0.63) 95% CI [-1.51-0.95] |
| Centred proportion of demonstrators choosing % X uninformative-different dummy | -1.20 * (0.54)  95% CI [-2.25 - -0.15] | -0.37 (0.74) 95% CI [-1.82- 1.08] |
| Centred proportion of demonstrators choosing % X reliably correct-same dummy | -1.07 (0.69)  95% CI [-2.44-0.29] | -1.13 (0.59) 95% CI [-2.28-0.03] |
| Centred proportion of demonstrators choosing % X reliably correct-different dummy | -1.28 * (0.60) 95% CI [- 2.45 - -0.12] | 0.27 (0.63) 95% CI -0.95-1.50] |
| Percentage as optimal dummy [signal indicates percentage is optimal option] | -0.04  (0.08) 95% CI [-0.20 -0.12] | 0.02 (0.11) 95% CI [-0.20-0.23] |
| Age | 0.01 (0.07) 95% CI [-0.13 -0.14] | 0.05 (0.08) 95% CI [-0.11 -0.22] |
| Gender | -0.66 (0.39) 95% CI [-1.42-0.10] | -0.17 (0.42) 95% CI [-0.99-0.66] |
| Time in residence | -0.01 (0.03) 95% CI [-0.06-0.04] | 0.04 (0.02) 95% CI [0.00 0.09] |
| Block Index | -0.01 *** (<0.001) 95% CI [-0.20- -0.12] | -0.02 *** (<0.001)  95% CI [-0.03- -0.01] |
| India dummy | 0.529 ** (0.164)  95% CI [0.21-0.85] | - |

*The asterisks denote the level of significance of our p values, with the following key:
*** (p<0.001)
** (p<0.01)
* (p<0.05)****^.^*** *(trend: p=0.05 – 0.10 significance)*

**S1D: The logistic regression modelling whether social learners self-reported following the minority or majority.**
The predictors were: (i) the centered proportion of demonstrators choosing % on each block; (ii) each combination of similarity and reliability signals minus the omitted category of reliably incorrect-similar signals and (iii) interactions between each of these signals and the centered proportion of demonstrators who chose %. Robust standard errors clustered on social learner.

During the game against nature (left-hand column, Table 1B), the social learner self-reported being less likely to follow the majority on the following blocks: reliably incorrect-different, uninformative-different, and reliably correct-different. The social learner seemingly treated all signals from different others as if they were reliably correct as they reported following the minority on all of these blocks. The social learners became more likely to choose % as the demonstrators did under the following signals: reliably correct-similar and reliably incorrect-different. These self-reported strategies matched those given in table 2 in-text and shows a complex processing of social information on conditions where the social learner aimed to follow the majority. Taken together, the social learners’ self-reported use of learning strategies suggests that they understood the complex social information and were trying to extract optimal social information on these blocks, though that they did not pay as much attention to the reliability signal given alongside different others.

However, there were no significant predictors for the coordination game (right-hand column, Table B).

| Parameter | Estimate (game against nature, no controls) | Estimate (coordination game, crash removed) |
| --- | --- | --- |
| Intercept | 0.702 *** (0.195) 95% CI [0.32-1.08] | 0.123 (0.168) 95% CI [-0.21 -0.45] |
| Centred proportion of demonstrators choosing % | -0.760 (0.622)  95% CI [-1.97-0.45] | 0.518 (0.457) 95% CI [-0.37 -1.41] |
| Reliably incorrect-different dummy [signal indicates different and is correct with 0.1 probability] | -0.767 ** (0.243) 95% CI [-1.24- -0.29] | 0.014 (0.178) 95% CI [-0.33-0.36] |
| Uninformative -same dummy [signal indicates same and is correct with 0.5 probability] | 0.292 (0.191)  95% CI [-0.08-0.67] | 0.022 (0.231) 95% CI [-0.07-0.82] |
| Uninformative -different dummy [signal indicates different and is correct with 0.5 probability] | -0.711 ** (0.269) 95% CI [-1.24 - -0.19] | 0.376 . (0.228) 95% CI [-0.07-0.82] |
| Reliably correct-same dummy [signal indicates same and is correct with 0.9 probability] | -0.137 (0.160)  95% CI [-0.45 -0.18] | 0.255 (0.229) 95% CI [-0.19-0.70] |
| Reliably correct -different dummy [signal indicates different and is correct with 0.9 probability] | -0.893 *** (0.252)  95% CI [-1.39- -0.40] | 0.194 (0.171) 95% CI [-0.14-0.53] |
| Centred proportion of demonstrators choosing % X reliably incorrect-different dummy | 1.389 * (0.684)  95% CI [0.05-2.72] | -0.129 (0.612)  95% CI [-1.32-1.07] |
| Centred proportion of demonstrators choosing % X uninformative-same dummy | 1.456 . (0.776)  95% CI [-0.06-2.97] | -0.583 (0.682)  95% CI [-1.92-0.75] |
| Centred proportion of demonstrators choosing % X uninformative-different dummy | 1.058  (0.785)  95% CI [-0.48 - 2.59] | -1.171 . (0.676) 95% CI [-2.49-0.15] |
| Centred proportion of demonstrators choosing % X reliably correct-same dummy | 1.492 * (0.704)  95% CI [0.12-2.87] | 0.126 (0.686)  95% CI [-1.21-1.46] |
| Centred proportion of demonstrators choosing % X reliably correct-different dummy | 0.910 (0.771)  95% CI [-0.60-2.42] | -0.841 (0.565) 95% CI [-1.94-0.26] |

*The asterisks denote the level of significance of our p values, with the following key:
*** (p<0.001)
** (p<0.01)
* (p<0.05)****^.^*** *(trend: p=0.05 – 0.10 significance)*

**S1E: The logistic regression modelling whether social learners self-reported following the minority or majority, with controls.**
The predictors were: (i) the centered proportion of demonstrators choosing % on each block; (ii) each combination of similarity and reliability signals minus the omitted category of reliably incorrect-similar signals and (iii) interactions between each of these signals and the centered proportion of demonstrators who chose % and (iv) key demographics and other controls. Robust standard errors clustered on social learner.

During the game against nature (left-hand column, Table 1C), the social learners report being less likely to follow the majority in response to reliably incorrect-different, uninformative-different and reliably correct-different (and so choose the opposite option to any group of different others). The social learners also report being more likely to follow the majority as more demonstrators chose % under both a reliably correct-similar and a reliably incorrect-different signal, matching the significant betas in the regression above. The social learners were less likely to follow the majority as the blocks progressed (Block Index dummy) Finally, those from India self-report being more likely to follow the majority (India dummy beta).

During the coordination game (right-hand column, Table 1C), the social learners report being less likely to follow the majority as more demonstrators choose % under both uninformative-similar and uninformative-different blocks. The only significant control predictor is that the learners reported being less likely to follow the majority as the block index increased (Block Index beta). Taken together, this confirms that the social learners reported being less likely to conform as the blocks progressed, perhaps because they felt more confident in their decisions as the blocks progressed.

| Parameter | Estimate (game against nature, with controls) | Estimate (coordination game, with controls) |
| --- | --- | --- |
| Intercept | 1.292 . (0.749) 95% CI [-0.17-2.75] | -0.417 (0.957) 95% CI [-2.28-1.45] |
| Centred proportion of demonstrators choosing % | -1.187 . (0.651) 95% CI [-2.46-0.08] | 0.627 (0.451) 95% CI [-0.25-1.51] |
| Reliably incorrect-different dummy [signal indicates different and is correct with 0.1 probability] | -0.822 *** (0.246) 95% CI [-1.30- -0.34] | 0.054 (0.181) 95% CI [-0.30-0.41] |
| Uninformative-same dummy [signal indicates same and is correct with 0.5 probability] | 0.295 (0.187) 95% CI [-0.07-0.66] | 0.083 (0.233) 95% CI [-0.37-0.54] |
| Uninformative-different dummy [signal indicates different and is correct with 0.5 probability] | -0.759 ** (0.269) 95% CI [-1.28- -0.24] | 0.353 (0.220) 95% CI [-0.18-0.73] |
| Reliably correct-similar dummy [signal indicates same and is correct with 0.9 probability] | -0.181 (0.164)  95% CI [-0.50 - 0.14] | 0.273 (0.233) 95% CI [-0.18 -0.73] |
| Reliably correct-different dummy [signal indicates different and is correct with 0.9 probability] | -0.934 *** (0.250)  95% CI [-1.42- -0.45] | 0.188 (0.173) 95% CI [-0.15 -0.52] |
| Centred proportion of demonstrators choosing % X reliably incorrect-different dummy | 1.604 * (0.722)  95% CI [0.20-3.01] | -0.489 (0.608) 95% CI [-1.67- 0.70] |
| Centred proportion of demonstrators choosing % X uninformative-same dummy | 1.563 * (0.790)  95% CI [0.02-3.10] | -0.916  (0.660) 95% CI [-2.20-0.37] |
| Centred proportion of demonstrators choosing % X uninformative-different dummy | 1.023 (0.794)  95% CI [-0.53 -2.57] | -1.336 * (0.635) 95% CI [-2.57- -0.10] |
| Centred proportion of demonstrators choosing % X reliably correct-same dummy | 1.783 * (0.763)  95% CI [0.29-3.27] | -0.318  (0.690) 95% CI [-1.66-1.03] |
| Centred proportion of demonstrators choosing % X reliably correct-different dummy | 0.690 (0.759) 95% CI [-0.79 - 2.17] | -0.736 (0.564) 95% CI -1.84-0.36] |
| Percentage as optimal dummy [signal indicates percentage is optimal option] | -0.136  (0.106) 95% CI [-0.34 -0.07] | 0.145 (0.112) 95% CI [-0.07-0.36] |
| Age | -0.015 (0.036) 95% CI [-0.09 -0.06] | 0.039 (0.045) 95% CI [-0.05 -0.13] |
| Gender | -0.007 (0.210) 95% CI [-0.42-0.40] | 0.180 (0.192) 95% CI [-0.19-0.55] |
| Time in residence | 0.004 (0.015) 95% CI [-0.02-0.03] | 0.008 (0.133) 95% CI [-0.02 -0.03] |
| Block Index | -0.066 *** (0.009) 95% CI [-0.08- -0.05] | -0.051 *** (0.010)  95% CI [-0.07- -0.03] |
| India dummy | 0.529 ** (0.164)  95% CI [0.21-0.85] | - |

*The asterisks denote the level of significance of our p values, with the following key:
*** (p<0.001)
** (p<0.01)
* (p<0.05)****^.^*** *(trend: p=0.05 – 0.10 significance)*

**S2: Repeating the logistic regressions for the coordination game with the crashed session removed**Due to a crash in one experimental session, only 278 participants (116 social learners) managed to provide demographics during the coordination game. The analysis reported in our main text uses the full data for power, though we repeat all analyses with the crashed session removed here for transparency reasons. All of the regressions reported in the supplementary text below only use the data for the 116 social learners that completed the full coordination game sessions. Any discrepancies with the analysis reported in our main text is discussed throughout.

**S2A: Repeating the logistic regression modelling whether the demonstrators chose the demonstrator optimum, with dummy for the final period and % as optimal predictors.**
Robust standard errors clustered on the demonstrator. This analysis confirms that the demonstrators did reliably learn to coordinate on the optimal option during the coordination game, when the crashed session was removed though they also had an arbitrary preference to coordinate on the % option. This matches the logistic regression reported in-text. This means that the demonstrators reliably learned to coordinate on the optimal option during the coordination game in both full and crashed experimental sessions.

| Parameter | Estimate (Coordination game, crash removed) |
| --- | --- |
| Intercept | -0.165 (0.178) 95% CI [-0.51 - 0.18] |
| Final Period dummy | 0.359 *** (0.101) 95% CI [0.16-0.56] |
| % as optimal | 1.30 *** (0.30) 95% CI [0.72-1.88] |

*The asterisks denote the level of significance of our p values, with the following key:
*** (p<0.001)
** (p<0.01)
* (p<0.05)****^.^*** *(trend: p=0.05 – 0.10 significance)*

**S2B: Repeating the logistic regression modelling social learner flexibility for the coordination game with crashed data removed.**
This analysis modelled whether the social learners chose % on the coordination game with 116 participants (crashed session removed). Predictors included: (i) the centered proportion of demonstrators choosing % on each block, (ii) each combination of similarity and reliability information minus the omitted category of reliably incorrect-similar signals and (iii) interactions between each of the dummies and the centered proportion of demonstrators who chose %. The robust standard errors given in parentheses were clustered on the social learner to account for the multiple observations gathered per learner.

Note that the significant betas in Table 2B match those reported in Table 3 in-text. That is, removing the crashed session did not affect the analysis; the participants in the crashed session versus the full session were equally flexible when making their choices during the coordination game.

| Parameter | Estimate (crashed coordination game) |
| --- | --- |
| Intercept | 0.346 * (0.140)  95% CI [0.07-0.62] |
| Centred proportion of demonstrators choosing % | -1.610 ** (0.530) 95% CI [-2.65- -0.57] |
| Reliably incorrect-different dummy [signal indicates different and is correct with 0.1 probability] | -0.229 (0.206) 95% CI [-0.63-0.17] |
| Uninformative-same dummy [signal indicates same and is correct with 0.5 probability] | 0.041 (0.189)  95% CI [-0.33-0.41] |
| Uninformative-different dummy [signal indicates different and is correct with 0.5 probability] | -0.224 (0.173) 95% CI [-0.56-0.11] |
| Reliably correct-similar dummy [signal indicates same and is correct with 0.9 probability] | -0.264 (0.220) 95% CI [-0.70-0.17] |
| Reliably correct-different dummy [signal indicates different and is correct with 0.9 probability] | -0.033 (0.826)  95% CI [-0.41-0.34] |
| Centred proportion of demonstrators choosing % X reliably incorrect-different dummy | 3.381 *** (0.826)  95% CI [1.77-4.99] |
| Centred proportion of demonstrators choosing % X uninformative-same dummy | 4.037 *** (0.808)  95% CI [2.46-5.62] |
| Centred proportion of demonstrators choosing % X uninformative-different dummy | 1.877 ** (0.728)  95% CI [0.45-3.30] |
| Centred proportion of demonstrators choosing % X reliably correct-same dummy | 6.974 *** (1.074)  95% CI [4.88-9.07] |
| Centred proportion of demonstrators choosing % X reliably correct-different dummy | -0.366 (0.694)  95% CI [-1.72-0.99] |

*The asterisks denote the level of significance of our p values, with the following key:
*** (p<0.001)
** (p<0.01)
* (p<0.05)****^.^*** *(trend: p=0.05 – 0.10 significance)*

**S2C: Repeating the logistic regression predicting social learner optimality for the coordination game with the crashed data removed.**The social learners may have adjusted their choices symmetrically by responding optimally to all informationally-equivalent trials (i.e., reliably correct, and reliably incorrect signals of similarity or difference). Alternatively, the social learners may show asymmetric adjustments if they find some of the informationally-equivalent trials easier to respond to than others. Accordingly, this logistic regression models whether the social learners chose the social learner optimum for the coordination game with the crashed session removed (for 116 participants only). Predictors included: (i) the centered proportion of demonstrators who chose the demonstrator optimum, (ii) dummies for each combination of similarity and reliability information, minus the omitted category of reliably incorrect- similar signals, and (iii) interactions between each of these dummies and the centered proportion of demonstrators who chose the demonstrator optimum. Robust standard error clustered on social learner.

When the blocks gave incorrect similarity signals (right-hand column in Table 2C), the social learners were more likely to coordinate on their social learner optimum if more of the demonstrators chose optimally (centered proportion of demonstrators choosing the demonstrator optimum beta). The social learners were less likely to coordinate on the social learner optimum as more demonstrators chose optimally under the following blocks: uninformative-similar and reliably-correct similar. Note that these betas match those reported in appendix 13 of the main text, showing consistency between how the coordination game was learned across the full data-set and the crashed data-set. However, the beta for centered proportion of demonstrators choosing optimum X reliably correct-different interaction is no longer significant when the crashed session is removed.

When the blocks gave correct similarity information (middle column of Table 2C), the social learners were less likely to coordinate on the social learner optimum for blocks with uninformative-similar signals (though the beta for reliably incorrect-different signals is no longer significant when the crashed session data is removed). The learner is also more likely to answer optimally as more demonstrators coordinate on their demonstrator optimum for the following blocks: uninformative-similar, reliably correct-similar and reliably correct-different. These match the betas in appendix 13 of the main text, again confirming consistency between how the coordination game was learned across both the full and crashed sessions.

When looking at all blocks- correct and incorrect- together (left-hand column of Table 2C), an interesting finding emerges. The social learners were more likely to coordinate on their social learner optimum on reliably correct-similar blocks. This does not match Table 5 in-text, where the social learners actually became less likely to coordinate on the optimal option as mote demonstrators chose optimally for uninformative-similar blocks. This highlights the inconsistent nature of the social learners’ bias to learn social norms from reliably similar others. Those participants in the crashed session may have arbitrarily copied similar others to improve the chances of coordination (even if on the sub-optimal option) due to the pressures of the coordination game, whereas the social learners in the full sessions may have learned to up weigh the social information from reliably similar others throughout the game, similar to how the social learners played the game against nature in-text.

| Parameter | Estimate (coordination game, all signals) | Estimate (coordination game, correct signals) | Estimate (coordination game, wrong signals) |
| --- | --- | --- | --- |
| Intercept | 0.009 (0.121)  95% CI [-0.23-0.25] | 0.096 (0.076) 95% CI [-0.05-0.24] | 0.061 (0.075) 95% CI [-0.08-0.21] |
| Centred proportion of demonstrators choosing the demonstrator optimum | 0.766 (0.471) 95% CI [-0.16-1.69] | 0.125 (0.232) 95% CI [-0.33-0.58] | 1.510 *** (0.307) 95% CI [0.91-2.11] |
| Reliably incorrect-different dummy [indicates different and is correct with 0.1 probability] | 0.125 (0.179)  95% CI [-0.22-0.47] | 1.125 . (0.672) 95% CI [-0.19-2.44] | 0.033 (0.159) 95% CI [-0.28-0.34] |
| Uninformative-similar dummy [indicates same and is correct with 0.5 probability] | -0.087 (0.181)  95% CI [-0.44-0.27] | -0.593 ** (0.221) 95% CI [-1.02--0.16] | -0.284 (0.220) 95% CI [-0.71-0.15] |
| Uninformative-different dummy [indicates different and is correct with 0.5 probability] | 0.016 (0.177)  95% CI [-0.33-0.36] | -0.256 (0.220) 95% CI [-0.69-0.17] | 0.166 (0.224) 95% CI [-0.27-0.60] |
| Reliably correct-same dummy [indicates same and is correct with 0.9 probability] | 0.723 ** (0.254)  95% CI [0.23-1.22] | 0.216 (0.247) 95% CI [-0.27-0.70] | -0.289 (0.316) 95% CI [-0.33-0.91] |
| Reliably correct-different dummy [indicates different and is correct with 0.9 probability] | 0.029 (0.181)  95% CI [-0.33-0.36] | -0.039 (0.145) 95% CI [-0.32-0.25] | -0.465 (1.044) 95% CI [-1.58-2.51] |
| Centred proportion of demonstrators choosing optimum X reliably incorrect-different dummy | 0.887 (0.646)  95% CI [-0.38-2.15] | -3.533 . (2.038) 95% CI [-7.52-0.45] | 0.374 (0.558) 95% CI [-0.72-1.46] |
| Centred proportion of demonstrators choosing optimum X uninformative-same dummy | -1.046 . (0.604) 95% CI [-2.23-0.14] | 3.950 *** (0.847) 95% CI [2.29-5.61] | -4.435 *** (0.824) 95% CI [-6.05--2.82] |
| Centred proportion of demonstrators choosing optimum X uninformative-different dummy | -0.538 (0.639) 95% CI [-1.79-0.71] | -0.022 (0.735) 95% CI [-1.46-1.42] | -1.183  (0.932) 95% CI [-3.01 - 0.64] |
| Centred proportion of demonstrators choosing optimum X reliably correct-same dummy | 0.248 (0.829) 95% CI [-1.37-1.87] | 4.554 *** (1.076)  95% CI [2.45-6.66] | -7.107 *** (1.684) 95% CI [-10.40- -3.82] |
| Centred proportion of demonstrators choosing optimum X reliably correct-different dummy | 0.353 (0.626)  95% CI [-0.87-1.58] | 1.181 * (0.565) 95% CI [0.08-2.29] | -4.728 (2.837) .  95% CI [-10.27-0.82] |

*The asterisks denote the level of significance of our p values, with the following key:
*** (p<0.001)
** (p<0.01)
* (p<0.05)****^.^*** *(trend: p=0.05 – 0.10 significance)*
